# Early Economic Modeling to Inform a Target Product Profile: A Case Study of a Novel Rapid Test for *Clostridioides difficile* Infection

**DOI:** 10.1177/23814683241293739

**Published:** 2024-11-22

**Authors:** Paola Cocco, Alison Florence Smith, Kerrie Ann Davies, Christopher Michael Rooney, Robert Michael West, Bethany Shinkins

**Affiliations:** Academic Unit of Health Economics, Leeds Diagnosis and Screening Unit, Leeds Institute for Health Sciences, University of Leeds, Leeds, UK; Academic Unit of Health Economics, Leeds Diagnosis and Screening Unit, Leeds Institute for Health Sciences, University of Leeds, Leeds, UK; Academic Unit of Health Economics, Leeds Diagnosis and Screening Unit, Leeds Institute for Health Sciences, NIHR Leeds In Vitro Diagnostics Co-operative (MIC), University of Leeds, Leeds, UK; Academic Unit of Health Economics, Leeds Diagnosis and Screening Unit, Leeds Institute for Health Sciences, NIHR Leeds In Vitro Diagnostics Co-operative (MIC), University of Leeds, Leeds, UK; Healthcare Associated Infections Research Group, Leeds Teaching Hospitals NHS Trust, and University of Leeds, Leeds, UK; NIHR Leeds In Vitro Diagnostics Co-operative (MIC), Leeds Teaching Hospitals NHS Trust, and University of Leeds, Leeds, UK; European Society of Clinical Microbiology and Infectious Diseases (ESCMID) Study Group for Clostridioides difficile – ESGCD; Healthcare Associated Infections Research Group, Leeds Teaching Hospitals NHS Trust, and University of Leeds, Leeds, UK; Leeds Institute for Health Sciences, University of Leeds, Leeds, UK; Division of Health Sciences, Warwick Medical School, University of Warwick, Coventry, UK

**Keywords:** early economic evaluation, target product profiles, diagnostic test, *clostridioides difficile* infection, test evaluation pathway

## Abstract

**Background.** Target product profiles (TPPs) specify the essential properties tests must have to be able to address an unmet clinical need. **Aim.** To explore how early economic modeling can help to define TPP specifications based on cost-effectiveness considerations using the example of a new rapid diagnostic for *Clostridioides difficile* infection (CDI), a contagious health care–associated infection causing potentially fatal diarrhea. **Methods.** A resource-constrained simulation model was developed to compare a hypothetical test for CDI with current practice (i.e., test with glutamate dehydrogenase enzyme immunoassay first; if positive, test with polymerase chain reaction and cytotoxicity assay) for adult individuals with suspected CDI at the Leeds Teaching Hospital National Health System (NHS) Trust in the United Kingdom. Parameters are taken from UK-based observational data collected between 2018 and 2021, published literature, and expert opinion. A methodological framework was developed 1) to derive minimum diagnostic sensitivity and specificity and maximum price for different test turnaround-time values based on cost-effectiveness considerations from the health care perspective using the National Institute of Health Care Excellence willingness-to-pay threshold of £20,000 per quality-adjusted life-years and 2) to test their robustness using a series of sensitivity analyses. **Results.** A new rapid test for CDI with a 15-min turnaround time would require a minimum diagnostic sensitivity and specificity both equal to 96% and a maximum price of £44 to maintain cost-effectiveness compared with standard of care. **Conclusions.** This study provides a framework to inform the essential test properties based on cost-effectiveness considerations and to isolate the most influential model parameters and scenarios via a series of sensitivity analyses. These specifications, in turn, could be used to inform future TPPs for tests.

**Highlights:**

- Target product profiles (TPPs) for new medical tests provide test developers with performance benchmarks and technical requirements for new tests. Early economic evaluation has already been used to identify acceptable ranges for certain performance requirements for new tests. Currently, however, early economic evaluation methods are yet to be used in the context of TPP development, and there is no guidance as to how this could and should be done.
- A de novo approach was developed to identify the minimum performance requirements and maximum costs for new tests, based on cost-effectiveness considerations, while also isolating most influential parameters. The added value of this framework lies in structuring early economic evaluation methods as a means of informing transparent, evidence-based minimum TPP performance specifications while also accounting as much as possible for the (inevitable) uncertainty surrounding the minimum performance requirements.
- This study represents the first application of early economic modeling as a means of deriving the minimum performance specifications for a novel point-of-care test for *Clostridioides difficile* infection as set out in a future TPP.

Target product profiles (TPPs) for diagnostic tests describe, at the early stages of technology development, the operational and performance requirements that novel tests would need to address an unmet clinical need.^1,2^ Existing TPPs for tests have provided specifications on 1) analytical performance (e.g., sample requirements), 2) clinical validity (e.g., diagnostic accuracy), 3) infrastructural requirements (e.g., transportation), 4) human factors (e.g., training needs), and 5) costs.^2^ Typically, test requirements are presented at 2 levels (“desirable” and “acceptable”), based on expert consultations and literature findings.^2^

Published TPPs for tests have largely been developed for global health applications in the context of low- or middle-income countries, with a sole focus on infection diagnostics.^2^ The main organizations funding the development of TPPs have been the World Health Organization and the Bill and Melinda Gates Foundation,^3^ in a bid to stimulate research and development on novel diagnostics.^3^ Increasing recognition that most tests currently developed fail to reach clinical practice^4^ has stimulated interest in TPPs,^3,5^ especially in response to the COVID-19 pandemic. For example, the UK Medicines & Healthcare products Regulatory Agency (MHRA) developed TPPs to assist manufacturers to design “fit-for-purpose” COVID-19 tests.^3^

We conducted a systematic review of published TPPs and identified a number of methodological limitations, including a lack of consideration as to how a test affects health outcomes and cost-effectiveness when defining test requirements, in addition to poor transparency in methodology reporting and a reliance on subjective data.^2^ Before embarking on developing TPPs for different clinical contexts, it is vital that the methodology be improved. Early economic evaluation (EEE) compares the costs and benefits of alternative interventions and is typically performed at early stages of technology development.^6^ We proposed that EEE could be a vehicle for identifying the necessary features of a new test and understanding the dependencies between test features, while considering cost-effectiveness.^7^

The aim of this study is to explore how early economic modeling can help to define TPP specifications using the example of a novel rapid test for *Clostridioides difficile* infection (CDI). CDI is one of the leading causes of health care–associated infections, with symptoms ranging from mild diarrhea to fulminant colitis. In England, inpatients with suspected CDI are isolated in single rooms while awaiting test results to prevent in-hospital transmission, until confirmation of noninfectious diarrhea.^8^

Based on the findings of an online survey of UK health care professionals with experience of CDI (*n* = 48), which aimed to assess current clinical practices for diagnosing CDI and any issues associated with the diagnosis of CDI, a perceived need for more accurate and rapid new point-of-care test (POCT) for CDI emerged.^9^ In addition to this, to overcome the limitations of current diagnostics for CDI,^10^ a new POCT for CDI is currently under development as part of a Medical Research Council–funded program grant (MR/N029976/1). To ensure that the test is fit for purpose, this program includes the development of a TPP.

To this end, we developed an early economic model to identify the necessary properties for a rapid hypothetical test (henceforth “HT”) for CDI to be cost-effective compared with standard care (focusing on minimum diagnostic sensitivity and specificity, turnaround time, and maximum test cost). The findings from this EEE will provide test developers with a blueprint of required characteristics a rapid CDI test should possess and, ultimately, will be used at later stages to inform parts of the full TPP developed within the Medical Research Council–funded project.

## Methods

### Model Structure

We developed a deterministic, stochastic, resource-constrained discrete event simulation (DES) in SIMUL8 to explore what impact a hypothetical rapid test could have on the availability of free single rooms and infection spread. The primary advantages of DES modeling in this case are^11^ 1) the ability to capture individual patient characteristics and history, 3) the ability to measure the timings of events, 3) the ability to simulate a sequence of hospital processes within the care pathway, and 4) the ability to capture capacity constraints for scarce resources (e.g., single rooms) and queues in the system.

The model reflects the clinical pathway in place at Leeds Teaching Hospital National Health System (NHS) Trust (LTHT), a medium to large NHS hospital.^12,13^ There is a wide variation in testing practices for diagnosing CDI in England,^14^ and therefore, a pragmatic decision was taken to simulate a clinical pathway specific to 1 site.

**Figure 1:**
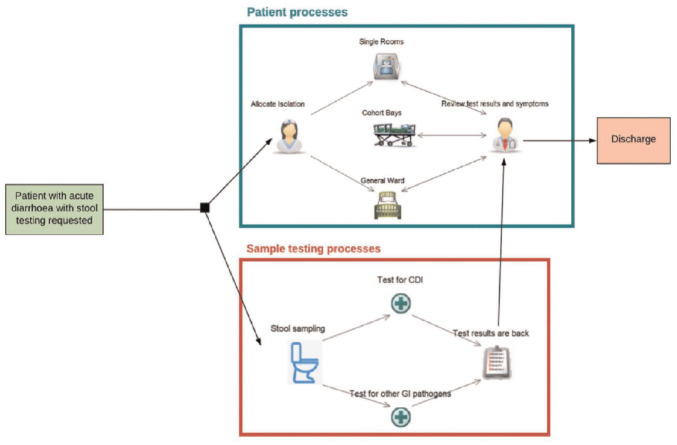
Simplified schematic of the early economic model for a new hypothetical rapid diagnostic test for *Clostridioides difficile* infection.

Consultations with clinical experts based at LTHT and a review of national and local clinical guidelines informed the model structure, presented in Figure 1,^9,12,13,15^ and model assumptions (see Table 1). The model tracks the flow of inpatients from the point of symptom presentation to hospital discharge. Adult inpatients with an initial episode of diarrhea for whom clinicians have requested stool testing for CDI^i^ enter the model. Two locations are available for patients suspected with infectious diarrhea, depending on room availability: 1) single isolation rooms, used to reduce the risk of infection spread, and 2) the general ward, used in scenarios in which no single rooms are available, presenting a higher risk of transmission. Patients remain in single room isolation or the general ward depending on their assigned length of stay (LOS). Details of the model are provided in Appendix 1.

**Table 1:** Key Model Assumptions

Clinical Assumptions
1. The monthly demand for stool testing was assumed to be constant and independent from seasonal change or other external factors.
2. There is no risk of co-infection between GI pathogens and CDI.
3. Isolation in single rooms (or cohort bays) is required for patients positive for other GI pathogens until 2 d after symptoms resolution (i.e., resolution of diarrhea).
4. When single rooms and cohort bays are at full capacity, patients confirmed with infectious diarrhea remain in the general ward until hospital discharge.
5. Upon confirmation of infectious diarrhea, patients in the general ward can be transferred into 1 of the 4 cohort bays depending on current availability. Patients confirmed with CDI are grouped separately from patients positive for other GI pathogens, to reduce the risk of developing co-infections. Confirmed patients remain in cohort bays until hospital discharge.
6. A rapid multiplex GI panel with assumed perfected diagnostic accuracy is run to detect multiple pathogens; this is separate from tests for CDI. Perfected diagnostic accuracy was assumed for the multiplex GI panel as the focus of this decision model lies in evaluating testing strategies for CDI, rather than other GI pathogens.
7. Clinicians consider the continuation or resolution of diarrhea as the only symptom for CDI, without assessing whether the frequency of stools is improving or worsening.
8. Clinicians are assumed to preemptively administer treatment for CDI (i.e. “empirical treatment”) prior to receipt of test results if a patient presents with severe symptoms of CDI, as per current clinical practice at LTHT.
9. Clinicians fully adhere to test results when deciding when to start or stop administering antibiotic treatment for CDI.
10. Early treatment, via early diagnosis, is assumed to have no impact on patient survival, risk of disease recurrence, or long-term quality of life due to paucity of data.
11. Patients with a TP result for CDI are assumed to recover to full health without risk of disease recurrence at the end of the antibiotic treatment regimen.
12. Patients with an FN result for CDI do not experience further disease recurrences.
13. Patients with an FN result for CDI are assumed to miss antibiotic prescriptions for CDI, and this, in turn, increases the risk of health-related quality-of-life loss due to missed treatment. In addition, FN patients are assumed to be released from single room isolation into the general ward upon receipt of an (incorrect) negative result, which, in turn, increases the risk of nosocomial transmission of CDI within the general ward.
14. Patients with an FP result for CDI are assumed to remain symptomatic at day 10 of treatment and to receive treatment for 4 additional days, as per current clinical practice at LTHT. This in turn reduces the availability of free single rooms for suspected or confirmed infectious patients, thus increasing the likelihood of infection spread in the general ward. Since patients with an FP test result receive unnecessary antibiotic treatment for CDI, they are assumed to remain symptomatic at day 10 of treatment as they are not treated for the real cause of diarrhea (e.g., noninfectious condition, as per expert opinion). This increases the loss in health-related quality of life due to receiving incorrect antibiotic treatment.
15. An indirect approach is taken to capture the impact of possible CDI infection spread within the general ward only, based on calculating the number of new secondary cases using the reproductive rate of infection spread (i.e., an estimate of the number of secondary infected cases resulting from 1 primary infected patient entering the general ward). The number of primary infected cases in the general ward comprises 1) FN cases incorrectly placed into general ward and 2) TP cases placed into the general ward due to paucity of free single rooms and cohort bays.

CDI, *Clostridioides difficile* infection; FN, false negative; FP, false positive; GI, gastrointestinal infection; LTHT, Leeds Teaching Hospital National Health System (NHS) Trust; TN, true negative; TP, true-positive.

The model compares an HT for CDI against standard diagnostic care. At LTHT, a 2-step testing algorithm (i.e., combination of sequential tests) is run for patients with suspected CDI: glutamate dehydrogenase (GDH) enzyme immunoassay (EIA) testing, with positive results confirmed by polymerase chain reaction and cell-cytotoxicity neutralization assay. Note that this testing algorithm is particular to LTHT, as cell cytotoxicity is rarely used; instead, GDH/toxin A/B EIA is the most common algorithm for CDI across English NHS hospitals.^14^ All samples are simultaneously tested for CDI and other gastrointestinal (GI) pathogens (e.g., norovirus, rotavirus, *Campylobacter*) using a rapid multiplex GI panel assumed to have perfect diagnostic accuracy.

The model captures the impact of the new POCT with increased diagnostic accuracy and reduced turnaround time on 1) improving short-term clinical outcomes (e.g., enhanced effectiveness of antibiotic treatment for CDI, reduced LOS) for patients via an expedited (and appropriate) administration of antibiotics for CDI and 2) supporting fast and appropriate (de-)escalation of infection-control measures to minimize in-hospital transmission. On a system-related level, a new test capable of reducing turnaround time shortens patients’ stay in presumptive isolation while awaiting the confirmation of infectious diarrhea as well as reducing the LOS for patients positive for CDI. This results in more single rooms being available for new patients suspected with CDI, thus reducing the number of secondary CDI cases in the general ward.^14^

Consultations with clinical experts ensured the face validity of the model structure and parameters. Coding was internally reviewed. The model was developed in accordance with the Strengthening The Reporting of Empirical Simulation Studies guidelines^16^ and Consolidated Health Economic Evaluation Reporting Standards^17^ (see Appendix 2).

### Data Sources for Model Parameters

Table 2 provides an overview of model parameters and related sources. Parameters relating to patient characteristics (e.g., symptoms duration, LOS) were based on UK-specific summary estimates from the point-prevalence study part of the Combatting Bacterial Resistance in Europe (COMBACTE-CDI) study (*n* = 180 patients).^18^

**Table 2:** Model Parameters, Related Data Sources and Notes, and Distribution Type

Parameter	Estimate (*s*)	Distribution Type	Data Source
Disease-related parameters			
CDI disease prevalence	10.3%	Probability profile^a^	Freeman et al.^19^
Other GI pathogen prevalence	13.8%	Probability profile	Freeman et al.^19^
CDI patients with mild symptoms	39.0%	Probability profile	COMBACTE-CDI CRF dataset
CDI patients with moderate symptoms	33.0%	Probability profile	COMBACTE-CDI CRF dataset
CDI patients with severe symptoms	27.0%	Probability profile	COMBACTE-CDI CRF dataset
Length of stay for CDI-negative patients (d)	28.6 (38.5)	Weibull	COMBACTE-CDI CRF dataset
Duration of diarrhea after sampling CDI-negative patients (d)	7.041 (12.0)	Weibull	COMBACTE-CDI CRF dataset
Duration of diarrhea after sampling CDI-positive patients (d)	12.27 (26.0)	Weibull	COMBACTE-CDI CRF dataset
UK England utility weight general population aged 55–64 y	0.8	Fixed	Szende et al.^20^
UK utility weight adult hospitalized patient with first episode of CDI	0.4	Fixed	Wilcox et al.^21^
Decrement utility weight due to inappropriate antibiotic treatment	0.1	Fixed	Assumption
Duration of CDI disease	10–14 d	Fixed	Freeman et al.^19^ and McDonald et al.^22^
Duration of CDI clinical management	30 d	Fixed	Barbut et al.^23^ and Jones et al.^24^
Reproductive ratio for CDI (median)	1.0	Fixed	Lanzas et al.^25^
Probability of clinical cure, slow diagnosis	85.3%	Probability profile	Barbut et al.^23^
Probability of clinical cure, average diagnosis	90.7%	Probability profile	Barbut et al.^23^
Probability of clinical cure, rapid diagnosis	95.6%	Probability profile	Barbut et al.^23^
Length of stay of CDI-positive patients, slow diagnosis (mean number of days)	30.3 (36.3)	Weibull	Barbut et al.^23^
Length of stay of CDI-positive patients, average diagnosis (mean number of days)	26.9 (28.9)	Weibull	Barbut et al.^23^
Length of stay of CDI-positive patients, rapid diagnosis (mean number of days)	23.2 (25.4)	Weibull	Barbut et al.^23^
Diagnostic accuracy			
GDH EIA sensitivity	94.0%	Fixed	Crobach et al.^10^
GDH EIA specificity	94.0%	Fixed	Crobach et al.^10^
PCR sensitivity	95.0%	Fixed	Crobach et al.^10^
PCR specificity	98.0%	Fixed	Crobach et al.^10^
CCNA sensitivity	86.4%	Fixed	Planche and Wilcox^26^ and Bank of England^27^
CCNA specificity	99.2%	Fixed	Planche and Wilcox^26^ and Bank of England^27^
Multiplex GI panel testing sensitivity	100%	Fixed	Assumption
Multiplex GI panel testing specificity	100%	Fixed	Assumption
Testing workflow			
Monthly median stool samples tested in UK teaching hospitals (*n*)	1,430.5 (689)	Fixed	COMBACTE-CDI survey
Proportion of samples tested for CDI only	5%–69%	Fixed	COMBACTE-CDI survey
Time to obtain stool sample	0.5 (0–2 d)	Triangular	Jones et al.^24^
Time to transport sample to the laboratory	15 min	Fixed	Assumption
Techlab *C diff* check GDH EIA operating time	60 min	Fixed	Techlab^28^
Xpert *C. difficile* BT Cepheid PCR operating time	43 min	Fixed	Cepheid^29^
CCNA operating time (d)	1.5 (1–2)	Triangular	Expert opinion
Multiplex GI pathogens panel operating time	43 min	Fixed	Equal to PCR
Time for preparing sample batch	30 min	Normal	Expert opinion
Xpert *C. difficile* BT Cepheid PCR operating time	1 min	Fixed	Cepheid^30^
Time for preparing CCNA	20 min	Fixed	Expert opinion
Time to review positive test results	30 min	Fixed	Expert opinion
Time to review negative test results	0 min	Fixed	Expert opinion
Costs			
Cost of bed day in adult isolation	£692.8	Fixed	Jones et al.,^24^ estimate dated 2016
Cost of bed day in general ward	£583.0	Fixed	Jones et al.,^24^ estimate dated 2016
GDH EIA cost per kit	£4.8	Fixed	Jones et al.,^24^ estimate dated 2017
PCR cost per run	£26.9	Fixed	Jones et al.,^24^ estimate dated 2017
CCNA cost	£3.7	Fixed	Sewell et al.,^31^ estimate dated 2011
Multiplex GI panel cost per sample	£43.0	Fixed	Jones et al.,^24^ estimate dated 2017
Vancomycin 125 mg (PO every 6 h for 10 d)	£132.5	Fixed	British National Formulary,^32^ estimate dated 2021
Additional cost per secondary case per day	£957.2	Fixed	Tresman and Goldenberg,^33^ estimate dated 2017
Hospital configuration			
Single rooms in typical UK teaching hospital (*n*)	93	N/A	COMBACTE-CDI survey

CCNA, cell cytotoxicity neutralization; CDI, *Clostridioides difficile* infection; EIA, enzyme immunoassay; GDH, glutamate dehydrogenase; GI, gastrointestinal infection; PO, per os (i.e., orally); PCR, polymerase chain reaction.

a A probability profile in SIMUL8 is defined as “a type of distribution that sets the probability (the percentage change) of a value being sampled from a distribution.”^28.34^

COMBACTE-CDI was a multicenter European-wide epidemiological study assessing the impact of CDI on community and hospitalized patients’ health and clinical practice across 119 health care sites from 12 European countries, conducted from 2018 to 2021.^35,36^ In addition, UK-specific summary data from a European-wide survey part of COMBACTE-CDI study was used to inform key model parameters related to hospital configuration (e.g., numbers of samples run and single rooms available). This survey was sent out to community and hospital sites (*n* = 158) across 12 European countries to assess clinical practices for CDI patients and costs.^37^

Diagnostic accuracies for standard care were based on published estimates, taking toxigenic culture as a reference method.^10,26,27^ Independence between sequential tests was assumed due to the paucity of data on the comparative diagnostic accuracy of each test. Health-related utility weights associated with CDI were based on a UK-based prospective study conducted with 30 UK adult inpatients with a first CDI episode (median age 70.2 y, 27% of patients had severe CDI).^21^

As per the National Institute for Health and Care Excellence (NICE) reference case, the analysis adopted the UK NHS and Personal Social Services perspective and included direct health care costs, such as direct testing costs, CDI treatment costs, bed costs, and additional costs due to secondary infections. Direct health care costs were based on published data. Costs were inflated to 2021 prices using the Bank of England inflator where appropriate.^38^ Other parameters are based on literature and expert opinion (see Appendix 1).

### Outcomes

The clinical effectiveness of each strategy was measured in terms of 1) quality-adjusted life-year (QALY) gains and 2) the number of secondary infections prevented (i.e., newly infected individuals resulting from contact with infected patients in the general ward). Since CDI is a transient disease, QALYs were estimated by first calculating the quality-adjusted life-days lost due to CDI and then converting this into QALYs lost.^17,39^ Details on this stage are provided in Appendix 1, section 1.3.

Cost-effectiveness outputs were expressed in terms of incremental net monetary benefit (INMB) from the UK NHS and Personal Social Services perspective, comparing HT against standard care, using the NICE willingness-to-pay (WTP) per QALY threshold of £20,000.

### Model Analysis

A warm-up period of 45 d was applied in the model to capture the steady-state capacity constraints patients face upon entering a busy hospital. Within constrained-DES models with a constant flow of patients, the warm-up period refers to a specified time period prior to starting the analysis that is applied to simulate the typical single room occupancy and operational conditions that are representative of a normal operational day within the hospital system.^40^ Starting with the model “cold” (i.e., all single rooms available and no patients in the system) would misrepresent the hospital stable operational condition and overestimate the hospital’s capacity to isolate patients.

After the warm-up period, the model records outputs for every patient within the model evaluation set (i.e., those entering with the model entry period, which is set equal to 60 d following the end of the warm-up period). Upon completion of the warm-up period, the model runs for a time horizon of 7 mo to ensure that every patient within the evaluation set can have their full experience of the clinical pathway simulated. Although the presented model is deterministic, all analyses were based on running 70 model replications (i.e., running the model 70 times using the same deterministic estimates for model parameters, with each run using a different random number) to minimize the impact of first-order uncertainty^ii^ on the results.^41^

As per good modeling practices,^40^ the warm-up period and replication number were set to values sufficient to provide stable outputs for the number of secondary infections within the general ward (i.e., less than 1% difference observed between output values across increasing model replications). Given the short time horizon, a discount rate of 0% was applied.

The aim of the analysis was to identify the minimum acceptable performance specifications for HT with respect to 1) diagnostic sensitivity, 2) diagnostic specificity, and 3) the maximum test cost for different test turnaround-time values. To do this, we developed a 3-stage minimum performance specifications framework (henceforth “MPS framework”; see Table 3). Next, we conducted extensive deterministic sensitivity analyses to identify the drivers of cost-effectiveness changes among the model parameters and assess how the results of the MPS framework would change depending on different values of the key drivers.

**Table 3:** Overview of the Minimum Performance Specification (MPS) Framework

Stage	Aim(s)	Methods
1, Assessment of clinical effectiveness outputs	To exclude any region of diagnostic accuracy resulting in clinically inferior outputs	• Determine when HT is considered less clinically effective
		• Two-way sensitivity analysis varying diagnostic sensitivity and specificity
		• Hold third test performance dimension^a^ constant to best-case value
		• Assume zero testing cost for HT
2, Assessment of cost-effectiveness outputs	To exclude any region of diagnostic accuracy not able to maintain cost-effectiveness	• Three-way sensitivity analysis varying diagnostic sensitivity, specificity, and third test performance dimension^b^
		• Assume zero testing cost for HT
3, Identifying minimum test specifications and maximum acceptable prices	(1) To convert the results of the previous stages into “minimum” specifications (2) To estimate the headroom and threshold prices using the WTP threshold	• Minimum: the lowest acceptable levels of diagnostic sensitivity and specificity that maintained clinical effectiveness (stage 1) and cost-effectiveness (stage 2)
		• Headroom price: represents the maximum price for HT to be cost-effective, assuming that HT meets the perfect levels of diagnostic accuracy. sorted by third test performance dimension^a^
		• Threshold price: represents the maximum price for HT to be cost-effective based on the lowest minimal diagnostic accuracy pair as identified in stage 1 and 2, sorted by third test performance dimension^a^

HT, hypothetical test; WTP, willingness to pay.

a In the context of this model, the third test performance being simulated is test turnaround time.

b The 3-way sensitivity analysis is constrained to a prespecified set of values for test turnaround time (15, 90, and 180 min)

### MPS Framework

The MPS framework pragmatically identifies the minimum performance benchmarks for HT based on deterministic incremental clinical- and cost-effectiveness outputs (see Table 3). All parameters were held at their baseline value at this stage.

#### Stage 1: Assessment of clinical effectiveness outputs

HT was considered to be clinically inferior compared with standard care when both of the following conditions were met: 1) HT led to negative incremental QALYs and 2) HT increased the number of secondary infections. Although a technology may be clinically less effective than standard care but nevertheless present a cost-effective alternative (e.g., if sufficient cost savings are accrued), the exclusion of clinically inferior options was considered appropriate because 1) the unit price of a new POCT is expected to cost more than the standard care since the latter involves batch testing and only patients positive to the first-stage test fully undergo the testing algorithm pathway, and 2) clinically inferior strategies would not be expected to be acceptable in the context of TPP development since the overarching aim of TPPs is to provide manufacturers with the requirements novel tests would need to address an unmet clinical need.

An initial analysis ruled out any combinations of diagnostic accuracy that resulted in the HT being clinically inferior to the standard care given a rapid turnaround time. Diagnostic sensitivity and specificity pairs were varied over a pragmatic range, while assuming the quickest turnaround time for a rapid CDI diagnostic (i.e., 15 min, as per expert opinion). This allows identification of the widest region of diagnostic accuracy where the HT is expected to be more clinically effective than standard care, to take forward into the next stage of the analysis.

#### Stage 2: Assessment of cost-effectiveness outputs

At this stage, the aim was to rule out scenarios where the HT was not expected to be cost-effective assuming a zero-test cost for HT. For each diagnostic accuracy pair from stage 1, the INMB was estimated, with cost-effective being defined as “nonnegative INMB.” This was repeated for 3 test turnaround-time values (i.e., 15, 30, and 180 min).

#### Stage 3: Minimum acceptable and maximum acceptable prices

For each test turnaround-time evaluated, the performance specifications that resulted in HT being cost-effective were defined based on the results of stage 1 and 2. The “minimum” levels corresponded to the lowest values of sensitivity and specificity that maintained a positive clinical benefit (stage 1) and cost-effectiveness (stage 2). Headroom price represents the maximum price for an intervention to be cost-effective, assuming that the intervention achieves best-case values for diagnostic accuracy. Based on the minimum performance specifications for diagnostic accuracy identified from stage 2, a maximum price for HT (i.e., threshold price) to be cost-effective was estimated. The headroom and threshold unit prices were estimated using the WTP threshold of £20,000 per QALY gained.

### Sensitivity and Scenario Analyses

Minimum performance requirements are likely to vary depending on different values of model parameters or structural factors relating to the clinical context. Here, we identified the key drivers of cost-effectiveness to inform which parameters and scenarios should be prioritized for sensitivity analyses. Deterministic univariate sensitivity and scenario analyses were conducted to explore the impact of varying each of the model parameters and several scenarios on cost-effectiveness outputs. Analyses were run assuming a 15-min test turnaround time,^iii^ fixing the diagnostic accuracy values to the minimum performance specifications identified in stage 1, and fixing the test price at the threshold value. Details on this stage are provided in Appendix 3.

After running 1-way sensitivity analyses and scenario analyses, further deterministic sensitivity and scenario analyses were then run to assess the impact of the top 2 most influential parameters and scenarios on the minimum performance specifications. This, in turn, helped to draw narrative inferences on the expected trajectory of the minimum performance test specifications without rerunning the MPS framework. In the context of parameter values and scenarios in which the marginal benefit of HT increases (i.e., higher INMB than baseline results), HT would be associated with lower performance specifications and higher threshold price and maintain cost-effectiveness, compared with the respective baseline results. Conversely, in the context of scenarios that lead to a reduced marginal benefit for HT, higher minimum performance specifications and lower threshold price would be required for HT to maintain cost-effectiveness.

## Results

Across 70 model replications, the size of the cohort entering the model (i.e., evaluation set) was on average 539 patients. Note that results are presented at the aggregate level, focusing on the total number of patients part of the evaluation set.

Full results are provided in Appendix 3.

### MPS Framework

#### Stage 1: Clinical-effectiveness outputs

Assuming a 15-min turnaround time, at diagnostic specificity values of 100%, 98%, and 96%, HT was clinically inferior to standard care for diagnostic sensitivity values below 82%, 90%, and 96%, respectively (see Table 4), whereas at a specificity level of 94%, all of the results were clinically inferior to standard care regardless of the level of sensitivity chosen. Based on these results, stage 2 of the analysis focused on exploring sensitivity values between 100% and 82% and specificity values between 100% and 96%.

**Table 4:** Incremental Clinical Effectiveness (Total QALY Gains and Number of New Secondary Cases across Patients Part of the Evaluation Set) for HT at Each Diagnostic Sensitivity and Specificity Pair with a Rapid Test Turnaround Time^a^

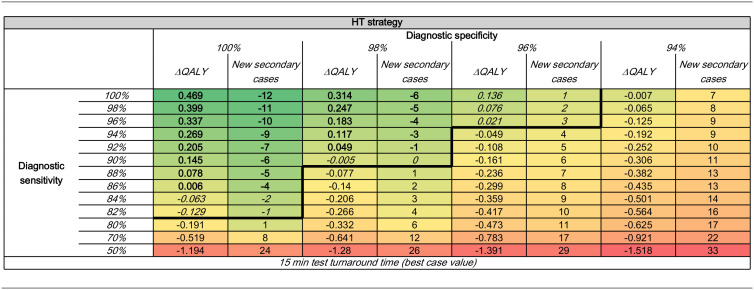

HT, hypothetical test; QALY, quality-adjusted life-year.

a Values in **bold** format represent the sensitivity specificity pairs at which HT yields higher QALY gains and leads to a lower number of new secondary infections. Values in *italic* format represent the sensitivity specificity pairs at which HT yields lower QALY gains but leads to a lower number of new secondary infections. The bold black line indicates the threshold at which the diagnostic sensitivity and specificity pairs yield better results on at least 1 of the clinical outcomes (e.g., higher ΔQALY or lower new secondary cases; i.e., HT is not clinically inferior). The cell colors provide an indication of where the results lie on the full spectrum of the outputs observed: solid green is associated with the highest clinical effectiveness outputs (e.g., higher ΔQALY and/or lower new secondary cases; i.e., dominant diagnostic sensitivity/specificity pair), whereas solid red is associated with the highest lowest clinical effectiveness outputs (e.g., lower ΔQALY and/or higher new secondary cases; i.e., dominated diagnostic sensitivity/specificity pair).

#### Stage 2: Cost-effectiveness outputs

Assuming perfect diagnostic accuracy and zero-test cost for HT, the INMB for HT was equal to £599,909 when assuming a 15-min turnaround time; £577,982 at a 90-min turnaround time; and £535,076 at a 180-min turnaround time (see Table 5).

**Table 5:** INMB (across Patients Part of the Service Evaluation Set) for HT at Each Sensitivity Specificity Pair Assuming Zero Test Cost for HT, Sorted by Test Turnaround-Time Values^a^

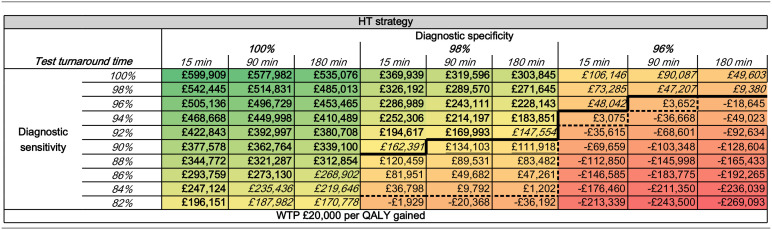

HT, hypothetical test; INMB, incremental net monetary benefit; QALY, quality-adjusted life-year; WTP, willingness to pay.

a Values in **bold** format represent the sensitivity specificity pairs at which HT yields higher QALY gains and leads to a lower number of new secondary infections and is more cost-effective. Values in *italic* format represent the sensitivity specificity pairs at which HT yields lower QALY gains but leads to a lower number of new secondary infections and is more cost-effective. Values in underline format represent the sensitivity specificity pairs at which HT is clinically inferior (i.e., DQALY and/or more new secondary cases) but maintains cost-effectiveness. The bold black line indicates the threshold at which the diagnostic sensitivity and specificity pairs yield better results on at least 1 of the clinical outcomes (e.g., higher ΔQALY or lower new secondary cases; i.e., HT is not clinically inferior) and HT is more cost-effective, sorted by test turnaround-time values. The dotted black line indicates the threshold at which the diagnostic sensitivity and specificity pairs yield worse clinical outputs on at least 1 of the clinical outcomes (i.e., HT is clinical inferior) but maintain cost-effectiveness. The cell colors provide an indication of where the results lie on the full spectrum of the outputs observed: solid green is associated with the highest cost-effectiveness outputs (e.g., higher INMB), whereas solid red is associated with the highest lowest cost-effectiveness outputs (e.g., lower INMB).

Based on the results of stage 1 and 2, for each of the following test turnaround-time values, the minimum diagnostic accuracy pairs at which HT is both clinically and cost-effective were estimated at

- 15-min turnaround time: 96% sensitivity and 96% specificity;
- 90-min turnaround time: 98% sensitivity and 96% specificity; and
- 180-min turnaround time: 98% sensitivity and 96% specificity.

#### Stage 3: Minimum test performance specifications and maximum acceptable prices

Based on the presented results for stages 1 to 3, Table 6 reports the minimum performance requirements and maximum acceptable prices associated with HT in order to achieve cost-effectiveness, for each test turnaround-time value. At a 15-min turnaround time, the minimum diagnostic sensitivity and specificity were both 96%. In the context of a test turnaround time of 90 and 180 min, the minimum sensitivity for HT to be cost-effective was 98%, whereas the minimum specificity remained 96%.

**Table 6:** Minimum Performance Specifications for HT and Maximum Acceptable Prices at Each Test Turnaround-Time Value^a^

	Test Turnaround Time		
	15 min	90 min	180 min
Minimum diagnostic sensitivity	96%	98%	98%
Minimum diagnostic specificity	96%	96%	96%
Headroom unit price (assuming optimal diagnostic accuracy) at WTP £20,000 per QALY gained	£554.36	£534.09	£494.45
Threshold unit price (assuming minimal diagnostic accuracy) at WTP £20,000 per QALY gained	£44.39	£43.62	£8.67

HT, hypothetical test; QALY, quality-adjusted life-year; WTP, willingness to pay.

a Minimum performance specifications and maximum acceptable unit price at a 90- and 180-min turnaround time are compared against minimum performance specifications and maximum unit price at a 15-min turnaround time (baseline value).

The maximum unit cost for HT to be cost-effective, however, decreased as test turnaround time increased due to a reduction in clinical effectiveness and higher costs. At a 15-min turnaround time, HT can be priced up to £544.36 assuming perfect diagnostic accuracy and still be cost-effective at a WTP threshold of £20,000. When diagnostic sensitivity and specificity were reduced to their minimally acceptable levels at a 15-min turnaround time, the maximum price for HT while maintaining cost-effectiveness was approximately £44.39.

Assuming a 90-min turnaround time, the headroom price (i.e., maximum price for a test to be cost-effective) for a perfectly accurate HT was £534.09. If diagnostic sensitivity and specificity were set to their minimum value, the threshold price for HT was £43.62. At a 180-min turnaround time and perfect diagnostic accuracy, the headroom price to be cost-effective was £494.45; once diagnostic accuracy was reduced to its minimally acceptable value, HT can be priced up to £8.67.

Full results are reported in Supplementary Table 3.5.

### Sensitivity and Scenario Analyses

Based on the results of the sensitivity and scenario analyses on the cost-effectiveness outputs associated with the minimum performance specifications for HT estimated with the MPS framework, the top 4 most influential drivers of changes were:

- operating time for multiplex GI panel: this parameter was the biggest driver of changes in cost-effectiveness, causing the INMB to vary between £255,620 and −£1,093,096 at the parameter’s lower and upper bound, respectively.
- LOS for patients positive for CDI receiving test results under slow time-to-diagnosis (i.e., ≥1.2 d): varying this parameter resulted in the INMB to fluctuate from −455,274 to £504,583 at the lower and upper bound, respectively;
- simulating a smaller hospital with fewer single rooms and patients tested for CDI: this scenario led to a higher INMB compared with baseline (£153,899); and
- increasing the availability of multiplex GI panels to test patients suspected with infectious diarrhea: this scenario yielded a higher INMB (£150,796) compared with baseline.

Additional sensitivity and scenario analyses were then conducted to explore how the minimum performance specifications at a 15-min turnaround time varied depending on the changes of the most influential model parameters and scenarios.

Applying a higher value of operating time for multiplex GI panels resulted in higher minimum performance specifications (99% for both diagnostic sensitivity and specificity) and a corresponding lower threshold unit price (£2) and headroom unit price (£179) compared with baseline values (see Table 6). Alternatively, simulating a lower value of operating time for multiplex GI panels led to an additional comparative benefit. This resulted in a comparatively lower minimum sensitivity (93%) while yielding the same required minimum diagnostic specificity as in the base-case analysis and a higher threshold (£159), and headroom unit price (£772) required for HT to maintain cost-effectiveness.

Simulating an extended LOS for patients confirmed with CDI receiving slow diagnosis resulted in a lower minimum diagnostic specificity (94%) while maintaining the same required minimum diagnostic sensitivity as in the base-case results (96%) and a corresponding higher threshold and (£310) headroom unit price (£1,031). Assuming a reduced LOS for patients led to a reduced clinical and economic benefit. This demanded a comparatively higher minimum diagnostic sensitivity (99%) and specificity (99%) for HT to be cost-effective as well as a significantly lower threshold (£10) and headroom unit price (£144).

In the scenario of a typical UK District Hospital,^iv^ the minimum performance specifications for HT were lower compared with baseline (minimum diagnostic sensitivity and specificity equal to 92% and 95%, respectively). Following the increased clinical and economic benefit of HT in this context (i.e., £153,899 INMB as estimated in phase 2), a corresponding higher threshold cost (£84) and headroom price (£2,818) were accepted in this context.

While simulating an additional multiplex GI panel, the minimum performance specifications for HT were found to be lower (90% and 93% for diagnostic sensitivity and specificity, respectively) than the baseline. The threshold cost associated with the above minimum performance specifications (£360) and headroom price (£590) was also higher than baseline.

## Discussion

### Necessary Properties of HTs for CDI

Based on the MPS framework, a new test for CDI with a 15-min turnaround time would require a minimum diagnostic sensitivity and specificity of 96% and a maximum price of approximately £44 to maintain cost-effectiveness compared with standard care. The robustness of the identified minimum performance specifications was tested against the top drivers of cost-effectiveness. Based on the results of sensitivity and scenario analyses, the minimum diagnostic sensitivity for HT varied between 90% and 99%, whereas the minimum diagnostic specificity ranged between 93% and 99%. The corresponding threshold cost ranged between £2 and £360.

Diagnostic specificity was a greater driver of cost-effectiveness compared with diagnostic sensitivity, based on the low prevalence of CDI, incorrectly diagnosing a proportion of healthy patients as CDI positive had a greater impact than missing a few truly positive CDI patients. A rapid test with lowered diagnostic specificity reduced the availability of free single rooms as more false-positive patients lead to the full capacity of the isolation rooms. This, in turn, increases the likelihood of infectious patients to be moved into the general ward, thereby leading to more secondary cases. In addition, the test turnaround time had a substantial impact on the expected benefits a POCT could afford. For example, if the test turnaround time exceeded 90 min, the incremental benefits associated with HT disappeared since the turnaround times for standard care allowed physicians to rule out CDI for most negative cases within 180 min upon sample receipt.

Minimum performance requirements for HT were high. This is largely due to the effective performance of the comparator: running testing algorithms is a cost-effective option for diagnosing CDI,^42,43^ as a sensitive first-stage test quickly rules out negative patients^39^; only positive patients fully undergo the testing pathway. New rapid diagnostics for CDI under development, nevertheless, could partially meet the estimated minimum performance requirements.^44,45^

Based on the results of sensitivity analyses, the operating time for multiplex GI panels had the biggest impact on the cost-effectiveness outputs. In clinical settings where patients suspected with infectious diarrhea are tested simultaneously for CDI and other GI pathogens, the full incremental benefit of HT can be maintained only if a quick final diagnosis of infectious diarrhea (for both CDI and other GI pathogens) can be achieved. Under the HT testing strategy, 1 test result was needed to confirm the diagnosis of CDI, whereas the LTHT testing strategy required several test results to rule in the diagnosis of CDI. In the HT testing arm, the extended time to wait for test results for other GI pathogens offset any benefits associated with the rapid test. In the standard care arm, in contrast, having to wait longer for a diagnosis of “other GI pathogens” had a minimal impact since standard care took longer to yield results for CDI.

Results should be considered in line with the model limitations, most notably, that the model does not capture the impact that testing strategies might have on survival and only partially and indirectly simulates infection spread within general ward. The chosen approach for estimating new secondary infections does not explicitly simulate the clinical processes occurring to new patients infected with CDI and ignores the risk of infection spread due to other pathogens as well as within cohort bays (i.e., potential transmission resulting from cohorting TP patients together with FP patients) due to a paucity of data.^46^ Due to a paucity of data, we did not account for the increased risk of nosocomial transmission dependent on LOS; presumably, patients experiencing longer LOS within the general ward would be exposed to a higher risk of transmission. Future iterations of the model should attempt to capture these infection spread components. As secondary infections were not fully and explicitly modeled, the potential benefits of rapid testing might have been underestimated. Although DES is not the optimal approach for modeling infection spread,^47^ it is nevertheless able to indirectly estimate secondary infections in an efficient and static way, which is the most common approach taken in past models to capture CDI spread.^48,49^

While evaluating rapid tests for CDI, accounting for the individual and intergenerational health impact of inappropriate antibiotic treatments on antimicrobial resistance results to be challenging.^50^ Due to limited data, the model did not directly capture the increased risk of becoming colonized with *C. difficile* organism upon receipt of inappropriate antibiotic treatment for CDI.^51,52^ Instead, an arbitrary utility decrement was applied to capture the downstream consequences of receiving incorrect antibiotic treatment. Although this parameter had a marginal effect on model results, the model would benefit from better data on the downstream effects of inappropriate antibiotic treatment.

In addition, assuming independence between sequential tests might have underestimated the clinical effectiveness of the comparator, as running sequential tests increases the overall diagnostic accuracy.^53^ Due to a paucity of data on the infection spread of CDI within LTHT strategy, it was also not possible to externally validate the number of secondary cases for CDI associated with the comparator.

Furthermore, the methodological choice of developing a site-specific DES model, as well as the particular nature of the LTHT testing strategy, might also limit the generalizability of the results across providers using different clinical guidelines and testing algorithms. The main drivers of incremental benefit associated with running a POCT ultimately depend on the comparator chosen and its associated limitations. For example, since the LTHT standard care is a highly accurate but slow testing strategy, the key benefit of running a new POCT in this context lies in reducing turnaround time while maintaining high levels of diagnostic accuracy. Were GDH EIA + toxin A/B EIA testing being selected as a comparator, the expected added benefit of a POCT would revolve more around increasing the diagnostic accuracy, as this alternative comparator yields results quickly although with a low diagnostic accuracy. In addition to this, the current analysis compared an HT as a full replacement of standard care at LTHT; future model iterations, however, might assess the new POCT run as part of the current standard care. Future analyses should also attempt to capture the costs and effects (e.g., productivity gains) associated with a rapid test for CDI on the whole of society. Nevertheless, we believe that this study improves on past models for CDI diagnostics in certain aspects.^49,54^ For example, we simulated key processes underlying the care pathway that past models overlooked while also accounting for capacity constraints.

Finally, sampling uncertainty was not captured within the model as a probabilistic sensitivity analysis (PSA) was not conducted. While running a PSA is good modeling practice (40), conducting a PSA for each diagnostic accuracy pair and test turnaround-time values explored as part of the analysis would have significantly increased the model running time. This pragmatic consideration was particularly relevant in the context of the complex resource-constrained DES model being presented. Instead, it was decided to focus on how to translate the modeling results into TPP performance specifications while also identifying what factors might affect the cost-effectiveness of HT. In addition, the biggest drivers of changes in cost-effectiveness were the performance specifications for the hypothetical rapid test for which no data are available, thereby making it difficult to apply any meaningful distributions around such unknown parameters. The model instead attempted to minimize the impact of first-order uncertainty on the outcomes of interest, alongside extensive sensitivity analyses. Nevertheless, further iterations of the model could include a PSA.

### MPS Framework: Key Considerations

Using established methods of early economic modeling, a novel framework was developed to identify the minimum test specifications for key performance dimensions, and the associated maximum costs, based on cost-effectiveness outputs. Due to the uncertainty surrounding the estimated minimum performance requirements, however, it is recommended to test their robustness before presenting the results to stakeholders in the context of TPP development. We therefore conducted extensive sensitivity analyses to test the impact of different parameters and scenarios on the model outputs. This helped to 1) pragmatically identify the main drivers of cost-effectiveness, 2) narratively draw inferences on the impact of each variable on the performance requirements, and 3) suggest areas for future research.

The MPS framework represents one of the first applications of early economic modeling as a means of deriving the minimum performance specifications as set out in TPPs. A NICE-commissioned early economic model estimated if a hypothetical COVID-19 POCT meeting predefined performance benchmarks as presented in the UK MHRA TPP would be cost-effective.^55^ The analysis presented herein, meanwhile, aimed to derive de novo minimum performance specifications based on cost-effectiveness considerations, via a series of interlinked sensitivity analyses, to support the development of a future TPP.

Because of the inevitable amount of uncertainty within an early economic model, initial discussions with stakeholders should take place around 1) what key test performance dimensions to capture within the model, 2) what is the meaning of “minimum” and “optimal” test requirements, 3) what model outputs should be considered to derive minimum performance requirements, and 4) whether it is appropriate to exclude clinical inferior strategies that may be considered cost-effective due to cost-savings.

Core to the MPS framework is the use of WTP threshold per QALY to derive the minimum performance requirements based on cost-effectiveness considerations. The MPS framework is expected to provide informative findings in the context of conditions where there is evidence (or a justification, in the context of a paucity of data) that the test-informed treatment decisions will have an impact on patients’ survival and/or quality of life. In the context of limited evidence (and/or justification) of this, alternative clinical outcomes (e.g. availability of free single rooms) could be measured to quantify the full impact of a new test, beyond the QALY. A possible approach could be conducting a WTP elicitation exercise with the stakeholders involved in the TPP development process.

The increased interest in TPPs for tests heightens the need for guidance as to how to integrate EEE methods in the typical TPP development process. To inform such guidance, more case studies are needed. To efficiently integrate EEE into TPPs into future case studies, it might be appropriate to consider updating existing decision models or building simple and flexible de novo models to allow running extensive sensitivity analyses without requiring substantial computational time. For example, decision trees are used to identify minimum specifications and headroom costs for hypothetical tests based on cost-effectiveness considerations.

We believe that for the purposes of informing TPP development, the model represents a significant improvement from standard practice of eliciting performance specifications solely from experts. We hope that the proposed methods will be considered of use for future applications of EEE in the context of TPP development as a framework to build on.

## Conclusion

A de novo pragmatic approach was developed to identify the minimum performance requirements and maximum costs for new tests, based on cost-effectiveness considerations using the WTP threshold as a decision criterion, while also isolating the most influential parameters.

## Supplemental Material

sj-docx-1-mpp-10.1177_23814683241293739 – Supplemental material for Early Economic Modeling to Inform a Target Product Profile: A Case Study of a Novel Rapid Test for Clostridioides difficile Infection Supplemental material, sj-docx-1-mpp-10.1177_23814683241293739 for Early Economic Modeling to Inform a Target Product Profile: A Case Study of a Novel Rapid Test for Clostridioides difficile Infection by Paola Cocco, Alison Florence Smith, Kerrie Ann Davies, Christopher Michael Rooney, Robert Michael West and Bethany Shinkins in MDM Policy & Practice

sj-docx-2-mpp-10.1177_23814683241293739 – Supplemental material for Early Economic Modeling to Inform a Target Product Profile: A Case Study of a Novel Rapid Test for Clostridioides difficile Infection Supplemental material, sj-docx-2-mpp-10.1177_23814683241293739 for Early Economic Modeling to Inform a Target Product Profile: A Case Study of a Novel Rapid Test for Clostridioides difficile Infection by Paola Cocco, Alison Florence Smith, Kerrie Ann Davies, Christopher Michael Rooney, Robert Michael West and Bethany Shinkins in MDM Policy & Practice

sj-docx-3-mpp-10.1177_23814683241293739 – Supplemental material for Early Economic Modeling to Inform a Target Product Profile: A Case Study of a Novel Rapid Test for Clostridioides difficile Infection Supplemental material, sj-docx-3-mpp-10.1177_23814683241293739 for Early Economic Modeling to Inform a Target Product Profile: A Case Study of a Novel Rapid Test for Clostridioides difficile Infection by Paola Cocco, Alison Florence Smith, Kerrie Ann Davies, Christopher Michael Rooney, Robert Michael West and Bethany Shinkins in MDM Policy & Practice

## Notes

i. At LTHT, patients with watery stools or unexplained diarrhea, defined as stool consistency equal to type 5 to 7 on the Bristol Stool Scale (i.e., a medical tool aid used to visually classify feces into 7 groups depending on stool consistency, ranging from constipation [type 1] to diarrhea [type 7]), are simultaneously tested for CDI and other gastrointestinal pathogens.

ii. First-order uncertainty reflects “the fact that individuals facing the same probabilities and outcomes will experience the effects of a disease or intervention” (40). For example, patients entering the model are assigned a probability of having a CDI that has a fixed mean value attributed to it. At the arrival of each new patient, the DES modeling technique samples a random number from a uniform distribution, compares that random number to the assigned mean probability, and uses this to determine whether a certain individual in the model has CDI. Although the overall likelihood that a patient has CDI is set of a fixed mean, there is a first-order uncertainty introduced at the individual patient level. To reduce the impact of this uncertainty within constrained-DES models, sufficient model iterations need to be run, using different random number draws for each run of the model.

iii. This analysis was restricted to the 15-min turnaround-time value since 1) the latter represents the best-case scenario that test developers should aspire to and 2) it would not be feasible to run this analysis for every possible turnaround time value.

iv. Compared with UK Teaching Hospitals, UK District Hospitals typically have 1) fewer single rooms, 2) fewer patients being tested for CDI, and 3) an off-site laboratory. See Supplementary Table 3.2 for further details.
