# Supplementary material for: Early Economic Modeling to Inform a Target Product Profile: A Case Study of a Novel Rapid Test for Clostridioides difficile Infection
Source: MDM Policy Pract. 2024 Nov 22;9(2):23814683241293739. doi: 10.1177/23814683241293739 (PMC11585019; doi:10.1177/23814683241293739)
Supplement: sj-docx-1-mpp-10.1177_23814683241293739 – Supplemental material for Early Economic Modeling to Inform a Target Product Profile: A Case Study of a Novel Rapid Test for Clostridioides difficile Infection [file sj-docx-1-mpp-10.1177_23814683241293739.docx]

**Appendix 1**

This Appendix includes information on (i) model structure (section 1.1); (ii) clinical assumptions underpinning the model (section 1.1.8); (iii) parameterisation (section 1.2); (iv) model outputs (section 1.3); (v) model implementation (section 1.4); (vi) model validation (section 1.5).

[**Appendix 1 41**](#_Toc155960730)

[**1.1.** **Model structure** 42](#_Toc155960731)

[**1.1.1.** **Clinical assumptions** 44](#_Toc155960732)

[**1.1.2.** **Starting point** 45](#_Toc155960733)

[**1.1.3.** **Escalation of infection-control measures** 45](#_Toc155960734)

[**1.1.4.** **Testing pathway** 46](#_Toc155960735)

[**1.1.5.** **Clinical decision-making upon receipt of test results** 52](#_Toc155960736)

[**1.1.6.** **Antimicrobial treatment for CDI** 53](#_Toc155960737)

[**1.1.7.** **Dynamic bed allocation** 55](#_Toc155960738)

[**1.1.8.** **Estimation of new secondary cases of CDI** 57](#_Toc155960739)

[**1.2.** **Model parameterisation** 58](#_Toc155960740)

[**1.2.1.** **COMBACTE-CDI study datasets** 58](#_Toc155960741)

[**1.2.2.** **Patient characteristics** 61](#_Toc155960742)

[**1.2.3.** **Time-to-event variables: duration of symptoms and length of stay** 62](#_Toc155960743)

[**1.2.4.** **Hospital configuration** 63](#_Toc155960744)

[**1.2.5.** **Inter-arrival time** 64](#_Toc155960745)

[**1.2.6.** **Features of the LTHT testing pathway for patients suspected with CDI** 67](#_Toc155960746)

[**1.2.7.** **Multiplex GI testing panel** 67](#_Toc155960747)

[**1.2.8.** **New secondary cases of CDI** 68](#_Toc155960748)

[**1.2.9.** **Health-related utility weights** 68](#_Toc155960749)

[**1.2.10.** **Costing estimates** 68](#_Toc155960750)

[**1.3.** **Model outputs** 70](#_Toc155960751)

[**1.4.** **Model implementation** 71](#_Toc155960752)

[**1.4.1.** **Model initialisation** 71](#_Toc155960753)

[**1.4.2.** **Handling uncertainty** 73](#_Toc155960754)

[**1.5.** **Model validation** 74](#_Toc155960755)

[**1.6. References Appendix 1** 76](#_Toc155960756)

1. **Model structure**

Consultations with clinical experts based at the Leeds Teaching Hospitals Trusts (LTHT) and a review of local clinical guidelines informed the model structure. Specifically, information on key processes underlying the LTHT laboratory workflow for diagnosing CDI and clinical management for patients suspected with CDI were derived from consultations with an LTHT Principal Clinical Scientist (Dr Kerrie Davies) and a Clinical Research Fellow (Dr Christopher Rooney). Consultations were conducted throughout the development of the structure and parameterisation of the model via online one-to-one meetings between beginning of 2020 and end of 2021.

Discrete event simulation (DES) was selected as the technique of choice for the model because of its key advantages in this case, including: (i) the ability to capture individual patient characteristics (e.g. age and duration of symptoms) and history in the model; (ii) the ability to accurately measure the timings of events, and their associated costs and outcomes (iii) the ability to capture a sequence of hospital processes and activities patients suspected with *Clostridioides* difficile infection (CDI) undergo within the care pathway; and (iv) the ability to capture capacity constraints for scarce resources (e.g. single rooms) and queues in the system.

Based on early clinical consultations, it was considered important that the model be able to capture key elements such as test turnaround time, and availability of hospital rooms. As such, the decision was made to focus the evaluation on a single centre, to reflect current clinical pathways in place at LTHT.

See **Supplementary Figure 1. 1** for a simplified schematic of the model structure.

**Supplementary Figure 1. 1 Simplified schematic of the model**


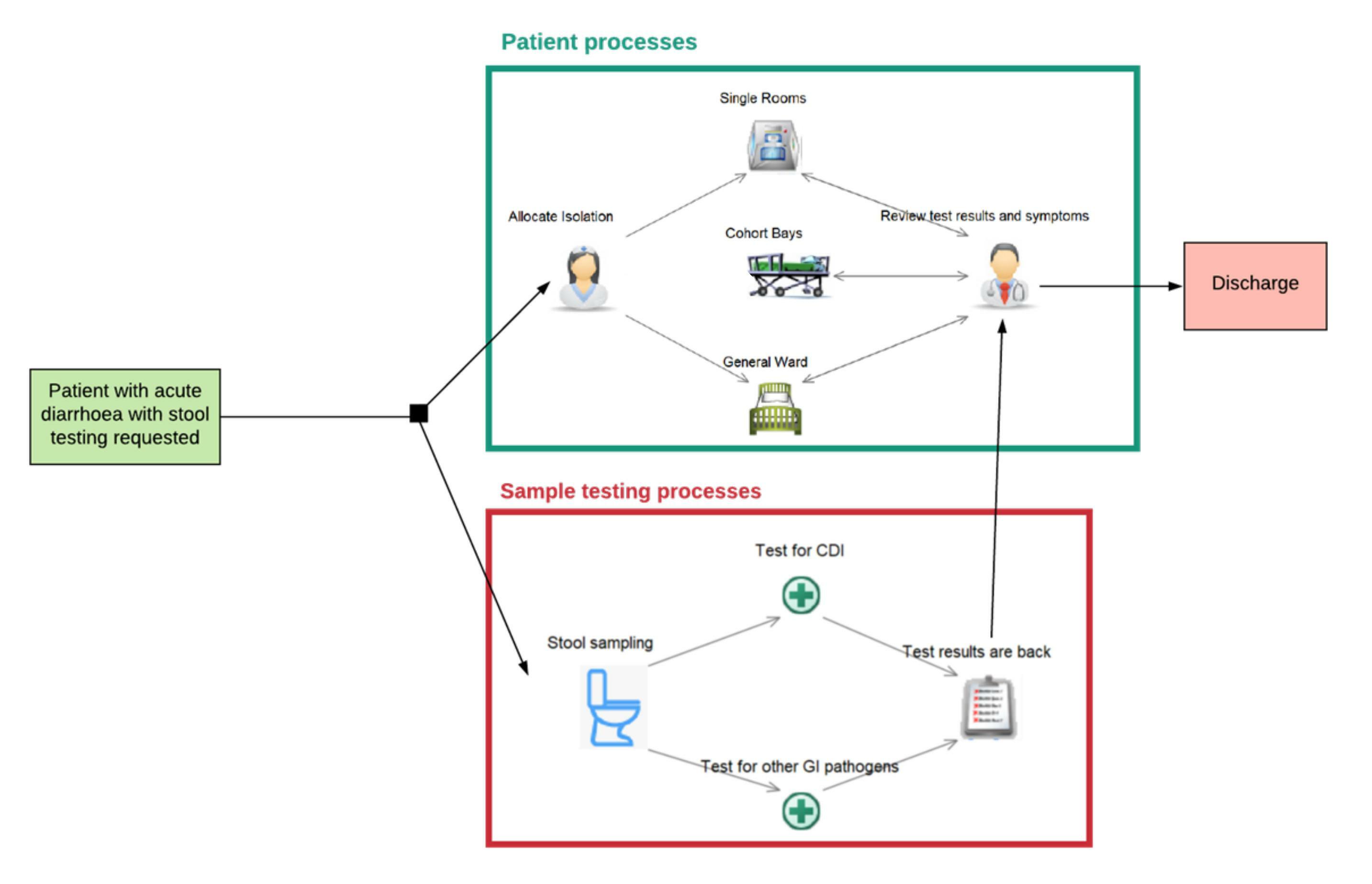


1. **Clinical assumptions**

Key clinical assumptions (CA) underpinning the model are summarised in **Supplementary Table 1. 1**. These assumptions were informed by a series of consultations with clinical experts (including a Principal Clinical Scientist and a Clinical Research Fellow at LTHT).

**Supplementary Table 1. 1 Key clinical assumptions of the model**

| **Clinical Assumptions** |
| --- |
| **1.** The monthly demand for stool testing was assumed to be constant and independent from seasonal change or other external factors. |
| **2.** There is no risk of co-infection between GI pathogens and CDI. |
| **3.** Isolation in single rooms (or cohort bays) is required for patients positive to other GI pathogens until 2 days after symptoms resolution (i.e. resolution of diarrhoea). |
| **4.** When single rooms and cohort bays are at full capacity, patients confirmed with infectious diarrhoea remain in general ward until hospital discharge. |
| **5.** Upon confirmation of infectious diarrhoea, patients in the general ward can be transferred into one of the four cohort bays depending on current availability. Patients confirmed with CDI are grouped separately from patients positive to other GI pathogens, to reduce the risk of developing co-infections. Confirmed patients remain in cohort bays until hospital discharge. |
| **6**. A rapid multiplex GI panel with assumed perfected diagnostic accuracy is run to detect multiple pathogens – separate to tests for CDI.  Perfected diagnostic accuracy was assumed for the multiplex GI panel as the focus of this decision model lies in evaluating testing strategies for CDI, rather than other GI pathogens. |
| **7.** Clinicians consider the continuation or resolution of diarrhoea as the only symptom for CDI – without assessing whether the frequency of stools is improving or worsening. |
| **8.** Clinicians are assumed to pre-emptively administer treatment for CDI (i.e. ‘empirical treatment’) prior to receipt of test results if a patient presents with severe symptoms of CDI – as per current clinical practice at LTHT. |
| **9.** Clinicians fully adhere to test results when deciding when to start or stop administering antibiotic treatment for CDI. |
| **10.** Early treatment, via early diagnosis, is assumed to have no impact on patient survival, risk of disease recurrence and long-term quality of life due to paucity of data. |
| **11.** Patients with a true-positive (TP) result for CDI are assumed to recover to full health without risk of disease recurrence at the end of the antibiotic treatment regimen. |
| **12.** Patients with a false-negative (FN) result for CDI do not experience further disease recurrences. |
| **13.** Patients with a FN results for CDI are assumed to miss antibiotic prescriptions for CDI and this, in turn, increases the risk of health-related quality of life loss due to missed treatment. In addition, FN patients are assumed to be released from single room isolation into general ward upon receipt of an (incorrect) negative result which, in turn, increases the risk of nosocomial transmission of CDI within the general ward. |
| **14.** Patients with a false-positive (FP) result for CDI are assumed to remain symptomatic at day 10 of treatment and to receive treatment for 4 additional days – as per current clinical practice at LTHT. This, in turn, reduces the availability of free single rooms for suspected or confirmed infectious patients thus increasing the likelihood of infection spread in the general ward.  Since patients with a FP test result receive unnecessary antibiotic treatment for CDI they are assumed to remain symptomatic at day 10 of treatment as they are not treated for the real cause of diarrhoea (e.g. non-infectious condition – as per expert opinion). This increases the loss in health-related quality of life due to receiving incorrect antibiotic treatment |
| **15.** An indirect approach is taken to capture the impact of possible CDI infection spread within general ward only, based on calculating the number of new secondary cases using the reproductive rate of infection spread (i.e. an estimate of the number of secondary infected cases resulting from one primary infected patients entering the general ward). The number of primary infected cases in the general ward comprises (i) FN cases incorrectly placed into general ward and (ii) TP cases placed into general ward due to paucity of free single rooms and cohort bays. |
| *CDI – Clostridioides difficile infection; FN – false-negative; FP – false-positive; GI – gastrointestinal infection; TN – true-negative; TP – true-positive* |

1. **Starting point**

In-hospital adult patients with an initial episode of acute diarrhoea for whom clinicians have requested stool testing for CDI enter the model. It was decided to model specifically adult patients given the difference in presentation of symptoms, severity of disease and response to treatments between paediatric and adult populations ^2^.

Patients are moved into presumptive isolation, while their stool samples are tested simultaneously for CDI and other gastrointestinal (GI) pathogens which could cause diarrhoea. Stool testing and presumptive isolation happen simultaneously. The simulation software has the functionality to divide a single *entity* (i.e. patient) into two parts (called *batching* in SIMUL8) which share the same individual-level information (e.g. disease prevalence, time to enter the model), and to re-combine those two parts (called *components*) of an entity at a later event (i.e. once test results are back). This enables the user to simulate different events happening simultaneously to a single entity. This approach is used in the model to track: (i) which single isolation room a patient enters, depending on the current availability of single rooms, and (ii) the various processes of the testing pathway that each individual’s test sample undergoes (e.g. sample preparation, setting the machine, reviewing test results). The division of entities into their two respective components is undertaken at the start of the model. Once the patient’s test result is received, the two components are recombined and information on an individual patient’s health is updated.

1. **Escalation of infection-control measures**

LTHT clinical guidelines recommend placing a patient into presumptive isolation within two hours of suspicion of infective diarrhoea to reduce the risk of nosocomial transmission within the general ward ^3, 4^. Two locations are available for patients suspected with infectious diarrhoea depending on current capacity constraints:

- **single rooms**: only one patient can enter a single room with no potential of infecting others. If there is a confirmation of CDI, the patient remains in isolation until the end of their hospital LOS; and
- **general ward**: when no single rooms are available, patients remain in the general ward while waiting for test result with a higher potential for infection transmission. The general ward is set to an unlimited capacity in the model, to host as many patients as possible, where necessary.

Cohort bays are available for patients confirmed with infectious diarrhoea only – in the absence of available single rooms. At LTHT, patients with the same detected infection can be grouped together in four cohort bays (each with a maximum capacity of 6 patients).

1. **Testing pathway**

Upon sample collection, the sample is shipped to the laboratory or tested within the ward depending on the testing strategy under evaluation. The following features are common to both testing strategies being evaluated: (i) test turnaround time represents how long it takes to yield test results after having obtained the sample to test; (ii) the sample is simultaneously tested for CDI and other GI pathogens to rule out other causes of infectious diarrhoea using a rapid multiplex GI panel with perfected diagnostic accuracy; and (iii) upon receipt of final diagnosis, the test sample is matched to the corresponding patient within the patient isolation pathway, to update information on patient’s health following test results.

**Standard care testing for CDI**

Details of the LTHT laboratory clinical pathway for CDI, outlined below, were derived from consultations with a Principal Clinical Scientist based at LTHT. At LTHT an on-site laboratory routinely processes stool samples at 10am and 4pm. Samples arriving before these time points will wait before being processed.

A two-step testing algorithm is currently run at LTHT for patients suspected with CDI (**Supplementary Figure 1. 2**). Each sample is initially screened with TECHLAB ® C.DIFF CHECK^TM^-60 glutamate dehydrogenase (GDH) enzyme immunoassay (EIA) to detect the presence of *C. difficile* organism. If a sample is negative to GDH EIA, it is possible to exclude CDI.

If a sample is positive on GDH testing, a Cepheid ® Xpert *C. difficile* polymerase chain reaction (PCR) and cell-cytotoxicity neutralisation assay (CCNA) are used simultaneously to detect toxin genes related to CDI or free toxins in stools, respectively. PCR testing yields results quickly (e.g. 43 minutes), whilst CCNA ultimately confirms the presence of free toxins within two days. Final diagnosis is confirmed upon receipt of CCNA results. **Supplementary Table 1. 2** gives an overview of the possible diagnosis, interpretation of test results and clinical recommendation based on both PCR and CCNA test results.

**Supplementary Figure 1. 2 Two-step testing algorithm run at LTHT to diagnose patients suspected with CDI**


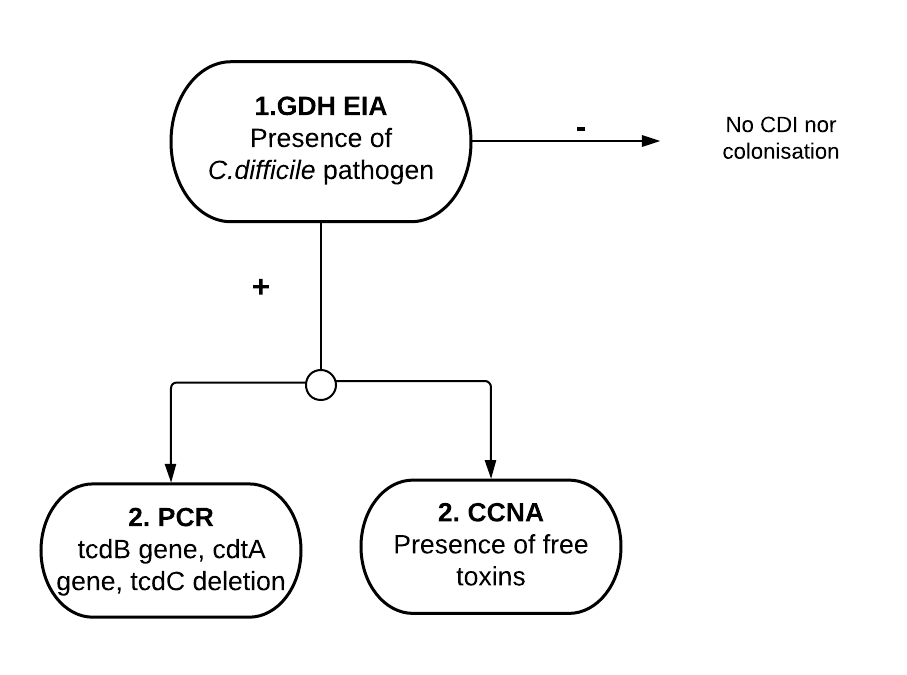


**Supplementary Table 1. 2 Possible diagnoses for samples positive for GDH then tested with PCR and CCNA, outlining interpretation and clinical recommendations according to LTHT clinical guidelines ^4, 5^**

|  | **Combined result** | **Interpretation** | **Clinical recommendation** |
| --- | --- | --- | --- |
| PCR +ve/ CCNA +ve | - Toxigenic strain - Free toxins detected | CDI positive | Contact and isolation measures are continued for the duration of inpatient stay |
| PCR -ve/ CCNA -ve | - Non-toxigenic strain - No free toxins detected | CDI negative | Pending results on other GI pathogens, contact measures and isolation measures can be discontinued. If patient test positive to other GI pathogens, contact and isolation measures are continued until after 2 days from resolution of diarrhoea. If patient test negative to other GI pathogens, contact and isolation measures can be discontinued. |
| PCR -ve/ CCNA +ve | - Free toxins detected | CDI positive | Contact and isolation measures are continued for the duration of inpatient stay |
| PCR +ve/ CCNA -ve | - Toxigenic strain - No free toxins detected | Potentially colonised with *C. difficile* pathogen | Contact and isolation measures are continued for the duration of inpatient stay |
| *CDI – Clostridioides difficile infection; FN – false-negative; FP – false-positive; GI – gastrointestinal infection; TN – true-negative; TP – true-positive* | | | |

**Hypothetical test for CDI**

The intervention being evaluated was therefore a ward-based hypothetical POCT test (henceforth ‘HT’) detecting toxins in stools. There are opposing views on which target diagnostic tests for CDI should detect, be it either free toxins in stool, the organism or genes encoding-toxins ^6^. Exploring the downstream benefits of detecting alternative targets, however, is outside the scope of this early economic model. It was therefore decided to model HT as a toxin detecting POCT since toxin detection has been reported to better correlate with disease severity and patient health outcomes ^7, 8^.

The focus of the model was on capturing the impact of HT on: (i) improving short-term clinical outcomes for patients via an expedited (and appropriate) administration of antibiotic treatment for CDI and (ii) supporting fast and appropriate escalation of infection control measures, to minimise in-hospital transmission and enable rapid de-isolation of non-infected patients. The model simulates two important mechanisms via which the HT may impact on clinical utility and cost-effectiveness outcomes: reduced test turnaround time, and increased diagnostic accuracy (see **Supplementary Figure 1. 3**).

As HT is a hypothetical test, no information is available currently on its diagnostic accuracy, turnaround time or test price. Various scenarios and sensitivity analyses were run in the model varying test turnaround time and diagnostic accuracy.

**Supplementary Figure 1. 3 Simplified schematic of how hypothetical test (HT) affects the hospital system and individual patient health outcomes via reduced test turnaround time and improved diagnostic accuracy**


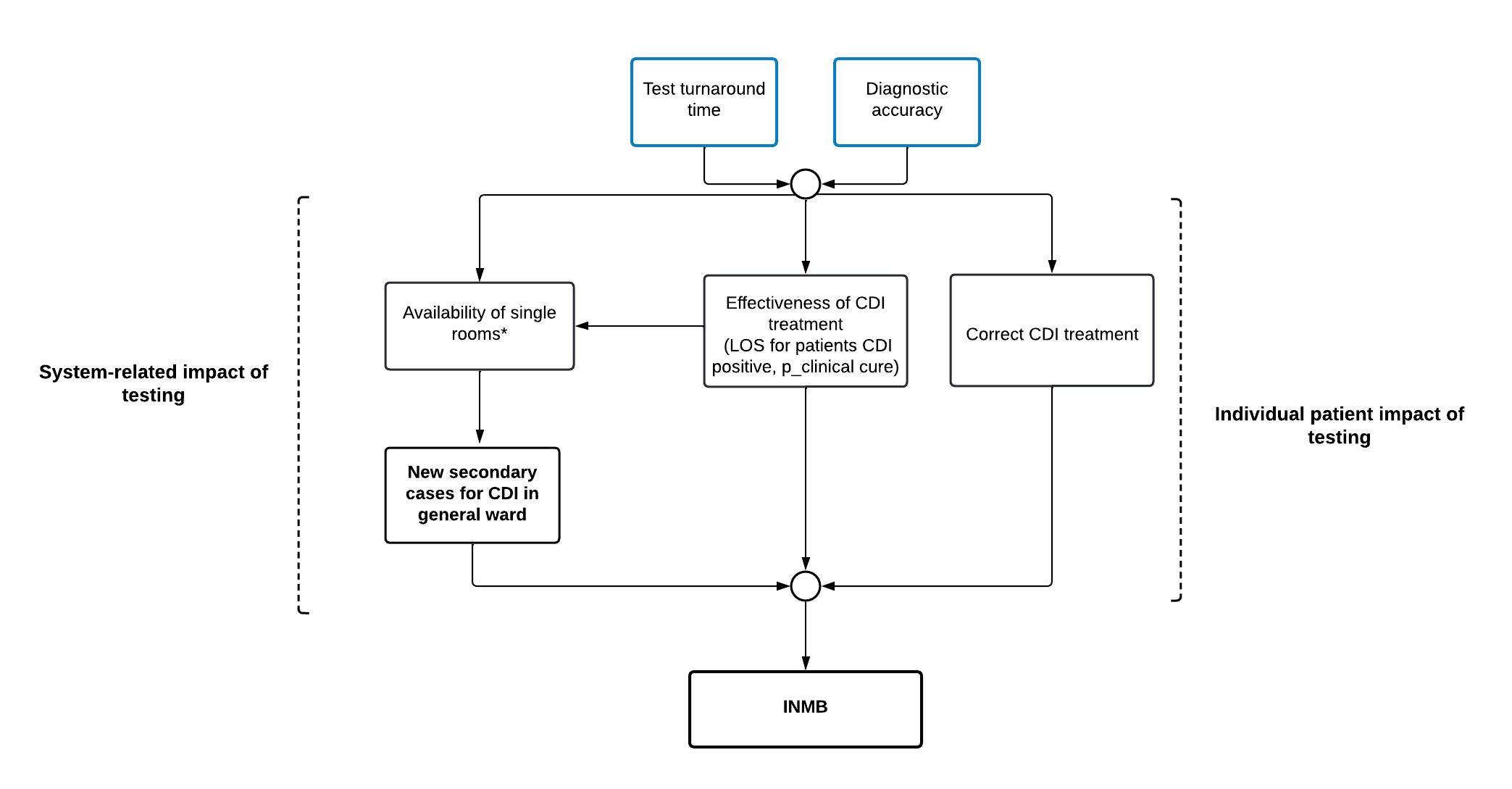


**Early diagnosis**

Upon receipt of positive test result for CDI, patients start antibiotic treatment for CDI – unless treatment has started in advance (i.e. *empirical treatment*) because patients presented with severe symptoms. Receiving test results in a timely manner supports clinicians in administering early treatment for CDI which, in turn, may improve short-term health outcomes for patients.

A HT with reduced turnaround time therefore: (1) increases the probability of clinical cure at day 10 of treatment regimen; as well as (2) reducing the hospital length of stay (LOS) compared to testing strategies with a slower turnaround time ^1^. In the context of early treatment, via early diagnosis, patients who tested positive to CDI experience a worsening in their quality of life for a shorter duration, while also decreasing the number of treatment days required for patients to recover to full health compared to patients who receive confirmation of CDI at later time points. This also leads to cost savings compared to standard care due to reduced total treatment cost and bed cost per patient.

On a system-related level, a HT capable of reducing turnaround time compared to standard care shortens patients’ stay in presumptive isolation whilst awaiting the confirmation of infectious diarrhoea, as well as reducing the LOS patients positive to CDI remain in single room isolation. This results in more single rooms being available for new patients suspected with CDI thus reducing the number of secondary CDI cases in the general ward ^9^.

The possible impact of such a test on improving patient survival and risk of disease recurrence via expediated delivery of antibiotics was not captured in this analysis, due to a current paucity of data in the literature as to the effect of early treatment in this context ^1, 10^. As further data on this emerges, future iterations of this early economic model could be expanded to include this element.

**Increased diagnostic accuracy**

A HT associated with higher diagnostic specificity is able to decrease the number of FP cases (i.e. patients who were given a positive result for CDI, although they did not have the disease), thus reducing the number of unnecessary antibiotic treatments for CDI. This minimises the loss in health-related quality of life due to receiving incorrect antibiotic treatment. In addition, a HT test with improved diagnostic specificity increases the availability of free single rooms for new suspected or confirmed infectious patients. With more single rooms being available, fewer patients are placed in the general ward while waiting for test results, thus minimising infection spread within the general ward. A HT with improved diagnostic sensitivity compared to standard care would reduce the number of false negative (FN) cases and missed antibiotic prescriptions for CDI. On an individual patient level, this minimises the risk of health-related quality of life loss due to missed treatment; on a system-related level, a HT with improved diagnostic sensitivity reduces the risk of FN patients being released into the general ward, which decreases the risk of nosocomial transmission of CDI.

1. **Clinical decision-making upon receipt of test results**

Upon receipt of a patient’s test results, clinicians can decide to: (i) continue isolation; (ii) de-escalate isolation measures and move the patient into the general ward; or (iii) discharge the patient if the patient has spent their assigned LOS in isolation^[[1]](#footnote-1)^. Based on current LTHT clinical guidelines, **Supplementary Table 1. 3** gives an overview of recommended clinical actions for patients suspected with CDI considering both clinical symptoms and test results as simulated within the model.

**Supplementary Table 1. 3 Clinical decision-making for patients suspected with CDI considering symptoms and test results**

|  | **Test results** |
| --- | --- |
| *Symptoms persist* | - **CDI +ve** – patient remains in isolation and antimicrobial treatment for CDI, unless empirical treatment was already started. - **CDI -ve / GI panel +ve** – patient remains in isolation. Set daily checks to assess resolution of symptoms. - **CDI -ve / GI panel -ve** – patient is confirmed with non-infective diarrhoea, isolation is not required. The patient is therefore de-isolated and moved into the general ward for their remaining hospital LOS. |
| *Symptoms disappear* | - **CDI +ve** – patient remains in isolation for the remaining length of hospital stay. Complete CDI treatment regimen. - **CDI -ve / GI panel +ve** – patient remains in isolation for 2 extra days, and subsequently moves into the general ward – unless the patient is de-isolated in advance if case single rooms are in full capacity. - **CDI -ve / GI panel -ve** – patient is de-isolated and moved into the general ward for their remaining hospital LOS. |
| *CDI – Clostridioides difficile infection; GI – gastrointestinal infection; LOS – length of stay* | |

1. **Antimicrobial treatment for CDI**

If a patient presents with severe symptoms of CDI (e.g. evidence of severe colitis, high temperature), clinicians are assumed to start empirical antibiotic treatment before placing the patient into presumptive isolation and prior to receipt of test results ^3^. For patients with mild or moderate symptoms (e.g. approximately 3-5 stools per day), antibiotic treatment for CDI (i.e. vancomycin) starts upon receipt of positive test results for CDI.

The antibiotic treatment regimen for CDI lasts up to 10 days. At day 10, clinicians check if the patient is symptomatic and, if so, treatment is continued for 4 additional days. Once the patient is no longer symptomatic at day 10, treatment for CDI is discontinued and patients are assumed to recover to full health without risk of disease recurrence. Patients with a FP test result receiving unnecessary antibiotic treatment for CDI are assumed to remain symptomatic at day 10 of treatment, as they are not treated for the real cause of diarrhoea (e.g. non-infectious condition – as per expert opinion. Since patients with a FP test result for CDI remain symptomatic, they are assumed to receive treatment for 4 additional days (as per current clinical practice at LTHT).

**Supplementary Figure 1. 4** shows a timeline of key events patients experience within the clinical management for CDI as simulated within the model.

**Supplementary Figure 1. 4 Key events patients experience within the clinical pathway for CDI as simulated within the model**


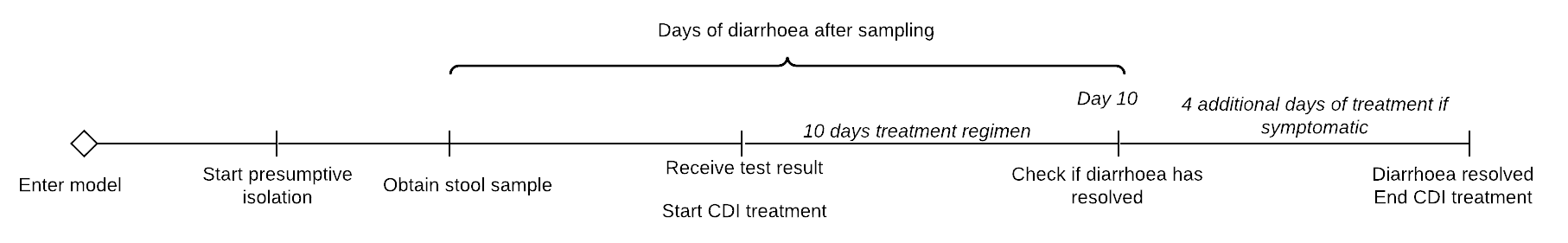


1. **Dynamic bed allocation**

The model has the capacity to resemble as much as possible the dynamic bed allocation processes underlying clinical practice, whilst capturing capacity constraints. The following subsections describe key events happening to patients placed either into single rooms, the general ward or cohort bays upon receipt of final diagnosis. The final section discusses some limitations of the approach chosen for the dynamic bed allocation within the model.

Single room isolation

If single rooms reach full capacity, clinicians can decide to de-escalate infection-control measures for patients who are no longer infectious. Based on expert opinion, patients who are positive with other GI pathogens but asymptomatic can be assigned a lower priority from an infection-control perspective. Once single rooms are occupied, the model has the functionality to de-isolate any asymptomatic patient positive to other GI pathogens in order to release additional single rooms. This patient will then enter the general ward or be discharged depending on their remaining hospital LOS.

Stay in general ward

If a patient within the general ward is confirmed with infectious diarrhoea, they may be transferred into a single room or cohort bay (depending on availability) to reduce risk of transmission. Alternatively, when single rooms and cohort bays are at full capacity, confirmed cases remain in the general ward until hospital discharge. The simulation then counts the total number of infectious CDI patients (i.e. true positive [TP] and FN) within general ward over the model evaluation period (for more information see section 1.1.7).

If a patient within the general ward is reported as not infectious, they stay in the general ward until hospital discharge depending on their simulated LOS, without receiving antibiotic treatment for CDI.

Cohort bay isolation

Upon confirmation of infectious diarrhoea, patients in the general ward can be transferred into one of the four cohort bays depending on current availability. Patients confirmed with CDI are grouped separately from patients positive to other GI pathogens, to reduce the risk of developing co-infections. Confirmed patients remain in cohort bays until hospital discharge.

Limitations of the proposed approach

In the context of single rooms and cohort bays at full capacity, confirmed cases were assumed to remain in the general ward until hospital discharge (CA 4). In the model, the simplifying assumption was adopted that patients who were placed in single room isolation could not be released from single room isolation when other patients with a higher priority needed to be isolated. In clinical practice, however, decisions around when to de-escalate infection control measures are based on a continuous assessment of the symptoms duration of patients being isolated, the risk of infection spread and up-to-date availability of free single rooms – for example, if no single rooms are available, lower priority patients (i.e. asymptomatic patients confirmed with CDI) would be released from single room isolation to accommodate for higher-priority patients. Simulating the continuous bed allocation decisions, and every possible factor having an impact on them, however, would have significantly increased the complexity of model structure, and therefore extended the model running time. The time-to-next event progression and individual feature of DES modelling technique make the task of continuously moving patients confirmed with CDI between general ward and single rooms based on the current availability of free single rooms computationally intensive.

1. **Estimation of new secondary cases of CDI**

DES is not capable of directly tracking infection spread within the modelled system without intense computation. Nevertheless, DES is capable of efficiently approximating the infection spread. As such an approximation of the infection spread was captured in the model using a reproductive rate of infection spread (R_0_) – which is represents an estimate of the number of secondary infected cases resulting from one primary infected patient ^11^.

Similar to existing decision models for CDI diagnostics ^12, 13^, at the end of the model simulation a simplified approach was taken to indirectly estimate the number of secondary infection cases resulting from infected patients entering the general ward, using R_0_ (i.e. as opposed to adopting a SD model):

$$Secondary infection cases=primary infection cases in general ward \times R_{0}$$

The number of primary infection cases in the general ward comprises of patients positive to CDI who were placed in the general ward due to scarcity of single rooms and cohort bays, and those patients incorrectly de-isolated upon receipt of a FN test result.

The model estimated the potential number of secondary infection cases due to patients confirmed with CDI being in the general ward only, as patients positive to CDI within single rooms and cohort bays pose a minimal risk of wide-spread transmission. The rate of infection, however, is assumed to be constant regardless of the number of secondary cases occurring in the model. In addition, the model only counts the number of secondary cases at each run, and estimates the associated healthcare costs and quality-adjusted life year (QALY) lost – without explicitly simulating the events and clinical processes occurring to new patients infected with CDI.

1. **Model parameterisation**

This section describes the assumptions and data used to populate the model**. Supplementary Table 1. 4** provides an overview of model parameters and related sources. Key model parameters relating to patient characteristics and hospital configuration are based on the COMBACTE-CDI study datasets. The following subsections describe the other data sources used to parameterise the model.

1. **COMBACTE-CDI study datasets**

Combatting Bacterial Resistance in Europe (COMBACTE-CDI) was a multicentre European-wide epidemiological study assessing the impact of CDI on patients’ health and clinical practice across 119 healthcare sites from 12 European countries, conducted from 2018 to 2021^[[2]](#footnote-2)^ ^14^. Full details on the COMBACTE-CDI trial are available in previous publications ^14, 15^.

In particular, UK-individual patient data (IPD) from the COMBACTE-CDI case report form (CRF) dataset (n= 180 patients from 23 UK sites) was used to inform model parameters relating to patient characteristics (e.g. disease severity, symptoms duration, hospital LOS). This dataset contains anonymised individual patient data on: (i) demographics; (ii) admission history (e.g. reason for hospital admission, previous treatments received); (iii) laboratory testing (e.g. information on testing for any GI pathogens in advance to CDI testing, CDI diagnosis); (iv) clinical information (e.g. comorbidities, disease severity for CDI, diarrhoea duration); (v) drug history; (vi) CDI treatment escalation; (vii) surgery and GI interventions; and (viii) health outcomes (e.g. hospital discharge, re-admission, death).

Parametric survival analysis was conducted to determine appropriate distributions for time-to-event data in the model (i.e. duration of symptoms for CDI positive and negative patients, and hospital LOS). For each of these variables, different parametric models (Normal, Lognormal, Exponential, Gamma and Weibull) were fitted to the UK-based COMBACTE CDI IPD data using the R ‘fitdistrplus’ package ^16^. Based on a maximum likelihood estimation (MLE) process, the optimal fitting distribution for each variable was chosen based on: (i) an analysis of the Akaike Information Criterion (AIC) and Bayesian Information Criterion (BIC) metrics (the distribution with the lowest AIC and BIC values indicating the parameterisation with the optimal statistical fit to the data); (ii) an analysis of key summary statistics produced from the various parameterisations (i.e. mean, median, inter-quartile range, standard deviation [SD], and maximum values); and (iii) visual inspection of the goodness of fit of the different parametric models against the observed patient data ^16^.

In addition, UK-specific summary data from a European-wide survey part of COMBACTE-CDI study was used to inform key model parameters related to hospital configuration (e.g. numbers of samples run and single rooms available). This survey was sent out to community and hospital sites (n=158) across 12 European countries to assess current clinical practices for CDI patients and CDI costs ^17^.

| **Parameter** | **Estimate (SD)** | **Distribution type** | **Data Source** |
| --- | --- | --- | --- |
| *Disease-related parameters* | | | |
| CDI disease prevalence | 10.342% | Probability profile^[[3]](#footnote-3)^ | ^19^ |
| Other GI pathogens prevalence | 13.801% | Probability profile | ^19^ |
| CDI patients with mild symptoms | 39% | Probability profile | COMBACTE-CDI CRF dataset |
| CDI patients with moderate symptoms | 33% | Probability profile | COMBACTE-CDI CRF dataset |
| CDI patients with severe symptoms | 27% | Probability profile | COMBACTE-CDI CRF dataset |
| Length of stay CDI negative patients (days) | 28.6 (38.51) | Weibull | COMBACTE-CDI CRF dataset |
| Duration diarrhoea after sampling CDI negative patients (days) | 7.041 (11.98) | Weibull | COMBACTE-CDI CRF dataset |
| Duration diarrhoea after sampling CDI positive patients (days) | 12.27 (26.04) | Weibull | COMBACTE-CDI CRF dataset |
| UK-England utility weight general population aged 55-64 | 0.819 | Fixed | ^20^ |
| UK utility weight adult hospitalised patient with first episode of CDI | 0.42 | Fixed | ^21^ |
| Decrement utility weight due to inappropriate antibiotic treatment | 0.10 | Fixed | Assumption |
| Duration CDI disease | 10-14 days | Fixed | ^19, 22^ |
| Duration CDI clinical management | 30 days | Fixed | ^1, 10^ |
| Reproductive ratio for CDI (median) | 1.04 | Fixed | ^23^ |
| Probability of clinical cure – slow diagnosis | 85.3% | Probability profile | ^1^ |
| Probability of clinical cure – average diagnosis | 90.7% | Probability profile | ^1^ |
| Probability of clinical cure – rapid diagnosis | 95.6% | Probability profile | ^1^ |
| Length of stay CDI positive patients – slow diagnosis (mean days) | 30.3 (36.3) | Weibull | ^1^ |
| Length of stay CDI positive patients – average diagnosis (mean days) | 26.9 (28.9) | Weibull | ^1^ |
| Length of stay CDI positive patients – rapid diagnosis (mean days) | 23.2 (25.4) | Weibull | ^1^ |
| *Diagnostic accuracy* | | | |
| GDH EIA sensitivity | 94% | Fixed | ^24^ |
| GDH EIA specificity | 94% | Fixed | ^24^ |
| PCR sensitivity | 95% | Fixed | ^24^ |
| PCR specificity | 98% | Fixed | ^24^ |
| CCNA sensitivity | 86.4% | Fixed | ^25, 26^ |
| CCNA specificity | 99.2% | Fixed | ^25, 26^ |
| Multiplex GI panel testing sensitivity | 100% | Fixed | Assumption |
| Multiplex GI panel testing specificity | 100% | Fixed | Assumption |
| *Testing workflow* | | | |
| Monthly median stool samples tested in UK Teaching Hospitals (n) | 1430.5 (689) | Fixed | COMBACTE-CDI survey |
| Proportion of samples tested for CDI only | 5%-69% | Fixed | COMBACTE-CDI survey |
| Time to obtain stool sample | 0.5 (0-2 day) | Triangular | ^10^ |
| Time to transport sample to the laboratory | 15 min | Fixed | Assumption |
| Techlab C.diff Check GDH EIA operating time | 60 minutes | Fixed | ^27^ |
| Xpert *C. difficile* BT Cepheid PCR operating time | 43 minutes | Fixed | ^28^ |
| CCNA operating time (day) | 1.5 (1-2) | Triangular | Expert opinion |
| Multiplex GI pathogens panel operating time | 43 minutes | Fixed | Equal to PCR |
| Time for preparing sample batch | 30 minutes | Normal | Expert opinion |
| Xpert *C. difficile* BT Cepheid PCR operating time | 1 minute | Fixed | ^29^ |
| Time for preparing CCNA | 20 minutes | Fixed | Expert opinion |
| Time to review positive test results | 30 minutes | Fixed | Expert opinion |
| Time to review negative test results | 0 minutes | Fixed | Expert opinion |
| *Costs* | | | |
| Cost of bed day in adult isolation | £692.83 | Fixed | ^10^ |
| Cost of bed day in general ward | £583 | Fixed | ^10^ |
| GDH EIA cost per kit | £4.84 | Fixed | ^10^ |
| PCR cost per run | £26.90 | Fixed | ^10^ |
| CCNA cost | £3.74 | Fixed | ^30^ |
| Multiplex GI panel cost per sample | £43.03 | Fixed | ^10^ |
| Vancomycin 125mg (PO every 6 hours for 10 days) | £132.49 | Fixed | ^31^ |
| Additional cost per secondary case per day | £957.18 | Fixed | ^32^ |
| *Hospital configuration* | | | |
| Single rooms in typical UK Teaching Hospital (n) | 93 | N/A | COMBACTE-CDI survey |
| *CCNA – cell cytotoxicity neutralisation; CDI – Clostridioides difficile infection; COMBACTE – Combatting Bacterial Resistance in Europe; GDH – glutamate dehydrogenase; GI – gastrointestinal infection; PCR – polymerase chain reaction* | | | |

**Supplementary Table 1. 4 Model parameters, related data sources and distribution type**

1. **Patient characteristics**

Patients entering the model are grouped depending on their infection status for CDI and other GI pathogens. CDI disease prevalence is set at 10.342%, whereas prevalence for other GI pathogens is equal to 13.801%^[[4]](#footnote-4)^ ^19^. Both estimates were based on a clinical effectiveness meta-analysis review conducted in 2015 alongside a UK-based economic model on testing strategies for patients suspected with gastroenteritis ^19^. This meta-analysis review pooled data from two large USA-based studies evaluating the performance of multiplex GI panels (n=2,963) ^33, 34^.

It was assumed that there was no risk of co-infection between CDI and other GI pathogens (CA 2). If a patient is confirmed to have CDI, they were assumed to be negative to other GI pathogens; conversely, if a patient is positive to other GI pathogens, they were assumed to be negative to CDI. Based on UK-specific COMBACTE-CDI CRF dataset, the expected prevalence of co-infection is negligible as only small proportion of patients negative to CDI tested positive to other GI pathogens (n= 12, 9%); similarly, few confirmed CDI patients appeared to be positive to other GI pathogens (n=4, 13%). A similar modelling approach was taken by past models on CDI diagnostics which accounted for testing for other GI pathogens ^10, 19^. Recent USA-based prospective studies have found no significant differences in terms of CDI severity, treatment effectiveness, recurrences and length of hospital stay between patients co-infected and patients confirmed with CDI only ^35, 36^. It is unclear therefore if simulating the risk of co-infection would have changed the clinical management and short-term outcomes for patients confirmed with both CDI and other GI pathogens. This simplifying assumption was therefore adopted as there is currently a lack of agreement as to how to treat patients who tested positive to more than GI pathogens. In addition to this, the focus of this model lied in the diagnosis of patients positive to CDI only, rather than every GI pathogen responsible for infectious diarrhoea.

Patients with positive result for CDI are further grouped depending on their disease severity. European estimates from the COMBACTE-CDI CRF dataset informed the proportions of patients with mild, moderate and severe CDI. European estimates were selected due to a paucity of UK data on disease severity.

1. **Time-to-event variables: duration of symptoms and length of stay**

**Patients negative to CDI**

Duration of diarrhoea and length of hospital stay for truly CDI negative patients (i.e. TN and FP cases) was informed by UK-specific IPD from the COMBACTE-CDI CRF dataset (n=180). Truly CDI negative patients were reported to remain symptomatic on average up to 7.041 days after sampling (11.98 SD) (n=53) and to stay at the hospital around 28.6 days (38.51 SD) (n=68). Based on the parametric survival analysis, a Weibull distribution was selected as the best fitting distribution for the duration of symptoms and LOS for patients negative to CDI parameters.

**Patients positive to CDI**

UK-specific IPD from COMBACTE-CDI CRF dataset also informed the duration of symptoms for truly CDI positive patients (i.e. TP and FN cases), which was estimated at 12.27 days on average (24.04 SD) (n=12). Based on the parametric survival analysis, a Weibull distribution was applied to the duration of symptoms for patients with a positive CDI result parameter.

LOS for truly positive CDI patients was based on a prospective time-series study conducted in a 750-bed hospital in France (n= 126 patients positive to CDI), which evaluated the impact of three testing regimens on the clinical management and short-term outcomes for patients with CDI. This study found that receiving rapid diagnosis for CDI (approximately 15 hours) had a positive impact on patient management while reducing hospital LOS and increasing the probability of being clinically cured at the end of the treatment regimen for CDI ^1^.

Depending on how quickly patients who tested positive to CDI (i.e. TP cases) receive a final diagnosis, a certain hospital LOS and probability of clinical cure at day 10 are assumed with rapid testing strategies leading to better short-term clinical outcomes compared to slower testing options. **Supplementary Figure 1. 5** reports the different probabilities of clinical cure at day 10 of treatment regimen for CDI and hospital LOS depending on the time to receive final diagnosis ^1^. Time cut-off points (e.g. 0.5 day, 1.2 day) reflect the mean time-for-return of results across the different testing strategies as reported in the prospective time-series study ^1^. It was assumed that patients with a FN result for CDI experienced a LOS equal to the LOS patients with a positive CDI result might experience in the context of slow diagnosis. This helped to capture the impact of missed diagnosis on patients’ hospital LOS.

Based on the summary data from the prospective time-series study, different parameterisations were explored for these variables (Normal, Lognormal, Exponential, Gamma and Weibull). The best fitting distribution was selected based on an analysis of key summary statistics (mean, median, interquartile range, standard deviation and maximum value). Based on this analysis, a Weibull distribution was applied to the LOS variables for patients positive to CDI.


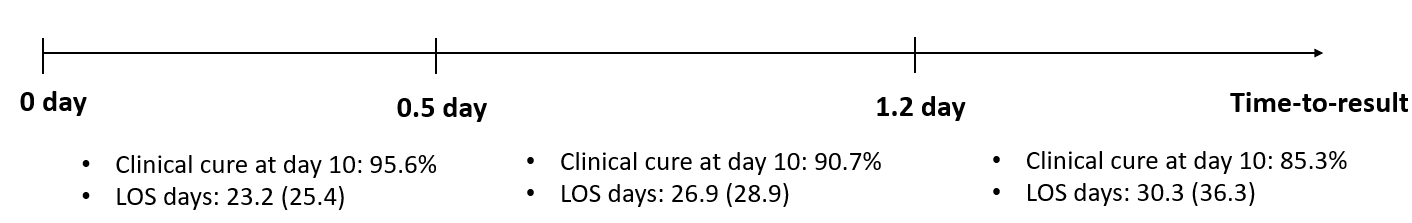


**Supplementary Figure 1. 5 Probabilities of clinical cure at day 10 of treatment regimen for CDI and hospital length of stay (SD), sorted by time-to-final diagnosis. Source: Barbut, Surgers 1.**

1. **Hospital configuration**

UK-specific summary data from the COMBACTE-CDI survey informed the total number of single rooms available (**Supplementary Table 1. 5**). In the UK, 8 Teaching Hospitals took part in this survey which provides the most comprehensive and up to date information on bed capacity and availability of single rooms within the UK. Estimates from this dataset were also validated with clinical experts.

According to the COMBACTE-CDI survey data, UK Teaching Hospitals had an average capacity of 279 side rooms. Not all of these single rooms would be used for patients suffering with GI symptoms however - some would be used for patients with other suspected or confirmed infectious conditions (e.g. acute meningitis, severe acute respiratory syndrome) ^37^. Based on expert opinion, it was assumed that one-third of the reported single rooms in the COMBACTE-CDI survey would be allocated for patients with suspected diarrhoea. Based on this estimate, this model simulated 93 single rooms where patients suspected with infectious diarrhoea could be placed^[[5]](#footnote-5)^.

**Supplementary Table 1. 5 Single rooms availability in UK Teaching Hospitals (n=8). Source: COMBACTE-CDI survey**

|  | **UK Teaching Hospitals** | **Source** |
| --- | --- | --- |
| Mean (n) | 278.7 | COMBACTE-CDI survey |
| Standard Deviation | 93.8 |  |
| Upper bound 95% CI (n) | 365.5 |  |
| Lower bound 95% CI (n) | 192 |  |
| Proportion of single rooms allocated for patients suspected with infectious diarrhoea | 33% | Expert opinion |
| Single rooms simulated (n) | 93 | Estimated |
| *CDI – Clostridiodes difficile infection; COMBACTE-CDI – Combatting Bacterial Resistance in Europe; CI – confidence interval* | | |

1. **Inter-arrival time**

The speed at which new patients enter the model was dictated by an assigned inter-arrival time. For the purpose of this model, the inter-arrival time should equal the expected frequency at which patients develop diarrhoeic symptoms within the hospital setting. This estimate was based on the average number of stool samples tested in the laboratory over a month from the COMBACTE-CDI (see **Supplementary Table 1. 6**). The observations from this data were highly right-skewed, with the mean being greater than the median due to a small number of high outliers in the data (n=2). Based on consultations with a clinical expert, the median number of samples being tested (for any condition) was considered to better represent the workload within a typical UK Teaching Hospital. This estimate (n= 1430) was thus applied in the model.

**Supplementary Table 1. 6 Monthly average stool samples being tested in UK Teaching Hospitals (n=8). Source: COMBACTE CDI survey**

|  | **UK Teaching Hospitals** | **Source** |
| --- | --- | --- |
| Mean | 1,923 | COMBACTE-CDI survey |
| Standard Error | 688.7 |  |
| Median | 1,430.5 |  |
| Standard Deviation | 1,948.1 |  |
| Minimum | 70 |  |
| Maximum | 6,239 |  |
| Upper CI (95%) | 3,551 |  |
| Lower CI (95%) | 294 |  |
| *CDI – Clostridiodes difficile infection; COMBACTE-CDI – Combatting Bacterial Resistance in Europe; CI – confidence interval* | | |

This data, however, included stool samples being tested for other conditions, not only CDI. In addition to the above data, the COMBACTE-CDI survey included a question about the proportion of stool samples being tested only for CDI (see **Supplementary Table 1. 6**). Respondents were asked to select from a multiple-choice question about the proportion of samples being tested for CDI only on a monthly basis (including: ‘less than 30% of samples’, ‘between 30-49%’, ‘between 50-69%’, ‘between 70-99% or ‘100%’ of samples’). Because of the wording of the question, the frequency of the specific values within each range was unknown. As such, within each range, a uniform distribution was applied so that each value within the range was assumed to be equally likely to occur. In addition, for the first range (e.g. 0-30%) a minimum proportion of samples being tested for CDI was set equal to 5% to ensure functionality of the simulation. Out of 8 participating UK sites in the COMBACTE-CDI survey, the majority reported testing between 30%-49% samples for CDI (50%, n=4) or, alternatively, less than 30% of samples (25%, n=2). The remaining UK sites would test between 50%-69% of samples for CDI only (25%, n=2). This information was combined with the median number of samples reported in **Supplementary Table 1. 6** to calculate the number of monthly samples tested for CDI in the model.

The monthly number of stool samples tested for CDI only was approximately equal to 539. As the monthly demand for stool testing was assumed to be constant and independent from seasonality or other external factors, an exponential distribution was selected to calculate the inter-arrival time. In the model, the monthly average of stool samples tested for CDI only equates a mean inter-arrival time of 35 minutes (meaning that a new symptomatic patient enters the model on average every 35 minutes), assuming an exponential distribution.

1. **Features of the LTHT testing pathway for patients suspected with CDI**

Diagnostic accuracies for the two-step LTHT testing algorithm were based on published estimates taking toxigenic culture as the diagnostic reference method ^24-26^. Due to a lack of data on sequential testing, independence between sequential tests was assumed.

Time to prepare a batch of samples to be tested and to set CCNA was based on expert opinion. A Normal distribution was applied to the ‘time for preparing sample batch’ variable to account for ± 25% variation from the mean, whereas a triangular distribution was applied to the operating time for CCNA based on the upper and lower bounds provided by the clinical expert. Manufacturers’ instructions for use ^27-29^ and published literature ^24, 38^ informed the time to set up and processing time for each testing option within the LTHT testing algorithm.

Time to review CDI test results was based on expert opinion. If GDH results are negative, the LIMS sends an automatic alert to the clinician in the ward. Similarly, in case of PCR negative results, the LIMS automatically alerts the clinician. For positive results for CDI (e.g. GDH, PCR, CCNA), a biomedical scientist will review the results within 30 minutes – as per expert opinion.

1. **Multiplex GI testing panel**

The multiplex GI panel, which detects the presence of other GI pathogens in patients with negative results for CDI, was assumed to have perfected diagnostic accuracy. This simplifying assumption was applied as the focus of this model lied in evaluating testing strategies for CDI, rather than other GI pathogens. A similar modelling approach was applied in the single economic model from the rapid literature review of CDI decision models review which evaluated the cost-effectiveness of a multiplex GI panel ^10^.

Based on expert opinion, one sample per time can be processed by the multiplex GI panel on an average time of 43 minutes. Three multiplex GI panels are available at LTHT to test samples for other GI pathogens – as per expert opinion – hence this number of devices were included in the model.

1. **New secondary cases of CDI**

A reproductive rate of infection spread of 1.04 was applied within the model as a baseline value, whereas the minimum (1.99) and maximum (0.52) values of this parameter were varied in the univariate sensitivity analysis conducted as part of the model analysis. This estimate was obtained from an epidemiological model on CDI in-ward transmission, which was based on a retrospective cohort study of an outbreak of CDI in a USA-based tertiary hospital in 2008 ^23^. Existing decision models for CDI diagnostics have all applied this same reproductive rate of infection spread from this epidemiological model ^12, 13, 39^. No equivalent data in the UK context could be identified.

1. **Health-related utility weights**

Health-related utility weights were based on published estimates. Baseline utility weights for UK-England general population were obtained from the European Quality of Life-5 Dimensions (EQ-5D) index population norm data ^20^. Health-related utility weights associated with CDI were based on a prospective patient self-assessment of quality of life study conducted with UK adult inpatients with a first CDI episode (n=30) ^21^.

Upon administration of antibiotic treatment, *C.difficile* and other bacteria have increasingly become resistant to certain antibiotics, causing suboptimal clinical outcomes for patients and treatment failures ^40^. Receiving unnecessary antibiotic treatment for CDI might increase the risk of developing CDI by disrupting the normal bowel flora and allowing for the opportunistic proliferation of the pathogen ^41^. To account for the detrimental effect of receiving unnecessary antibiotic treatment for CDI upon receipt of a FP diagnosis, a 10% reduction in quality of life until discharge compared to TN cases was applied. This estimate was based on expert opinion due to a paucity of data.

1. **Costing estimates**

The modelled costs were based on published data and included testing costs, CDI treatment costs, bed costs and additional costs due to secondary infections. Costs were inflated to 2021 prices using the Bank of England inflator where appropriate ^42^.

*Testing costs*

Testing costs were based on published estimates from the Newcastle Microbiology Laboratory ^10^, inflated to 2021 from 2017 prices. Testing costs included the cost per testing kit and reagents only ^10^. Labour and transportation costs were excluded for the following reasons: (i) transportation costs were assumed to be negligible as the model simulated a UK Teaching Hospital with the availability of an on-site laboratory for testing (hence no transport would be required in this scenario); and (ii) labour costs associated with running a POCT and a laboratory-based testing strategy were assumed to be equivalent. A similar modelling approach was taken by the NICE-commissioned early economic model of COVID-19 POCTs ^43^.

*CDI treatment costs*

Costs for CDI treatment regimens were taken from the British National Formulary (BNF). Patients confirmed with CDI receive oral vancomycin 125mg every 6 hours for 10 days according to the LTHT clinical practice ^3^. Every vancomycin pack contained 28 capsules. Assuming one patient receive 4 capsules per day (24h/6h), one pack with 28 capsules is expected to cover 7 days of treatment per patient. For each patient, the total number of CDI treatment days was divided by 7 to estimate how many vancomycin packs were needed. This estimate was then multiplied by the BNF vancomycin 125mg Drug Tariff price equal to £132.49 ^31^. A similar costing approach was taken by a UK-based decision model on CDI diagnostics ^10^.

*Bed Costs*

Costs for bed-days in the general ward were taken from UK-based decision model of CDI diagnostics ^10^, based on the NHS Reference Costs 2015/2016 ^44^. This estimate was based on the average costs for patients with GI infections without interventions, taken from the elective inpatient spreadsheet. To estimate the cost per adult bed day in the isolation ward, an additional cost was added to the cost for bed days in the general ward to account for single room isolation ^10^.

*Additional costs due to secondary infections*

A UK retrospective cohort study estimated the cost per day for first CDI episode equal to £836 using micro-costing data from 45 hospitalised patients ^32^. This cost per day was multiplied by the typical duration of clinical management for a CDI patient (e.g. 30 days) to calculate the cost of a secondary CDI infection in the model ^10^.

1. **Model outputs**

The clinical effectiveness of each testing strategy was measured in terms of two key outputs: (1) QALY gains; and (2) the number of secondary infections prevented. Since CDI is a transient disease, QALYs were estimated by first calculating the quality-adjusted life days (QALDs) lost due to CDI, and then converting this into QALYs lost ^19, 45^. The individual QALD lost is estimated as follow ^45^:

$${QALD}_{i} lost= \left( u_{n}-u_{CDI} \right)\times t$$

where $i$ is the individual patient, $u_{n}$ is the baseline utility weight for UK general population , $u_{CDI}$ is the health-related utility weight associated with CDI ^21^ , and $t$ is the number of days.

Truly infected patients who test positive for CDI are assumed to experience worsened quality of life for the duration of CDI treatment, and then to recover without risk of disease recurrence. Patients receiving a FN test result, meanwhile, are not provided with the necessary CDI treatment and are assumed to experience lower quality of life for the duration of their whole hospital stay.

Truly healthy patients who test negative for CDI (i.e. true negative [TN] cases) experience no loss in quality of life due to CDI. Patients receiving a FP test result are administered unnecessary antibiotic treatment and are therefore assumed to experience an associated quality of life decrement for the duration of their treatment regimen. Due to a paucity of data, individuals receiving unnecessary CDI treatment in the model were assumed to experience a worsened quality of life compared to TN cases (see section 1.1.5).

To account for the new secondary infections, QALDs lost due to secondary cases were calculated as:

$$QALDs lost due secondary infections=Secondary CDI infections \times\left[ \left( u_{n}-u_{CDI} \right)\times t_{CDI} \right]$$

where $t_{CDI}$ is CDI disease duration which is set to 14 days ^22^. Total QALDs lost comprises: (1) the sum of individual QALDs lost; and (2) the QALDs lost due to secondary infections. Total QALDs lost are then converted into QALYs lost by dividing by 365 days.

Finally, to enable standard cost-effectiveness calculations to be undertaken, the total QALY lost values were converted to QALY gains using an arbitrary common baseline value (in this case, 50) as shown below:

$$QALY gains=50-total QALY lost$$

Cost-effectiveness outputs of the model were expressed in terms of INMB from the UK NHS perspective (i.e. including direct healthcare costs), comparing each HT against standard care, using the NICE WTP lower threshold per QALY of £20,000. Clinical and cost-effectiveness outputs are calculated at the aggregate level – focusing on the total number of patients part of the *evaluation set* (i.e. those entering within the model entry period). The time horizon for the analysis is 7 months (see section 1.4.1 for further detail) and therefore a discount rate of 0% was applied.

1. **Model implementation**

The model was implemented in the commercial software SIMUL8 version 28.0.0.4060 Student edition, and run on a VivoBook ASUS laptop X571GT_F571GT with a 2.60 GDHz Intel ® Core ™ i7 processor and 16 GB of memory under Microsoft Windows 10 Home (build 19,043). Total model run time was 20 minutes for HT testing strategy and 40 minutes for LTHT testing strategy. The model runs from 9am to 7pm (10 hours = 600 minutes in SIMUL8) for 5 days each week (Monday to Friday). Simulated time within the model progresses according to the *next time-to-event progression* mechanism.

The starting conditions of the model are first outlined, followed by an overview of how the uncertainty was handled in the model.

1. **Model initialisation**

A *warm-up period* of 45 days (i.e. nine weeks) was applied in the model, to appropriately capture ongoing capacity constraints. Starting with the model ‘cold’ (i.e. all single rooms available and no patients in the system) would overestimate the hospital’s capacity to test and isolate patients; simulating a warm-up period therefore helps to appropriately capture the steady-state capacity constraints patients face upon entering a busy hospital. In addition, all analyses were based on running 70 *model replications* (i.e. running the model 70 times, with each run using a different random number sequence). This approach ensures that the impact of *first-order* uncertainty on the model results is appropriately accounted for.

The warm-up period and replication number were set to values sufficient to provide stable outputs for the number of secondary infections within the general ward. As per good modelling practices for DES models ^46^, the warm-up period and model replication number were set to ensure a less than 1% difference observed between output values across increasing model replications.

Upon completion of the warm-up period, each model replication runs for a total of 7 months (i.e. the *results collection period*). The model records outputs for every patient that enters the hospital during the *model entry period*, which is set equal to 60-days (two months) following the end of the warm-up period. Extending the results collection period to 7 months ensures that every patient within the model *evaluation set* (i.e. those entering within the model entry period) can have their full experience of the clinical pathway simulated. Whilst clinical management of CDI patients within the hospital usually lasts approximately up to one month ^1, 10^, a proportion of patients experience extended hospital stays (as reflected in the right-skewed tails of the LOS distributions applied in the model). A seven-month extended results collection period was therefore required to ensure outputs for patients entering during the model entry period were captured. Whilst additional patients were allowed to enter the model after the model entry period (to continue to simulate the busy hospital environment), only results for those patients who entered the model during the model entry period were recorded. **Supplementary Figure 1. 6** provides a schematic of the timeline for the model analysis.

**Supplementary Figure 1. 6 Relevant time points for model analysis**


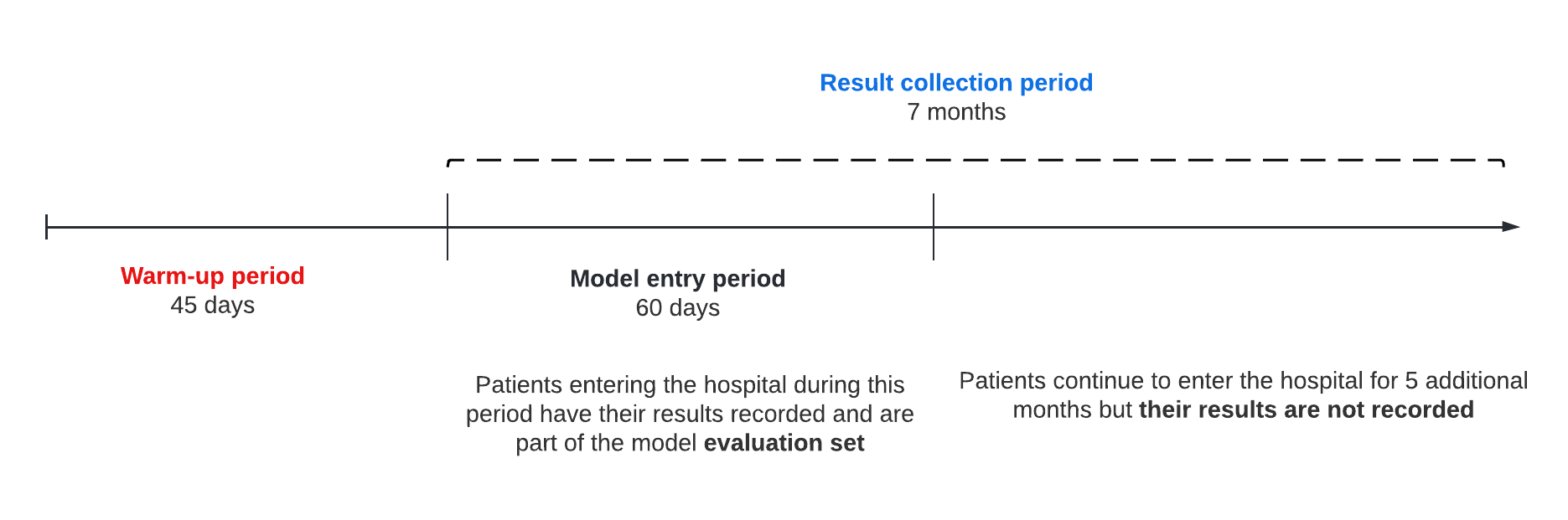


1. **Handling uncertainty**

Although the presented model is deterministic, *first-order* uncertainty was captured by running different iterations of the model 70 times – with each run using a different random number sequence. The example below highlights the importance of minimising *first-order* uncertainty of an event probability.

Patients entering the model are assigned a probability of having CDI which has a fixed mean value attributed to it (i.e. 10.342%). At the arrival of each new patient however, the DES modelling technique samples a random number from a uniform distribution (i.e. ‘throws the dice’), compares that random number to the assigned mean probability (0.10342) and uses this to determine whether a certain individual in the model has CDI or not. For example, assuming a random number of 0.10, this value is below the probability of having CDI (0.10342) and therefore the patient will be classed as having CDI. The opposite is true if the random number exceeds the probability of having CDI. Although the overall likelihood that a patient has CDI is set of a fixed mean, there is a *first-order* uncertainty introduced at the individual patient level (i.e. the fact that SIMUL8 ‘throws the dice’ at each patient’s arrival), which in part determines whether or not they are truly with CDI or not. In no-constraints DES models (e.g. with no limit on number of single beds), simulating a large number of patients entering the hospital will estimate on average the true number of CDI positive patients which, in turn, eliminates this issue. As such, in order to reduce the impact of this uncertainty within constrained-DES models, sufficient model iterations need to be run, using different random number sequences for each run of the model.

Since a probabilistic sensitivity analysis (PSA) was not conducted as part of the model analysis, *second-order* uncertainty^^[[6]](#footnote-6)^^ was not accounted for. Conducting a PSA for each possible value of diagnostic accuracy and test turnaround time within the analysis would have significantly increased the computational burden and extended the model running time. Extensive sensitivity and scenario analyses were instead conducted to explore the uncertainty around key parameters and clinical assumptions and its impact on model outcomes.

1. **Model validation**

The model structure and parameters underwent extensive validation, including:

- **face validity** – consultations with clinical experts ensured validity of the conceptual model and its input data;
- **internal validity** – the model was checked for any errors in coding by running extreme sets of parameters values (*extreme value testing*), replacing distributions with a constant number, and tracking patients through the model to assess whether the coding logic was correct *(testing of traces*). Model coding was also internally reviewed by an expert modeller within the Academic Unit of Health Economics, University of Leeds.
- **operational validity** – marginal and extreme changes to baseline parameters values were explored to assess: (i) how model parameters and structural assumptions affect the model outputs; and (ii) whether the model outputs would follow the expected trajectory following changes in parameters and structural assumptions. **Supplementary Figure 1. 7** gives a simplified schematic of the mechanisms by which model parameters and structural scenarios have an impact on model outputs

**Supplementary Figure 1. 7 Simplified schematic of the impact of model parameters and structural scenarios on clinical- and cost-effectiveness outputs. Disease- and testing-workflow-related parameters are associated with red and blue colourings, respectively. Sections highlighted in green and orange are associated with cost parameters and structural scenarios, respectively. Sections with an asterisk (*) are dependent on the size of the hospital being simulated**


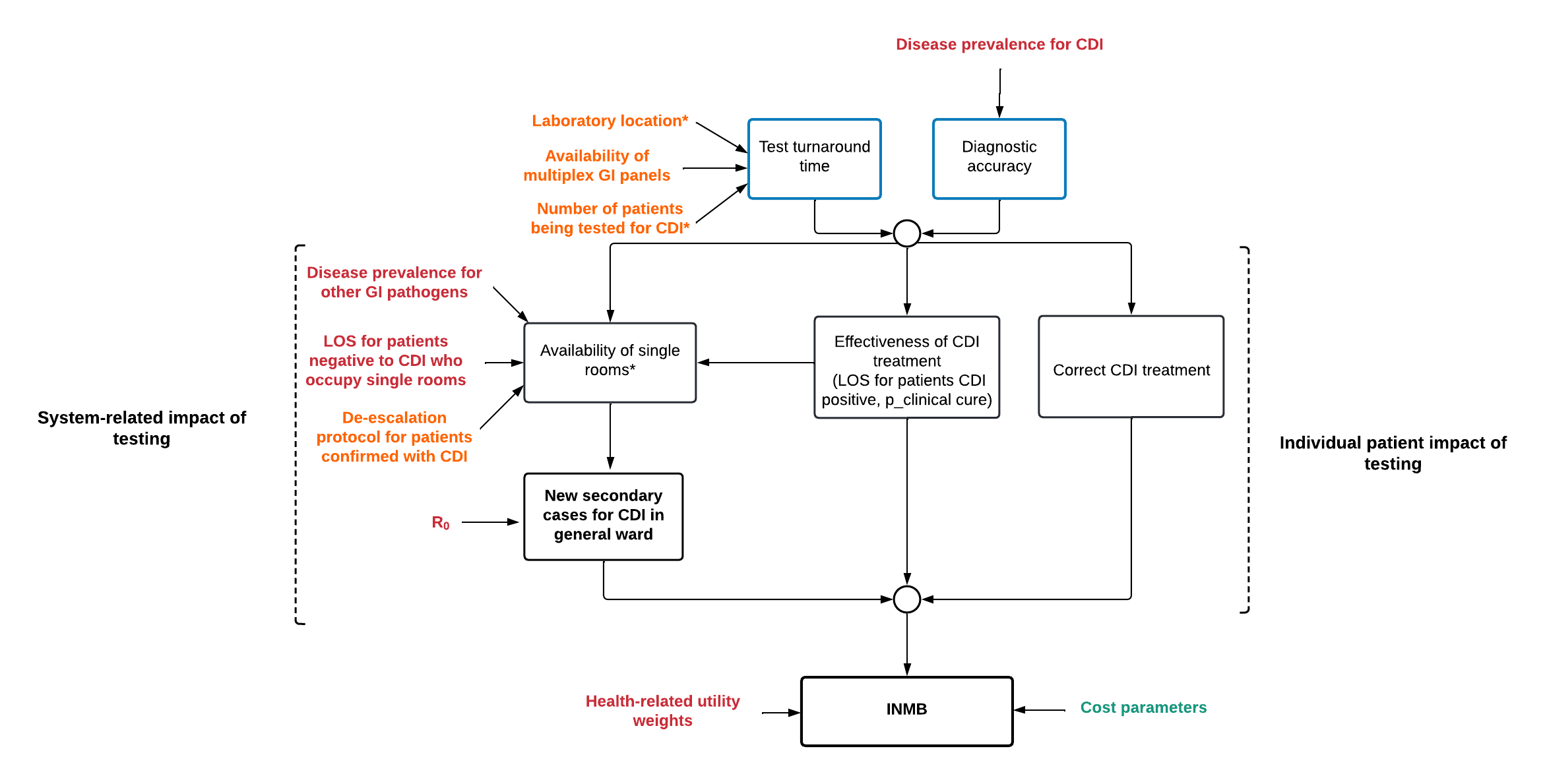


**1.6. References Appendix 1**

1. Barbut F, Surgers L, Eckert C, et al. Does a rapid diagnosis of *Clostridium difficile* infection impact on quality of patient management? *Clin Microbiol Infect* 2014; 20: 136-144. DOI: 10.1111/1469-0691.12221.

2. McFarland LV, Ozen M, Dinleyici EC and Goh S. Comparison of pediatric and adult antibiotic-associated diarrhea and Clostridium difficile infections. *World J Gastroenterol* 2016. DOI: doi:10.3748/wjg.v22.i11.3078.

3. Leeds Teaching Hospitals NHS Trust. Clostridium Difficile Infection ( CDI ) in Adults ( 16 years of age ), <http://www.lhp.leedsth.nhs.uk/detail.aspx?ID=1254> (2008, accessed 24/10/2020 2020).

4. Leeds Teaching Hospitals NHS Trust. Clostridium Difficile - Prevention of Transmission - in Adults and Children > 2 years with Clostridium Difficile Infection ( CDI ), <http://www.lhp.leedsth.nhs.uk/detail.aspx?ID=677> (2017, accessed 26/10/2020 2020).

5. Leeds Teaching Hospitals NHS Trust. Isolation - Infection Prevention & Control, <http://www.lhp.leedsth.nhs.uk/detail.aspx?ID=678> (2018, accessed 26/10/2020 2020).

6. Tenover FC, Baron EJ, Peterson LR and Persing DH. Laboratory diagnosis of Clostridium difficile infection can molecular amplification methods move us out of uncertainty? *J Mol Diagn* 2011; 13: 573-582. DOI: <https://dx.doi.org/10.1016/j.jmoldx.2011.06.001>.

7. Polage C, Gyorke CE, Kennedy MA, et al. Overdiagnosis of Clostridium difficile Infection in the Molecular Test Era. *JAMA Intern Med* 2015; 175: 1792-1801. DOI: 10.1001/jamainternmed.2015.4114.

8. Planche TD, Davies KA, Coen PG, et al. Differences in outcome according to *Clostridium difficile* testing method: a prospective multicentre diagnostic validation study of *C difficile* infection. *The Lancet Infectious Diseases* 2013; 13: 936-945. DOI: 10.1016/S1473-3099(13)70200-7.

9. Goldenberg SD, Bisnauthsing KN, Patel A, et al. Point-of-Care Testing for Clostridium Difficile Infection: A Real-World Feasibility Study of a Rapid Molecular Test in Two Hospital Settings. *Infect Dis Ther* 2014; 3: 295-306. DOI: 10.1007/s40121-014-0038-6.

10. Jones WS, Rice S, Power HM, et al. Cost Consequences for the NHS of Using a Two-Step Testing Method for the Detection of Clostridium difficile with a Point of Care, Polymerase Chain Reaction Test as the First Step. *Diagnostics* 2020; 10. DOI: <https://dx.doi.org/10.3390/diagnostics10100819>.

11. Maghdoori S and Moghadas SM. Assessing the effect of patient screening and isolation on curtailing Clostridium difficile infection in hospital settings. *BMC Infect Dis* 2017; 17: 384. DOI: 10.1186/s12879-017-2494-6.

12. Bartsch SM, Curry SR, Harrison LH and Lee BY. The potential economic value of screening hospital admissions for Clostridium difficile. *Eur J Clin Microbiol Infect Dis* 2012; 31: 3163-3171. DOI: <https://dx.doi.org/10.1007/s10096-012-1681-z>.

13. Bartsch SM, Umscheid CA, Nachamkin I, et al. Comparing the economic and health benefits of different approaches to diagnosing Clostridium difficile infection. *Clin Microbiol Infect* 2015; 21: 77.e71-79. DOI: <https://dx.doi.org/10.1016/j.cmi.2014.07.002>.

14. Boekhoud IM, Sidorov I, Nooij S, et al. Haem is crucial for medium-dependent metronidazole resistance in clinical isolates of Clostridioides difficile. *J Antimicrob Chemother* 2021. DOI: 10.1093/jac/dkab097.

15. Viprey VF, Davis GL, Benson AD, et al. Key Differences in Diagnosis and Patient Populations between Community and In-Patient Clostridioides Difficile Infections (CDI): Results from Combatting Bacterial Resistance in Europe CDI (COMBACTE-CDI). *Lancet* 2021. DOI: Available at SSRN: <https://ssrn.com/abstract=3812436> or <http://dx.doi.org/10.2139/ssrn.3812436>.

16. Delignette-Muller ML and Dutang C. fitdistrplus: An R Package for Fitting Distributions. *J Stat Softw* 2015; 64: 1 - 34. DOI: 10.18637/jss.v064.i04.

17. Wingen-Heimann S, Lurienne L, Davies K, et al. Healthcare resource utilisation for treatment of Clostridioides difficile infection across 12 European countries: health economic results of COMBACTE-CDI. *ECCMID*. 2020.

18. SIMUL8 Corporation. Probability Profile, <https://www.simul8.com/support/help/doku.php?id=features:distributions:probability_profile> (2022, accessed 12/07/2022 2022).

19. Freeman K, Mistry H, Tsertsvadze A, et al. Multiplex tests to identify gastrointestinal bacteria, viruses and parasites in people with suspected infectious gastroenteritis: a systematic review and economic analysis. *Health Technol Assess* 2017; 21: 1-188. DOI: <https://dx.doi.org/10.3310/hta21230>.

20. Szende A, Janssen MF and Cabases JM. *Self-Reported Population Health: An International Perspective based on EQ-5D*. 2014, p.1-196.

21. Wilcox MH, Ahir H, Coia JE, et al. Impact of recurrent Clostridium difficile infection: hospitalization and patient quality of life. *J Antimicrob Chemother* 2017; 72: 2647-2656. DOI: 10.1093/jac/dkx174.

22. McDonald LC, Gerding DN, Johnson S, et al. Clinical Practice Guidelines for Clostridium difficile Infection in Adults and Children: 2017 Update by the Infectious Diseases Society of America (IDSA) and Society for Healthcare Epidemiology of America (SHEA). *Clin Infect Dis* 2018; 66: e1-e48. DOI: 10.1093/cid/cix1085.

23. Lanzas C, Dubberke ER, Lu Z, et al. Epidemiological Model for Clostridium difficile Transmission in Healthcare Settings. *Infect Control Hosp Epidemiol* 2011; 32: 553-561. 2015/01/02. DOI: 10.1086/660013.

24. Crobach MJT, Planche T, Eckert C, et al. European Society of Clinical Microbiology and Infectious Diseases: update of the diagnostic guidance document for Clostridium difficile infection. *Clin Microbiol Infect* 2016; 22 Suppl 4: S63-81. DOI: <https://dx.doi.org/10.1016/j.cmi.2016.03.010>.

25. Planche T and Wilcox M. Reference assays for *Clostridium difficile* infection: one or two gold standards? *J Clin Pathol* 2011; 64: 1. DOI: 10.1136/jcp.2010.080135.

26. Eastwood K, Else P, Charlett A and Wilcox M. Comparison of Nine Commercially Available *Clostridium difficile* Toxin Detection Assays, a Real-Time PCR Assay for *C*. *difficile* *tcdB* and a Glutamate Dehydrogenase Detection Assay to Cytotoxin Testing and Cytotoxigenic Culture Methods. *J Clin Microbiol* 2009; 47: 3211. DOI: 10.1128/JCM.01082-09.

27. Techlab. C-DIFF-CHEK-60TM-60, <https://www.techlab.com/wp-content/uploads/2019/09/C-DIFF-CHEK-60_PI_91-392-03_read-version_08_2019.pdf> (2019, accessed 10/03/2021 2021).

28. Cepheid. Xpert C.difficile ENGLISH Package Insert <https://p.widencdn.net/zmmjln/Cepheid-Xpert-C-difficile-BT-Datasheet-CE-IVD-3073-English> (2019, accessed 10/03/2021 2021).

29. Cepheid. Xpert Cdifficile/Epi, <https://labymed.com.gt/wp-content/uploads/2020/06/XpertCdifficile-Epi.pdf> (2019, accessed 10/03/2021 2021).

30. Sewell B, Rees E, Thomas I, et al. Impact on patient length of stay and cost-effectiveness of rapid molecular testing for Clostridium difficile. *Clin Microbiol Infect* 2012; 18: 667. DOI: <http://dx.doi.org/10.1111/j.1469-0691.2012.03802.x>.

31. British National Formulary. VANCOMYCIN Medicinal forms <https://bnf.nice.org.uk/medicinal-forms/vancomycin.html> (accessed 20/05/2021 2021).

32. Tresman R and Goldenberg SD. Healthcare resource use and attributable cost of Clostridium difficile infection: a micro-costing analysis comparing first and recurrent episodes. *J Antimicrob Chemother* 2018; 73: 2851-2855. DOI: 10.1093/jac/dky250.

33. Food Drug Administration Agency. Evaluation of Aumatic Class III Designation (De Novo) For xTAG® Gastrointestinal Pathogen Panel (GPP) Decision Summary, [www.accessdata.fda.gov/cdrh_docs/reviews/K121454.pdf](file:///C:\Users\u2272102\OneDrive%20-%20University%20of%20Leeds\6.Model%20C.diff%20infection\Manuscript\MDM\RESUBMISSION\www.accessdata.fda.gov\cdrh_docs\reviews\K121454.pdf) (2012, accessed 09/02/2022 2022).

34. Buss S, Leber A, Chapin K, et al. Multicenter Evaluation of the BioFire FilmArray Gastrointestinal Panel for Etiologic Diagnosis of Infectious Gastroenteritis. *J Clin Microbiol* 2015; 53: 915-925. DOI: 10.1128/JCM.02674-14.

35. Korhonen L, Cohen J, Gregoricus N, et al. Evaluation of viral co-infections among patients with community-associated Clostridioides difficile infection. *PLoS One* 2020; 15: e0240549. DOI: 10.1371/journal.pone.0240549.

36. Shafiq M, Alturkmani H, Zafar Y, et al. Effects of co-infection on the clinical outcomes of Clostridium difficile infection. *Gut Pathog* 2020; 12: 9. DOI: 10.1186/s13099-020-00348-7.

37. Leeds Teaching Hospitals NHS Trust. Infections ( Alert Organisms And Conditions ) That Require Source Isolation, <http://www.lhp.leedsth.nhs.uk/detail.aspx?id=1671> (2009, accessed 26/10/2020 2020).

38. Zheng L, Keller SF, Lyerly DM, et al. Multicenter Evaluation of a New Screening Test That Detects *Clostridium difficile* in Fecal Specimens. *J Clin Microbiol* 2004; 42: 3837. DOI: 10.1128/JCM.42.8.3837-3840.2004.

39. Schroeder LF, Robilotti E, Peterson LR, et al. Economic evaluation of laboratory testing strategies for hospital-associated Clostridium difficile infection. *J Clin Microbiol* 2014; 52: 489-496. DOI: <https://dx.doi.org/10.1128/JCM.02777-13>.

40. Lewis BB and Pamer EG. Microbiota-Based Therapies for Clostridium difficile and Antibiotic-Resistant Enteric Infections. *Annu Rev Microbiol* 2017; 71: 157-178. DOI: 10.1146/annurev-micro-090816-093549.

41. Pollock NR. Ultrasensitive Detection and Quantification of Toxins for Optimized Diagnosis of Clostridium difficile Infection. *J Clin Microbiol* 2016.

42. Bank of England Inflation Calculator. <https://www.bankofengland.co.uk/monetary-policy/inflation/inflation-calculator> (accessed 05/03/2021 2021).

43. Stevenson M, Metry A and Messenger M. Modelling of hypothetical SARS-CoV-2 point of care tests on admission to hospital from A&E: rapid cost-effectiveness analysis. 2021; 25: 21. DOI: 10.3310/hta25210.

44. Department of Health. NHS reference costs 2014 to 2015, <https://www.gov.uk/government/publications/nhs-reference-costs-2014-to-2015> (2012, accessed 18/11/22 2022).

45. Minor T, Lasher A, Klontz K, et al. The Per Case and Total Annual Costs of Foodborne Illness in the United States. *Risk Anal* 2015; 35: 1125-1139. <https://doi.org/10.1111/risa.12316>. DOI: <https://doi.org/10.1111/risa.12316>.

46. Karnon J, Stahl J, Brennan A, et al. Modeling using Discrete Event Simulation: A Report of the ISPOR-SMDM Modeling Good Research Practices Task Force-4. *Value in Health* 2012; 15: 821-827. DOI: <https://doi.org/10.1016/j.jval.2012.04.013>.

47. Briggs AH, Weinstein MC, Fenwick EAL, et al. Model Parameter Estimation and Uncertainty: A Report of the ISPOR-SMDM Modeling Good Research Practices Task Force-6. *Value in Health* 2012; 15: 835-842. DOI: <https://doi.org/10.1016/j.jval.2012.04.014>.

48. RID-AMR@Leeds. Rapid infection diagnostics to combat antimicrobial resistance, <https://ridamr.leeds.ac.uk/> (2021, accessed 02/12/2021 2021).

49. Goldenberg SD, Bisnauthsing KN, Patel A, et al. Point-of-Care Testing for Clostridium Difficile Infection: A Real-World Feasibility Study of a Rapid Molecular Test in Two Hospital Settings. *Infectious Diseases and Therapy* 2014; 3: 295-306. DOI: 10.1007/s40121-014-0038-6.

50. Barbut F, Braun M, Burghoffer B, et al. Rapid detection of toxigenic strains of Clostridium difficile in diarrheal stools by real-time PCR. *J Clin Microbiol* 2009; 47: 1276-1277. 2009/02/25. DOI: 10.1128/JCM.00309-09.

51. DiPersio JR, Varga FJ, Conwell DL, et al. Development of a rapid enzyme immunoassay for Clostridium difficile toxin A and its use in the diagnosis of C. difficile-associated disease. *J Clin Microbiol* 1991. DOI: 10.1128/jcm.29.12.2724-2730.1991.

1. If a patient remains longer in single room isolation than the assigned LOS due to slow time-to-diagnosis, during the next check to assess resolution of symptoms, the model has the capacity to assess if the time the patient has spent within the simulation exceeds the assigned individual LOS. If so, the patient is assumed to be discharged. [↑](#footnote-ref-1)
2. Samples were collected only between 2018 and 2019. [↑](#footnote-ref-2)
3. A probability profile in SIMUL8 is defined as “a type of distribution that sets the probability (the percentage change) of a value being sampled from a distribution” 18. SIMUL8 Corporation. Probability Profile, https://www.simul8.com/support/help/doku.php?id=features:distributions:probability_profile (2022, accessed 12/07/2022 2022).. For example, a probability profile of 10.342% was assigned to the disease prevalence of CDI. This means that, within each model run, there is a probability of 10.342% that each patient entering the hospital has CDI, as opposed to a probability of 89.658% of not having CDI. [↑](#footnote-ref-3)
4. Other pathogens included: adenovirus, Campylobacter, Cryptosporidium, E.coli, Giardia, norovirus, rotavirus, salmonella and shigella 19. Freeman K, Mistry H, Tsertsvadze A, et al. Multiplex tests to identify gastrointestinal bacteria, viruses and parasites in people with suspected infectious gastroenteritis: a systematic review and economic analysis. *Health Technol Assess* 2017; 21: 1-188. DOI: https://dx.doi.org/10.3310/hta21230. [↑](#footnote-ref-4)
5. The median estimate of single rooms was applied to represent the availability of single rooms for a typical UK Teaching Hospital while reducing the variability within the results due to outlier observations. This model did not attempt to capture the uncertainty variability around the number of available single rooms across the participating sites to the COMBACTE-CDI survey. [↑](#footnote-ref-5)
6. *Second-order* uncertainty is also referred as *parameter uncertainty* – defined as “the uncertainty in the estimation of the parameter of interest” 47. Briggs AH, Weinstein MC, Fenwick EAL, et al. Model Parameter Estimation and Uncertainty: A Report of the ISPOR-SMDM Modeling Good Research Practices Task Force-6. *Value in Health* 2012; 15: 835-842. DOI: https://doi.org/10.1016/j.jval.2012.04.014..

   The impact of *parameter uncertainty* can be estimated by deterministic sensitivity analysis, or preferably, by running a PSA 47. Ibid.. [↑](#footnote-ref-6)
