# Supplementary material for: Early Economic Modeling to Inform a Target Product Profile: A Case Study of a Novel Rapid Test for Clostridioides difficile Infection
Source: MDM Policy Pract. 2024 Nov 22;9(2):23814683241293739. doi: 10.1177/23814683241293739 (PMC11585019; doi:10.1177/23814683241293739)
Supplement: sj-docx-2-mpp-10.1177_23814683241293739 – Supplemental material for Early Economic Modeling to Inform a Target Product Profile: A Case Study of a Novel Rapid Test for Clostridioides difficile Infection [file sj-docx-2-mpp-10.1177_23814683241293739.docx]

Appendix 2

For each item of the CHEERS and STRESS-DES checklists, this appendix provides a reference to the section(s) where to find additional information on the development of the model outlined in manuscript and Appendix 1.

**Supplementary table 2. 1 CHEERS checklist**

| **Topic** | **No.** | **Item** | **Location where item is reported** |
| --- | --- | --- | --- |
| **Title** |  |  |  |
|  | 1 | Identify the study as an economic evaluation and specify the interventions being compared. | Manuscript, Title |
| **Abstract** |  |  |  |
|  | 2 | Provide a structured summary that highlights context, key methods, results, and alternative analyses. | Manuscript |
| **Introduction** |  |  |  |
| **Background and objectives** | 3 | Give the context for the study, the study question, and its practical relevance for decision making in policy or practice. | Manuscript, Introduction |
| **Methods** |  |  |  |
| **Health economic analysis plan** | 4 | Indicate whether a health economic analysis plan was developed and where available. | Not applicable |
| **Study population** | 5 | Describe characteristics of the study population (such as age range, demographics, socioeconomic, or clinical characteristics). | Manuscript, Model structure. |
| **Setting and location** | 6 | Provide relevant contextual information that may influence findings. | Manuscript, Model structure |
| **Comparators** | 7 | Describe the interventions or strategies being compared and why chosen. | Manuscript, Model structure |
| **Perspective** | 8 | State the perspective(s) adopted by the study and why chosen. | Manuscript, Model structure |
| **Time horizon** | 9 | State the time horizon for the study and why appropriate. | Manuscript, Model analysis |
| **Discount rate** | 10 | Report the discount rate(s) and reason chosen. | Manuscript, Model analysis |
| **Selection of outcomes** | 11 | Describe what outcomes were used as the measure(s) of benefit(s) and harm(s). | Manuscript, Model analysis |
| **Measurement of outcomes** | 12 | Describe how outcomes used to capture benefit(s) and harm(s) were measured. | Appendix 1, section 1.3. |
| **Valuation of outcomes** | 13 | Describe the population and methods used to measure and value outcomes. | Appendix 1, section 1.3 |
| **Measurement and valuation of resources and costs** | 14 | Describe how costs were valued. | Appendix 1, section 1.2.10. |
| **Currency, price date, and conversion** | 15 | Report the dates of the estimated resource quantities and unit costs, plus the currency and year of conversion. | Appendix 1, section 1.2.10. |
| **Rationale and description of model** | 16 | If modelling is used, describe in detail and why used. Report if the model is publicly available and where it can be accessed. | Manuscript, Model Structure |
| **Analytics and assumptions** | 17 | Describe any methods for analysing or statistically transforming data, any extrapolation methods, and approaches for validating any model used. | Manuscript, Model Structure. |
| **Characterising heterogeneity** | 18 | Describe any methods used for estimating how the results of the study vary for subgroups. | Not applicable |
| **Characterising distributional effects** | 19 | Describe how impacts are distributed across different individuals or adjustments made to reflect priority populations. | Not applicable |
| **Characterising uncertainty** | 20 | Describe methods to characterise any sources of uncertainty in the analysis. | Manuscript, Model Analysis and Results, |
| **Approach to engagement with patients and others affected by the study** | 21 | Describe any approaches to engage patients or service recipients, the general public, communities, or stakeholders (such as clinicians or payers) in the design of the study. | Manuscript, Model Structure. |
| **Results** |  |  |  |
| **Study parameters** | 22 | Report all analytic inputs (such as values, ranges, references) including uncertainty or distributional assumptions. | Manuscript, Results |
| **Summary of main results** | 23 | Report the mean values for the main categories of costs and outcomes of interest and summarise them in the most appropriate overall measure. | Manuscript, Results |
| **Effect of uncertainty** | 24 | Describe how uncertainty about analytic judgments, inputs, or projections affect findings. Report the effect of choice of discount rate and time horizon, if applicable. | Manuscript, Results |
| **Effect of engagement with patients and others affected by the study** | 25 | Report on any difference patient/service recipient, general public, community, or stakeholder involvement made to the approach or findings of the study | Not applicable |
| **Discussion** |  |  |  |
| **Study findings, limitations, generalisability, and current knowledge** | 26 | Report key findings, limitations, ethical or equity considerations not captured, and how these could affect patients, policy, or practice. | Manuscript, Discussion |
| **Other relevant information** |  |  |  |
| **Source of funding** | 27 | Describe how the study was funded and any role of the funder in the identification, design, conduct, and reporting of the analysis | Manuscript, Title pages |
| **Conflicts of interest** | 28 | Report authors conflicts of interest according to journal or International Committee of Medical Journal Editors requirements. | Manuscript, Title pages |

**Supplementary table 2. 2** **STRESS-DES checklist**

| **Section/Subsection** | **Item** | **Details/location in Manuscript where details are reported** | |
| --- | --- | --- | --- |
| 1. **Objectives** |  | | |
| Purpose of the model | 1.1 | This study focused on the use of early economic modelling within Target Product Profiles (TPPs) as a means of informing key TPP performance requirements, for example diagnostic accuracy, turnaround time, and cost. Development of a full TPP for new diagnostic tests for CDI (including a wide range of additional elements such as analytical performance and infrastructural requirements) is part of a broader Medical Research Council (MRC)-funded programme grant (MR/N029976/1)^[[1]](#footnote-1)^, and is outside the scope of this study. The findings from this study will be used at later stages to inform parts of the full TPP developed within the MRC-funded project.  To explore the use of early economic modelling within TPP development, a case study is presented in this study focusing on a new rapid in-vitro point-of-care diagnostic test for Clostridioides difficile infection (CDI).  The aim of this study is to develop an early economic model to help inform key TPP characteristics for a new rapid diagnostic test for CDI (focusing on minimum diagnostic sensitivity and specificity, turnaround time and maximum cost for the test), based on the clinical utility and cost-effectiveness outputs of an early economic model. | |
| Model Outputs | 1.2 | The clinical effectiveness of each testing strategy was measured in terms of two key outputs: (1) quality-adjusted life year (QALY) gains; and (2) the number of secondary infections prevented.  Cost-effectiveness outputs of the model were expressed in terms of Incremental Net Monetary Benefit (INMB) from the UK NHS perspective (i.e. including direct healthcare costs), comparing each HT against standard care, using the NICE WTP lower threshold per QALY of £20,000. Clinical and cost-effectiveness outputs are calculated at the aggregate level – focusing on the total number of patients part of the *evaluation set* (i.e. those entering within the model entry period). | |
| Experimentation Aims | 1.3 | The experimental aims of this model were two-fold, including:   - explore the impact that a POCT could have on infection control infrastructure (e.g. availability of isolation rooms), clinical decision-making, infection spread (e.g. new secondary CDI cases in general ward) and costs; and - identify the necessary properties of a POCT for CDI to be cost-effective compared to standard care, from a UK NHS perspective (focusing on minimum diagnostic sensitivity and specificity, turnaround time and maximum cost for the test).   The findings of this early economic model should be considered exploratory since HT is a hypothetical test, no information is available currently on its diagnostic accuracy, turnaround time or test price. | |
| 1. **Logic** |  | | |
| Base model overview diagram | 2.1 | The structure of the model is presented in **Figure 1**.  The model tracks the flow of patients presenting with CDI symptoms through the hospital setting, accounting for key capacity constraints (i.e. availability of single rooms, laboratory schedule for processing samples). The upper branch of the model depicts the movement of patients through the hospital, while the lower branch depicts the testing processes undertaken on each patient’s test sample. In-hospital adult patients with an initial episode of acute diarrhoea for whom clinicians have requested stool testing for CDI enter the model.  Patients are moved into presumptive isolation, while their stool samples are tested simultaneously for CDI and other gastrointestinal (GI) pathogens which could cause diarrhoea. Stool testing and presumptive isolation happen simultaneously. Two locations are available for patients suspected with infectious diarrhoea depending on current capacity constraints:   - **single rooms**: only one patient can enter a single room with no potential of infecting others. If there is a confirmation of CDI, the patient remains in isolation until the end of their hospital LOS; and - **general ward**: when no single rooms are available, patients remain in the general ward while waiting for test result with a higher potential for infection transmission. The general ward is set to an unlimited capacity in the model, to host as many patients as possible, where necessary.   Cohort bays are available for patients confirmed with infectious diarrhoea only – in the absence of available single rooms. At LTHT, patients with the same detected infection can be grouped together in four cohort bays (each with a maximum capacity of 6 patients).  Upon sample collection, the sample is shipped to the laboratory or tested within the ward depending on the testing strategy under evaluation. The following features are common to both testing strategies being evaluated: (i) test turnaround time represents how long it takes to yield test results after having obtained the sample to test; (ii) the sample is simultaneously tested for CDI and other GI pathogens to rule out other causes of infectious diarrhoea using a rapid multiplex GI panel with perfected diagnostic accuracy; and (iii) upon receipt of final diagnosis, the test sample is matched to the corresponding patient within the patient isolation pathway, to update information on patient’s health following test results.  Upon receipt of a patient’s test results, clinicians can decide to: (i) continue isolation; (ii) de-escalate isolation measures and move the patient into the general ward; or (iii) discharge the patient if the patient has spent their assigned LOS in isolation^[[2]](#footnote-2)^.  If a patient presents with severe symptoms of CDI (e.g. evidence of severe colitis, high temperature), clinicians are assumed to start empirical antibiotic treatment before placing the patient into presumptive isolation and prior to receipt of test results (283). For patients with mild or moderate symptoms (e.g. approximately 3-5 stools per day), antibiotic treatment for CDI (i.e. vancomycin) starts upon receipt of positive test results for CDI.  This model evaluates two testing strategies, including:   - **Standard care testing for CDI** - A two-step testing algorithm is currently run at LTHT for patients suspected with CDI. Each sample is initially screened with TECHLAB ® C.DIFF CHECK^TM^-60 GDH EIA to detect the presence of *C. difficile* organism. If a sample is negative to GDH EIA, it is possible to exclude CDI. If a sample is positive on GDH screening, a Cepheid ® Xpert *C. difficile* PCR and CCNA are used simultaneously to detect toxin genes related to CDI or free toxins in stools, respectively. PCR testing yields results quickly (e.g. 43 minutes), whilst CCNA ultimately confirms the presence of free toxins within two days. Final diagnosis is confirmed upon receipt of CCNA results. - **Hypothetical rapid test (HT)** – a ward-based hypothetical POCT detecting toxins in stools. Various scenarios and sensitivity analyses were run in the model varying test turnaround time and diagnostic accuracy, as outlined in the section 2.3.   All patients, regardless of arm, exit the models once they have spent the assigned individual LOS within the simulation. | |
| Base model logic | 2.2 | In-hospital adult patients with an initial episode of acute diarrhoea for whom clinicians have requested stool testing for CDI enter the model. Two unique label identifiers ‘Patient_ID’ and ‘lbl_sampleID’ are assigned to all new arrivals at the ‘Starting Point’ to track patients for internal validation purposes. For each new arrival, the following labels are assigned   - lbl_time at entry = to track when patients enter the model; - lbl_true disease CDI = depending on the probability profile for disease prevalence, patients either (i) truly have CDI or (ii) truly do not have CDI. - lbl_clinical symptoms = set to 1 since all patients entering the model are symptomatic. The value of this label would change once the patient becomes asymptomatic. - lbl_intervention = set equal to global variable ‘Test Strategy’ (1= HT, 2 = standard care). - lbl_priority = set equal to 0 as patients entering the model are assigned a lower priority compared to patients confirmed with infectious diarrhoea while staying in the general ward. - Time to allocation isolation = set equal to 240 SIMUL8 minutes (4 hours)   At the **‘Starting Point’**, the model records KPIs only for patients entering after the end of warm-up period and up to the following 2 months (i.e. 24000 minutes). If a new patient enters simulation between this time period, set lbl_evaluation set equal to 1. This means that a certain patient is part of the Evaluation Set. Different activities will record KPIs only for those patients that belong to the Evaluation Set. In addition, the model assigns disease prevalence for other GI pathogens, hospital length of stay and CDI disease severity depending on the true disease status for CDI. If a patient is truly CDI positive (lbl_true disease CDI = 1), the model performs the following tasks:   - Set lbl_true disease GI pathogens equal to 2 as it is not possible to be positive also to other GI pathogens (no risk of co-infection) - Set lbl_CDI disease severity equal to dist_CDI disease severity   If a patient is truly CDI negative (lbl_true disease =2), the model performs the following tasks:   - Set lbl_true disease GI pathogens equal to dist_true disease GI pathogens. Assuming that there is no risk of co-infection, only CDI negative patients can be positive for other causes of infective diarrhoea. - Set lbl_LOS CDI Neg equal to dist_Hospital LOS Negative * 600. The distribution is originally defined in days, so it is necessary to convert it into SIMUL8 days (1 day=600 min in SIMUL8) - For the patients entering the model within the evaluation period, check if the assigned lbl_LOS CDI Neg is greater than the total results collection period. If the lbl_LOS CDI Neg is greater than the results collection period, reduce the assigned lbl_LOS CDI Neg by one month (12000 SIMUL8 minutes). This will ensure that patients having a long LOS exit the simulation and that their costs are captured within the analysis. As a Weibull distribution was used to fit the LOS data for CDI Negative patients, the distribution is skewed to the right with a long tail – this means that some patients might be assigned an extended LOS.   Patients are moved into presumptive isolation, while their stool samples are tested simultaneously for CDI and other GI pathogens which could cause diarrhoea. Stool testing and presumptive isolation happen simultaneously. The simulation software has the functionality to divide a single entity (i.e. patient) into two parts (called batching in SIMUL8) which share the same individual-level information (e.g. disease prevalence, time to enter the model), and to re-combine those two parts (called components) of an entity at a later event (i.e. once test results are back). This enables the user to simulate different events happening simultaneously to a single entity. This approach is used in the model to track: (i) which single isolation room a patient enters, depending on the current availability of single rooms, and (ii) the various processes of the testing pathway that each individual’s test sample undergoes (e.g. sample preparation, setting the machine, reviewing test results). The division of entities into their two respective components is undertaken at the start of the model. Once the patient’s test result is received, the two components are recombined and information on an individual patient’s health is updated. In the **‘Dummy Pre Testing’** activity, the Routing Out On Work Complete divides each entity into 2 components – Batching size equal to 2.  **A. Clinical Pathway**  Based on the LTHT clinical guidelines, a patient should be placed in isolation within 4 hours from suspicion of infectious diarrhoea. The **‘Queue for Allocate isolate’** is set to Prioritize based on lbl_priority. Assume lbl_priority is 0 for patients once they enter the simulation and 1 after they have been confirmed as having infectious diarrhoea in general ward.  **A.1. ‘Allocate isolation’**  The activity **‘Allocate isolation’** determines where a patient would move next depending on (i) availability of free single rooms and cohort bays, (ii) presence of patients with confirmed infectious diarrhoea with higher priority and (iii) time to receive test result. If the time elapse between time to obtain stool sample and time to receive final diagnosis is less than 2 hours, a patient is not placed in presumptive isolation. Isolating a patient for less than 2 hours might be a waste of resources as the same side room could be used for other patients in need [2]. In case of negative test result and clearance of symptoms, a patient might stay in General Ward without being empirically isolated.  There are two locations where patients suspected with infectious diarrhoea could be placed into isolation: (1) Single Rooms: capacity 1 patient, 93 rooms available [base case scenario]; (2) General Ward: infinite capacity.  The Route in VL, After Loading Work for **‘Allocate isolation’** performs the following tasks:   - **Setting Single Rooms Current Capacity** = Check current capacity in each single room and define total Single Rooms capacity. If total Single Rooms capacity is equal to 93, there are no single rooms available. Since all single rooms are full, the model will later (in Routing Out) try to allocate a patient in General Ward – or, in case of confirmed infectious patients (high priority), in Cohort Bays if available. - **Cohort Bays Current Capacity** = Similarly, check current capacity in each Cohort Bay and define total Cohort Bays capacity. If total Cohort Capacity is equal to 24 (4 cohort bays available x 6 patients each = max 24 patients can be placed in cohort bays), set Cohort Bays Full Capacity equal to 1. Only patients with confirmed infectious diarrhoea can be placed into cohort bays. - If Single Room Full Capacity is 1 (i.e. no single rooms are available), call **Single Rooms Lower Priority VL**. This VL checks if there are GI positive patients who are no longer symptomatic in any of the occupied single rooms. From an infection-control perspective, these patients are considered a lower priority thereby they can be de-isolated in case there is need of single rooms. Taking as an example **‘Single Room 1’** (note that this coding applies to each side room) - If a GI positive asymptomatic patient is in **‘Single Room 1’**, when test results are reviewed or at every time clinicians check presence of symptoms, a global variable called Single Room 1 Lower Priority will be set to 1. - If Single Room 1 Lower priority is set to 1, check if there is a patient in **‘Single Room 1’**. If yes, use command Select Current Work Item, estimate how long that patient has spent in isolation and then de-isolate that patient moving them into queue for Setting General Ward. Set Single Room 1 Lower Priority equal to 0 (since the patient has left this side room) and calculate Single Room 1 Capacity to keep this information updated   The Route Out VL, Work Complete for **‘Allocate isolation’** performs the following tasks   - **Sample ID Rapid Diagnosis VL** = Check if any of the patients waiting to be moved into presumptive isolation had received test results back within 2-hour window from request of stool testing (lbl_rapid diagnosis =1). If the time elapse between time to obtain stool sample and time to receive final diagnosis is less than 2 hours, a patient is not placed in presumptive isolation. Isolating a patient for less than 2 hours might be a waste of resources as the same single room could be used for other patients in need [2]. Clinicians are assumed to act upon test results and symptoms clearance. - If patient has not received test result yet (lbl_rapid diagnosis = 0), then this VL checks if the patient has been assigned a high priority from an infection control perspective. - HIGH PRIORITY (lbl_priority = 1) – the model checks if there are any single rooms available. If not, the next task would be to estimate if there are any beds available within Cohort Bays   - - If Cohort Bays are full, move patient into General Ward     - If there is an empty bed in one of the Cohort Bays, call **Cohort Bay Allocation High Priority VL** - CDI Pos Patients (includes Lbl_CDI Final diagnosis = 1 [TPs] or 4 [FPs]) – call **Cohort Bay Re Allocate CDI Pos (Allocate isolation) VL**. Estimate current capacity in Cohort Bay 1 and 2. If there are less than 6 patients in Cohort Bay 1, move this patient into Cohort Bay 1. Otherwise check capacity in Cohort Bay 2 – if there are less than 6 patients, move this patient into Cohort Bay 2. Otherwise, patient will move again into General Ward. - GI Pos Patients (i.e. lbl_GI panel testing outcome =1) – call **Cohort Bay Re Allocate GI Pos (Allocate isolation) VL**. Estimate current capacity in Cohort Bay 3 and 4. If there are less than 6 patients in Cohort Bay 3, move this patient into Cohort Bay 3. Otherwise check capacity in Cohort Bay 4 – if there are less than 6 patients, move this patient into Cohort Bay 4. Otherwise, patient will move again into General Ward. - If patient is CDI negative GI negative, stop simulation as patients without (confirmed) infectious diarrhoea should not be placed in Cohort Bays. - If Single Rooms and Cohort Bays are in full capacity, move patient into General Ward - NO PRIORITY (lbl_priority = 0) - patients suspected with infectious diarrhoea awaiting test results**.** the model checks if there are any single rooms available. If not, the patient would move to General ward. Count how many patients within the evaluation period are placed in General Ward instead of Side Rooms while waiting for test results. - Check CDI disease severity. If patient presents with severe symptoms (lbl_CDI disease severity =1) provide empirical treatment and set lbl_time to start CDI treatment equal to Simulation time. If patient has entered the simulation within evaluation count number of empirical treatments provided.   The Action VL for **‘Allocate isolation’** Call Single Rooms and Cohort Bays Full Capacity Time VL .This VL counts how many minutes both single rooms and cohort bays are in full capacity  **A.2. ‘Rapid diagnosis’**  Only patients who got test results within 120 minutes from stool testing request enter this activity. This activity matches the patient being processed in **‘Allocate isolation’** and sample with information on test results coming from **‘Dummy Post Testing’.** Once patient and test result/sample are matched, and it is possible to decide whether to move the patient in isolation depending on test result and clearance of symptoms  The Route Out, On Work Complete VL for **‘Rapid diagnosis’** performs the following tasks:   - **Check symptoms resolution (general) VL** - check if patient is still symptomatic depending on the timespan a patient has already spent in the simulation and time to clear symptoms (individual value drawn from a distribution), and test result for CDI. - Call **Check symptoms resolution (general) VL**– check where a patient can be placed in isolation   - SYMPTOMS DISAPPEAR (lbl_clinical symptoms = 2) - check if patient has tested negative to other GI pathogens - If not, check patient again in 2 days (1200 SIMUL8 minutes). At this stage, set lbl_time to next check (isolation) and (general ward) equal to 2 days as the current capacity of single rooms/cohort bays is currently unknow.   - Call **Check symptoms resolution (general) VL** to check where a patient can be placed in isolation - If patient is negative to other GI pathogens, call **Symptoms disappear: Review test results (rapid diagnosis) VL** - Check if lbl_CDI Final diagnosis = 1 or 4 (i.e. patient has tested positive to CDI)   - If not, CDI negative patient should not be isolated. Move patient into General ward   - If yes, end CDI treatment if it has already been started.   - CDI positive asymptomatic patients are assumed to remain in isolation/general ward until they are discharged.  - If patients have already spent their length of stay in isolation/simulation, they should be discharged. If patients have some time left, call **Check symptoms resolution (general) VL** to check where a patient can be placed in isolation.   - SYMPTOMS DISAPPEAR AND PATIENT HAS ALREADY SPENT LOS WITHIN THE SIMULATION (lbl_clinical symptoms = 3) – call De-escalation (rapid diagnosis) VL   - - Call **Reset Sample ID Rapid Diagnosis** – so that new patients can be assigned these IDs if needed       - - i.e. If Sample ID XX Rapid Diagnosis = Patient ID; SET Sample ID XX Rapid Diagnosis = 0. For XX = 0 to 30.   The Action VL for **‘Rapid diagnosis’** counts how many times presumptive isolation was avoided within the evaluation period when patients are not placed in isolation because there is confirmation of non-infective diarrhoea.  **A.3. ‘Setting Data Single Rooms’ activity**  This activity estimates how many single rooms are available, counts how many patients within the evaluation period are moved into isolation in single room and checks which single room is empty so that to move the next patient there using Routing Out based on label value. Call Allocation Single Rooms VL.  **A.4. ‘Setting Data Cohort Bay’ activity**  This activity estimates the current capacity within cohort bays. It checks if there are six patients already in Cohort Bay 1 and if not move patient to Cohort Bay 1 otherwise it checks if there are six patients already in Cohort Bay 2. This process is repeated until Cohort Bay 4. This activity counts how many patients within the evaluation period are moved into isolation and how many are moved within Cohort Bays.  **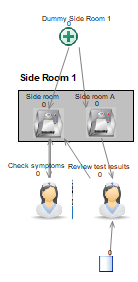A.5. ‘Single Room’ activities**  Each single room is divided into 2 separate queues. From a computational perspective, the model considers both queues as part of a single room. For example, Single Room 1 is divided into   - - **Single Room A**: where patients in presumptive isolation while waiting for test results. Set Single Room 1 Patient ID equal to individual PatientID (label). Once test results are ready, Dummy Post Testing activity will check through the global variables Single Room (1-93) Patient ID to see if a certain patient has been moved into Side Room 1. If so, Dummy Post Testing activity will route out the entity with test results info towards Side Room 1 to match test results with the patient awaiting in presumptive isolation   - **Single Room** **1**: where patients who have received test results are placed. Patients with confirmed infectious diarrhoea (either CDI or GI pathogens-related) remain in single room upon receipt of test results   The ‘Dummy Single Room X’ activities assess if the single room X is empty and route out patients only if single room X is free. These activities also move patients with confirmed infectious diarrhoea who have already received positive test result (lbl_rapid diagnosis = 1) directly to Single Room 1 as there is no need for presumptive isolation.  Once test results are back, Review test results activity matches the patient being in presumptive isolation (e.g. in Single Room A) and the entity with information on test results coming from Dummy Post Testing activity. Review test results activity decides whether to keep a patient in isolation, move them to General Ward or to discharge them depending on both test results and clearance of symptoms   - **Test positive for either CDI or other GI pathogens**: patient will stay in isolation. Patients with confirmed CDI (true-positive, false-positive) will stay in isolation for the duration of their hospital stay. Patients with other GI pathogens will stay in isolation up to 2 days from symptoms resolution and then might enter General Ward or be discharged depending on their individual length of stay. - **Test negative (for both CDI or GI pathogens)**: patient will be deisolated and subsequently enter General ward. This depends on patient’s hospital length of stay and how long they have spent in isolation.   If a patient is staying in isolation upon receipt of positive test result, set minimum waiting time for queue Single Room 1 according to their length of stay or time to next check.  Check symptoms activity assesses presence of symptoms on a daily basis (every 600 minutes) and decides whether to keep a patient in isolation depending on test results and clearance of symptoms  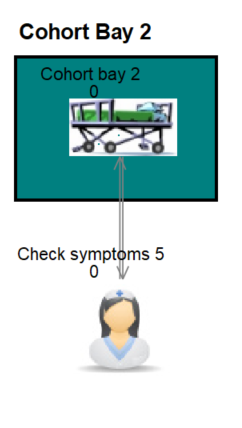**A.5. ‘Cohort Bay’ activities**  Four Cohort Bays are available. CDI positive patients are placed in Cohort Bay 1 and 2 only, whereas GI positive patients are placed in Cohort Bay 3 and 4 only. According to LTHT clinical practice, it is recommended to avoid mixing between CDI positive and GI positive patients. A maximum of 6 patients can be grouped together within a given Cohort Bay (e.g. Cohort Bay x). A new patient being allocated into isolation enters Cohort Bay x if there are less than 6 patients.  Check symptoms activity assesses presence/resolution of symptoms and decides whether to keep a patient in isolation depending on   - Test results and clearance of symptoms (logic is similar to the one for Single Rooms) - Whether any of the single rooms are currently available - every half a day the simulation will check if patients in Cohort Bays can be decanted into Single room isolation depending on current availability of single rooms.   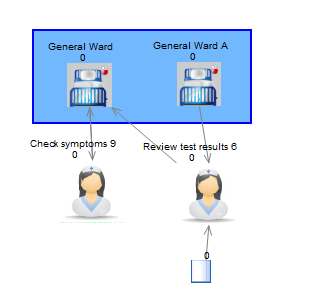  **A.6. ‘General Ward’ activities**  Three patient subgroups enter General Ward:   - patients who received confirmation of infectious diarrhoea within 2 hours from stool testing request (lbl_rapid diagnosis =1) but were not moved into isolation in single Rooms as there were no free single rooms nor Cohort Bays available; - patients who are waiting for test results and are being moved into General Ward as there were no free single rooms available; - patients who had been already isolated in single rooms/Cohort Bays but are going to stay in General Ward for the remaining of their length of stay (because they are either asymptomatic or CDI-/GI-)   If a patient has already received test result, the simulation checks if patients have already spent their LOS in isolation   - If yes, discharge patient (difference between LOS and time spent in isolation will be equal to 0 or negative) - If not, patient will stay in General Ward for the remaining LOS   The General Ward is divided into 2 separate queues. From a computational perspective, the model considers both queues as part of the General Ward. For example, Single Room 1 is divided into   - - **General Ward A**: where patients waiting for test results are placed. Set any of the General Ward Patient IDs equal to the PatientID individual label value. This information will be used later within Dummy Post Testing activity to match individual patient test results with patient’s location. If General Ward Patient ID 1 has already been assigned, check General Patient ID 2 until General Ward ID 104 (there is infinite capacity within General Ward). Once test results are ready, a certain patient will exit queue for presumptive isolation. Reset General Ward Patient ID equal to 0 so that new patients entering this queue can be assigned this global variable.   Example: Patient 2398 entered General Ward and there were already 10 patients awaiting in presumptive isolation. General Ward Patient ID 11 is now equal to 2398, as General Ward Patient ID 1-10 global variables are set equal to PatientID labels of the patients already within General Ward A. once test results for Patient 2398 are ready, Patient 2398 will exit queue for presumptive isolation. General Ward Patient ID 11 is now set to 0 since Patient 2398 is no longer within this queue.   - - **General Ward**: where patients who have received test results are placed – due to lack of availability of free single rooms and/cohort bays or because CDI negative or GI pathogens negative.   **‘Review test results 6’** activity matches the patient waiting in General Ward A with the test result information based on PatientID label. It then decides whether to keep a patient in isolation based on symptoms clearance and test results. If a patient within the general ward is confirmed with infectious diarrhoea, they may be transferred into a single room or cohort bay (depending on availability) to reduce risk of transmission. These patients will be assigned a higher priority from an infection-control perspective over patients suspected with infectious diarrhoea awaiting to be placed in presumptive isolation. Alternatively, when single rooms and cohort bays are at full capacity, confirmed cases remain in the general ward until hospital discharge. The simulation then counts the total number of infectious CDI patients (i.e. TP and FN) within general ward over the model evaluation period.  **‘Check symptoms 9’** activity checks if the patient being evaluated is still symptomatic (call Check symptoms resolution (general) VL). Patient will remain in General Ward depending on the following information:   - - Presence/clearance of symptoms   - Test results for both CDI and other GI pathogens   - Length of hospital stay   **A.7. Discharge**  The **‘Clinic assessment: discharge’** activity performs the following tasks:   - It routes out patients depending on their diagnosis of CDI and other GI pathogens. - For those patients within the evaluation set, store individual labels into Internal spreadsheet (call **Exporting Individual Labels into Spreadsheet**) - Count number of patients within the evaluation set exiting the simulation   - - **CDI disease severity VL** – to estimate how many patients had mild, moderate and severe CDI symptoms     - **CDI final diagnosis VL** – to define how many patients were TP,FP,TN,FN     - **Isolation and General Ward Bed Costs VL** – to estimate bed costs     - **CDI treatment regimen VL** – to estimate how long/and if patients have received CDI treatment and calculate costs     - **Count unjustified isolation_treatment days VL** – estimate how many unnecessary isolation and treatment days have occurred within the evaluation period     - **Testing Costs, Bed Costs, Treatment Costs and Total Costs VL**     - **Total QALY calculations (general) VL** – QALY calculations VL coding is dependent on the CDI diagnosis   **B. Testing workflow**  **B.1. Stool Sampling**  This activity takes on average 0.5 day (300 minutes) based on a triangular distribution. It performs the following tasks:   - It counts how many samples are being tested within evaluation period - It defines the individual time to clear symptoms based on distribution diarrhoea duration after sampling * 600. This is because original distributions are set in days, and it is necessary to convert them into SIMUL8 minutes (1 day = 600 minutes in SIMUL8). Distribution of diarrhoea duration is dependent on true disease CDI status.   **B.2 Sample Transportation**  This activity transports the sample from the ward to the laboratory or another location within the ward (if testing strategy is ward-based). It also routes out samples to either HT or LTHT standard testing strategy depending on the testing strategy being evaluated.  **B.3. HT – testing strategy**  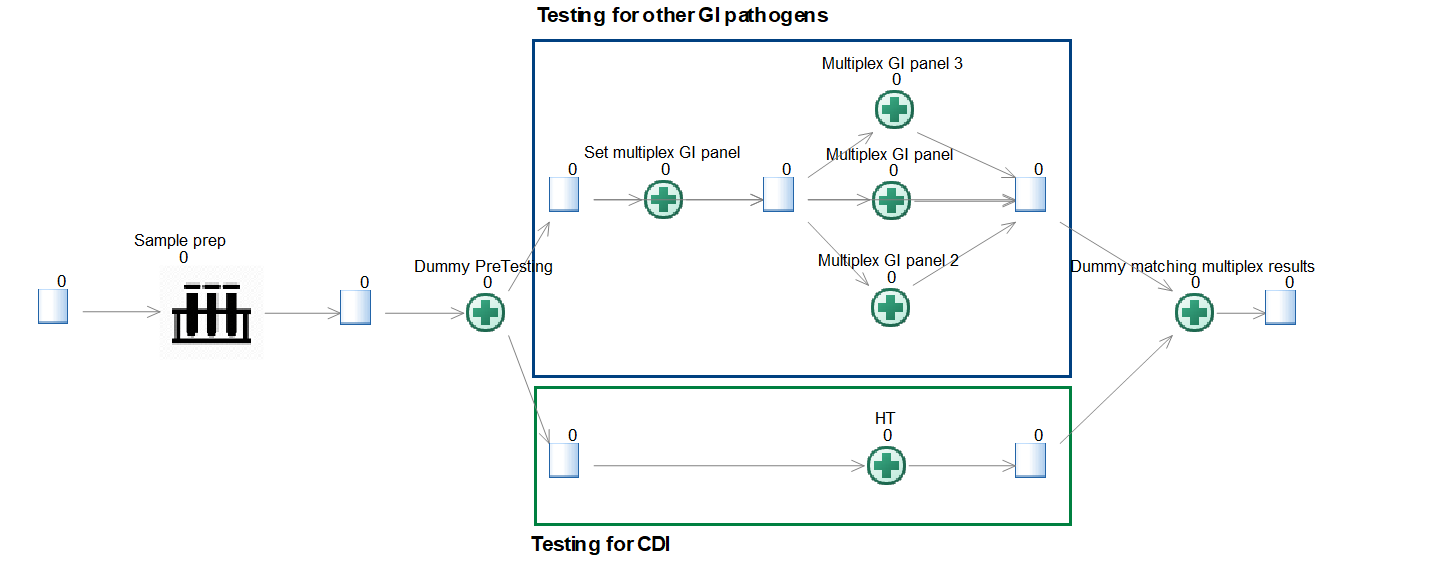  The activity **‘Sample preparation’** prepares the samples to be tested over an average of 30 minutes on average. All samples waiting to be prepared are collected at once (there is no fixed batch size).  The activity **‘Dummy PreTesting’** duplicates 1 sample into 2 entities that will be processed simultaneously by a multiplex GI panel and a POCT detecting toxins.  The activity **‘HT’** determines the individual diagnosis of CDI depending on the individual’s true disease status for CDI and the diagnostic sensitivity and specificity of the test. This activity then sets the lbl_HT outcome accordingly   - lbl_HT outcome =1 🡪 true-positive - lbl_HT outcome = 2 🡪 false-negative - lbl_HT outcome = 3 🡪 true-negative - lbl_HT outcome = 4 🡪 false-positive   The activity **‘HT’** also counts the number of HT tests run within the evaluation period, and the number of TP, FN, TN, FP cases.  The activity **‘Set multiplex GI panel’** prepares samples to be tested with multiplex GI panel with an operating time equal to PCR hands-on time.  The activities **‘Multiplex GI panel X’** determine the individual diagnosis of infectious diarrhoea due to other GI pathogens depending on the individual’s true disease status for other GI pathogens and the diagnostic sensitivity and specificity of the test. This activity then sets the lbl_GI panel testing outcome accordingly   - lbl_ GI panel testing outcome =1 🡪 true-positive - lbl_ GI panel testing outcome = 2 🡪 false-negative - lbl_ GI panel testing outcome = 3 🡪 true-negative - lbl_ GI panel testing outcome = 4 🡪 false-positive   Multiplex GI panels are assumed to be perfected diagnostic accuracy. The activities **‘Multiplex GI panel X’** also counts the number of multiplex GI panel tests run within the evaluation period, and the number of TP, FN, TN, FP cases.  The activity **‘Dummy matching multiplex results’** matches 2 samples being tested simultaneously with multiplex PCR and toxin POCT based on Patient ID. This activity also calculates Final Diagnosis TAT as the time difference between when stool sample is ready and when test results are back  **B.4. LTHT Testing Algorithm**  The activity **‘Sample preparation’** prepares the samples to be tested over an average of 30 minutes on average. All samples waiting to be prepared are collected at once (there is no fixed batch size).  The activity **‘Dummy PreTesting’** duplicates 1 sample into 2 entities that will be processed simultaneously by a multiplex GI panel and a GDH/PCR/CCAN testing algorithm.  The activity **‘GDH’** performs the following tasks:   - It collects all samples waiting to be tested for GDH at once. There is no fixed batch size for this activity to function; - It counts how many times GDH testing kits have been used within the evaluation period, regardless of the number of samples being tested - It determines the individual diagnosis of CDI depending on the individual’s true disease status for CDI and the diagnostic sensitivity and specificity of the test. This activity then sets the lbl_GDH outcome accordingly   - lbl_GDH outcome =1 🡪 true-positive   - lbl_GDH outcome = 2 🡪 false-negative   - lbl_GDH outcome = 3 🡪 true-negative   - lbl_GDH_outcome = 4 🡪 false-positive - For GDH Negative samples (TN,FN), results are automatically sent back to clinicians through Laboratory Information Management System. This means that in the Screening assessment activity, operation time is set equal to 0 since results are automatically sent without a biomedical scientist reviewing GDH Positive results - For GDH Positive samples (TP,FP), a biomedical scientist reviews the test results. This takes 30 minutes   The activity **‘Screening assessment’** routes samples out depending on the respective test result (GDH Positive vs GDH Negative):   - GDH Positive samples need to be tested for the presence of toxins. Move towards Dummy toxins testing - GDH Negative samples: CDI can be ruled out, so the simulation matches this sample with results from other GI pathogens to rule out any causes of infectious diarrhoea. This activity counts the number of missed CDI treatment and treatment correctly NOT administered within evaluation period in case of FN and TN GDH test results, respectively.   The activity **‘Dummy toxin testing’** performs the following tasks:   - It collects all samples waiting to be tested for toxins at once. This activity waits until there are no entities being processed in the Screening activity assessment. This means that the biomedical scientist has reviewed all GDH positive samples. - It counts (i) how many samples are being tested for toxins; (ii) how many samples are being test with PCR and CCNA simultaneously.   The activity **‘Dummy PCR CCNA testing’** duplicates 1 sample into 2 entities that will be processed simultaneously by PCR and CCNA  The activity **‘Setting PCR machine’** collects all samples waiting to be tested for PCR at once.  The activity **‘PCR’** performs the following tasks:   - It collects maximum 4 samples waiting to be tested for PCR at once - It determines the individual diagnosis of CDI depending on the individual’s true disease status for CDI and the diagnostic sensitivity and specificity of the test. This activity then sets the lbl_PCR outcome accordingly   - lbl_PCR outcome =1 🡪 true-positive   - lbl_PCR outcome = 2 🡪 false-negative   - lbl_PCR outcome = 3 🡪 true-negative   - lbl_PCR_outcome = 4 🡪 false-positive - It counts how many times PCR has been used within the evaluation period, regardless of the number of samples being tested. - Final diagnosis for CDI is confirmed upon receipt of CCNA results which usually take longer to get back. A negative PCR result is not sufficient to rule out the diagnosis of CDI so it is necessary to wait until CCNA results are back. In the meantime, patients will continue to remain in presumptive isolation awaiting test results.Then PCR and CCNA results are matched.   The activity **‘Prepare CCNA’** collects all samples waiting to be tested with CCNA at once.  The activity **‘CCNA’** performs the following tasks:   - It determines the individual diagnosis of CDI depending on the individual’s true disease status for CDI and the diagnostic sensitivity and specificity of the test. This activity then sets the lbl_CCNA outcome accordingly   - lbl_CCNA outcome =1 🡪 true-positive   - lbl_CCNA outcome = 2 🡪 false-negative   - lbl_CCNA outcome = 3 🡪 true-negative   - lbl_CCNA_outcome = 4 🡪 false-positive - It counts how many times CCNA has been prepared and ran within the evaluation period, regardless of the number of samples being tested.   The activity **‘Match PCR and CCNS result’** matches PCR positive samples with CCNA samples and it defines the Final diagnosis taking into consideration both CCNA and PCR result. This activity also counts the TP,FN, TN, FP, correct/missed/inappropriate/correctly NOT administered CDI treatment within the evaluation period.  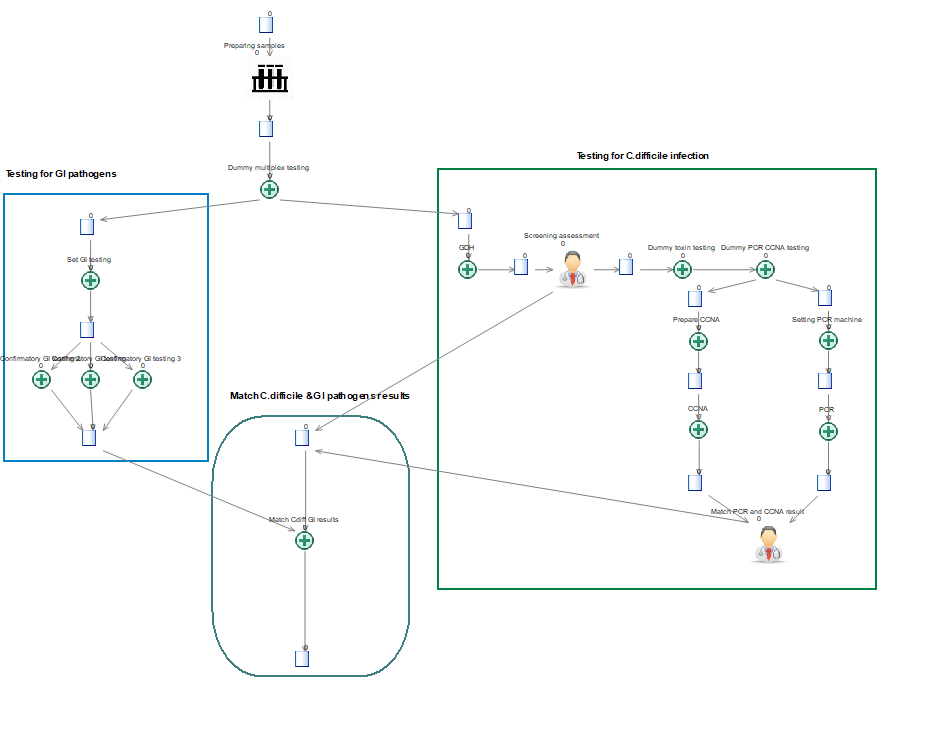  The activity **‘Multiplex GI panel’** performs the same activity as per the HT testing strategy arm.  The activity ‘**Match Cdiff GI results’** matches 2 samples being tested simultaneously with multiplex PCR and toxin POCT based on Patient ID and it estimates the Final Diagnosis TAT as the time difference between when stool sample is ready and when test results are back.  The activity **‘Dummy Post Testing’** links the individual test results to where the patient is staying in presumptive isolation be it Single Rooms or General Ward once final diagnosis is reached. Once a patient enters presumptive isolation (regardless of the location), a global variable will store their PatientID. Once test results are ready, this activity will check if the PatientID from the sample/entity being processed is equal to any of the PatientID global variables either in single rooms, general ward or awaiting to receive quick test results. Once PatientID label and PatientID global variables are matched, this activity will route out the entity accordingly  Example: Patient 6 has entered presumptive isolation in Single Room 5. Single Room 5 ID will be set to 6 (equal to Patient 6’s ID). Dummy Post Testing will check if the Patient ID label of the entity being processed is equal to Single Room 1 ID and then Single Room 2 ID and then Single Room 3 ID [..] until Single Room 5 ID. Since Patient ID label is equal to Single Room 5 ID, this activity will route out the entity (with test result info) towards Single Room 5 to match patient and test results.  This activity also determines clinical outcomes such as hospital LOS are based depending on how long it took to receive final diagnosis (source Barbut et al 2014 study) – only for patients who are truly CDI positive (lbl_true disease CDI = 1). Call **LOS Final diagnosis TAT VL** – this VL calculates how the timespan patient has already spent within the simulation and then deduct it from the total LOS which is dependent on the time-to-diagnosis. Length of stay are based on distributions informed by Barbut et al study which are then multiplied by 600 as they are originally set in days. Cut-off points (e.g. 300 and 720 are based on the study design) | |
| Scenario logic | 2.3 | As HT is a hypothetical test, no information is available currently on its diagnostic accuracy, turnaround time or test price. Various scenarios and sensitivity analyses were run in the model varying test turnaround time and diagnostic accuracy.  The analysis was designed to pragmatically and efficiently isolate the minimum acceptable performance specifications for each of the aforementioned outcomes of interest, and test the robustness of those results. The analysis therefore consists of two consecutive phases, including:   1. **Minimum performance specifications framework** – a novel three-stage framework (henceforth ‘MPS framework’) was developed to pragmatically identify the expected minimum performance benchmarks for the HT based on deterministic clinical- and cost-effectiveness outputs. All other model parameters are held at their baseline value at this stage. 2. **Sensitivity and scenario analyses** – deterministic univariate sensitivity and scenario analyses were conducted to assess the impact of varying each model parameter, and structural scenarios, on the clinical- and cost-effectiveness outputs of the model associated with the minimum performance specifications identified in phase 1. The aim of this phase was to identify key drivers of the model outputs which could potentially impact on the results from phase 1. Then, additional sensitivity analyses were conducted to assess the impact of the top two influential model parameters and structural scenarios on the minimum performance specifications determined in phase 1. Results from this phase indicate how the minimum performance requirements identified in phase 1 might change depending on different values of the model key drivers.   The sections below provide further information on the deterministic sensitivity analyses and scenario analyses, separately.  ***Deterministic univariate sensitivity analysis***  Deterministic univariate sensitivity analyses were conducted to explore the impact of varying each of the model parameters on the clinical- and cost-effectiveness outputs. These analyses were run assuming a 15-minute test turnaround time, fixing the diagnostic sensitivity and specificity values to the minimum performance specifications identified in phase 1, and fixing the test price at the threshold value.  For the remaining model parameters, lower and upper bounds for the parameter values were defined. Each sensitivity analysis was then run using 70 model replications (using different random number sequences), recording the incremental QALY gains, number of secondary cases, incremental costs, and INMB at £20,000 WTP per QALY gained. Full details on the lower and upper parameter values applied in this analysis are provided in Appendix 1. For several parameters, lower and upper bounds were taken from a recent economic model on CDI diagnostics (266), or from the literature, where available. For the remaining parameters, the range of parameter values assessed was based on a +/-25% and +/-50% deviation from the baseline parameter value, separately.  *Scenario analyses*  The clinical care pathway for patients suspected with CDI varies across different hospitals. Based on consultations with clinical experts, key structural variations in the care pathway for patients suspected with CDI were identified. Scenario analyses were therefore conducted in this phase to explore the impact of key structural differences on the modelled outputs. As before, these analyses were conducted assuming that sensitivity and specificity equal to the minimum performance specifications at 15-minutes test turnaround time as derived in phase 1, and fixing the test price at the threshold value. Full details on the scenarios explored are provided in Appendix 1. Scenarios investigated in this phase include:   1. **Alternative de-escalation protocol for patients confirmed with CDI** - at LTHT, patients confirmed with CDI remain in single room isolation until the end of their hospital stay. Based on consultations with clinical experts, clinicians in other hospitals de-isolate patients confirmed with CDI 48 hours after the resolution of symptoms. This difference in de-escalation practices might have an impact on the availability of single rooms. 2. **Increased availability of multiplex GI panels** – the quantity of multiplex GI molecular platforms available may vary across different clinical settings depending on laboratory’s financial constraints and number of stool samples being tested. This scenario explored the impact of having an additional multiplex GI molecular platform – resulting in a total of four multiplex GI molecular platforms being available to detect other GI pathogens in patients suspected with CDI. 3. **Reduced availability of single rooms** – this scenario simulated the impact of HT in a smaller hospital with reduced availability of single rooms compared to LTHT. The baseline value of single rooms available (n=93) was reduced by 25%, resulting in 70 available single rooms in this scenario. 4. **UK District Hospital** – compared to UK Teaching Hospitals, UK District Hospitals typically have: (i) fewer single rooms (93 single rooms within UK Teaching Hospital vs 64 single rooms within UK District Hospital); (ii) fewer patients being tested for CDI (1430 monthly average of stools being tested within UK Teaching Hospital vs 850 stools within UK District Hospital); and (iii) an off-site laboratory (see Appendix O for further details). This scenario analysis explored the impact of these differences. 5. **Application of second-best distribution to time-to-event parameters from parametric survival analysis** – this scenario investigated the impact of applying a different parametric distribution to the duration of symptoms and LOS parameters. For each time-to-event parameter, the second-best fitting distribution was selected. Key properties of the time-to-result parameters are summarised in Appendix O. 6. **Inclusion of labour costs for running laboratory testing** – this scenario investigated the impact of including additional labour costs for running laboratory-based testing strategies. Since HT is a ward-based POCT, additional costs are applied solely to the standard care testing strategy.   For each scenario analysis, 70 model replications were run recording the incremental QALY gains, number of secondary cases, incremental costs and INMB at £20,000 WTP per QALY gained. | |
| Algorithms | 2.4 | See Technical documentation - available upon request  This section explains the following simulation logics:   1. **Single Rooms and Cohort Bays Full Capacity Time VL**   This VL counts how many minutes both single rooms and cohort bays are in full capacity. It performs the following tasks:   - It checks whether single rooms are in full capacity (i.e. 93 single rooms are already occupied) - If single rooms are in full capacity, it checks if cohort bays are also in full capacity - If cohort bays are in full capacity, it checks if cohort bays were already in full capacity up to this point. If cohort bays have just started to be in full capacity now, it means that 'Time start full side room capacity' global variable was equal to 0. If that's the case, set this variable equal to Simulation time - In case cohort bays are no longer in full capacity, check if cohort bays were in full capacity up to this point. If cohort bays were in full capacity, global variable 'Time start full side room capacity' is supposed to be greater than 0 (as previously defined). Sum the total minutes single rooms and cohort bays were in full capacity within the result collection period. Since now cohort bays are no longer in full capacity, set global variable ‘Time start full side room capacity’ again equal to 0. Once single rooms & cohort bays will be in full capacity again, the simulation will set 'Time start full side room capacity' equal to Simulation time.  1. **Allocation Single Rooms VL**  - Check if there is already one patient in single room 1. If not, move the patient to single room 1 and set single room 1 Patient ID global variable equal to Patient ID label. This will help later to match the patient test result with the patient waiting in isolation (see Dummy Post Testing activity). If there is already one patient in single room 1, check current capacity of single room 2 (until reaching the last available single room).  1. **Check symptoms resolution (general) VL**  - This VL checks if patient is still symptomatic. Depending on the timespan a patient has already spent in the simulation and time to clear symptoms (individual value drawn from a distribution), define if a patient is still symptomatic or not. Depending on the test result for CDI there are different VL:   - CDI True-Positive patients (lbl_CDI Final diagnosis =1) – call **Check symptoms resolution CDI True Pos patients VL**     - Check if patient has already become asymptomatic following treatment regimen for CDI       - If yes, check symptoms resolution depending on how long patient has spent in simulation and lbl_time to clear symptoms         - If the patient is still symptomatic, check if they have experienced treatment failure for CDI   If yes, the patient has already had 4 additional days of CDI treatment. Assume that after 4 additional days of treatment, the patient fully recovers and becomes asymptomatic  If the patient has not experienced treatment failure (yet), check if CDI treatment has already been started   - - - - - If not, assign lbl_clinical symptoms = 1 patient will then start 10 days of treatment regimen.         - If the patient has already started CDI treatment regimen, check if the time elapse between time to start treatment and current Simulation time is equal or greater than 10 days (6000 SIMUL8 minutes). NB – treatment regimen for CDI lasts at least 10 days.   If not, this means that the patient has not completed the 10 days of treatment regimens. Assign lbl_clinical symptoms = 1 so that the simulation will check once a day if symptoms have resolved – set time to next check equal to 600 min (1 day)  If the patient has completed 10 days of CDI treatment regimen, assign a probability of clinical cure depending on how long it took to receive final diagnosis for CDI.   - - - If lbl_clinical symptoms = 2 🡪 treatment cure, the patient is no longer symptomatic     - If lbl_clinical symptoms = 1 🡪 clinical failure, the patient is still symptomatic and need to receive 4 additional days of CDI treatment - CDI False-Positive patients (lbl_CDI Final diagnosis = 4) – call **Check symptoms resolution CDI False Pos patients VL** - Check if patient has already become asymptomatic following treatment regimen for CDI   - If yes, check symptoms resolution depending on how long patient has spent in simulation and lbl_time to clear symptoms - If the patient is still symptomatic, check if they have experienced treatment failure for CDI   - If yes, the patient has already had 4 additional days of CDI treatment. Assume that after 4 additional days of treatment, the patient fully recovers and becomes asymptomatic - If the patient has not experienced treatment failure (yet), check if CDI treatment has already been started   - If not, assign lbl_clinical symptoms = 1 patient will then start 10 days of treatment regimen. - If the patient has already started CDI treatment regimen, check if the time elapse between time to start treatment and current Simulation time is equal or greater than 10 days (6000 SIMUL8 minutes).   - If not, this means that the patient has not completed the 10 days of treatment regimens. Assign lbl_clinical symptoms = 1 so that the simulation will check once a day if symptoms have resolved – set time to next check equal to 600 min (1 day) - If the patient has completed 10 days of CDI treatment regimen, assume that patient remains symptomatic. False-positive patients receive inappropriate antibiotic treatment, so they are not cured for the pathogen/causing their diarrhoea. Assume they remain symptomatic at day 10 of treatment regimen and receive treatment for 4 additional days. - CDI Negative patients (lbl_CDI Final diagnosis = 2 or 3) – call **Check symptoms resolution CDI Neg patients VL**. Check symptoms clearance depending on how long patient has already spent in the simulation and time to clear symptoms.   - - Depending on the symptoms resolution, take a clinical management decision depending on the test results     - SYMPTOMS PERSIST (lbl_clinical symptoms =1) – call **Check test results and symptoms (rapid diagnosis) VL**. CDI positive patients will remain in isolation, whereas CDI negative and GI negative patients will be moved into General ward. GI positive patients are set daily checks for symptoms clearance and will remain in isolation  1. **Cohort Bay Re Allocate CDI POS (Setting Data Cohort Bay) VL**  - The model will check capacity Cohort Bay 1. If Cohort Bay 1 has more than 5 patients, then check capacity Cohort Bay 2. If the latter has more than 4 patients, move patient into General Ward - GI positive patients – call Cohort Bay Re Allocate GI Pos (Setting Data Cohort Bay) VL - The model will check capacity Cohort Bay 3. If Cohort Bay 3 has more than 5 patients, then check capacity Cohort Bay 4. If the latter has more than 4 patients, move patient into General Ward - For both patient subgroups, set time to next check (isolation) equal to half a day. Every half a day the simulation will check if patients in Cohort Bays can be moved into single rooms isolation if there are any single rooms being available at the moment.   1. This VL also calculates how many patients within the evaluation period are moved into isolation and how many are isolated within Cohort Bays | |
| Components | 2.5 | 2.5.1. Entities | This model tracks in-hospital adult patients with an initial episode of acute diarrhoea for whom clinicians have requested stool testing for CDI. |
|  |  | 2.5.2. Activities | The key activities for both models are presented in the **Figure 1** below and described in section 2.1 and 2.1.  Certain activities (i.e. ‘Stool sampling’, ‘HT’, ‘Allocate isolation’, ‘CCNA’) were replicated for many times (i.e. 1,000) to ensure that there are no bottlenecks in the model.  **Figure 1**  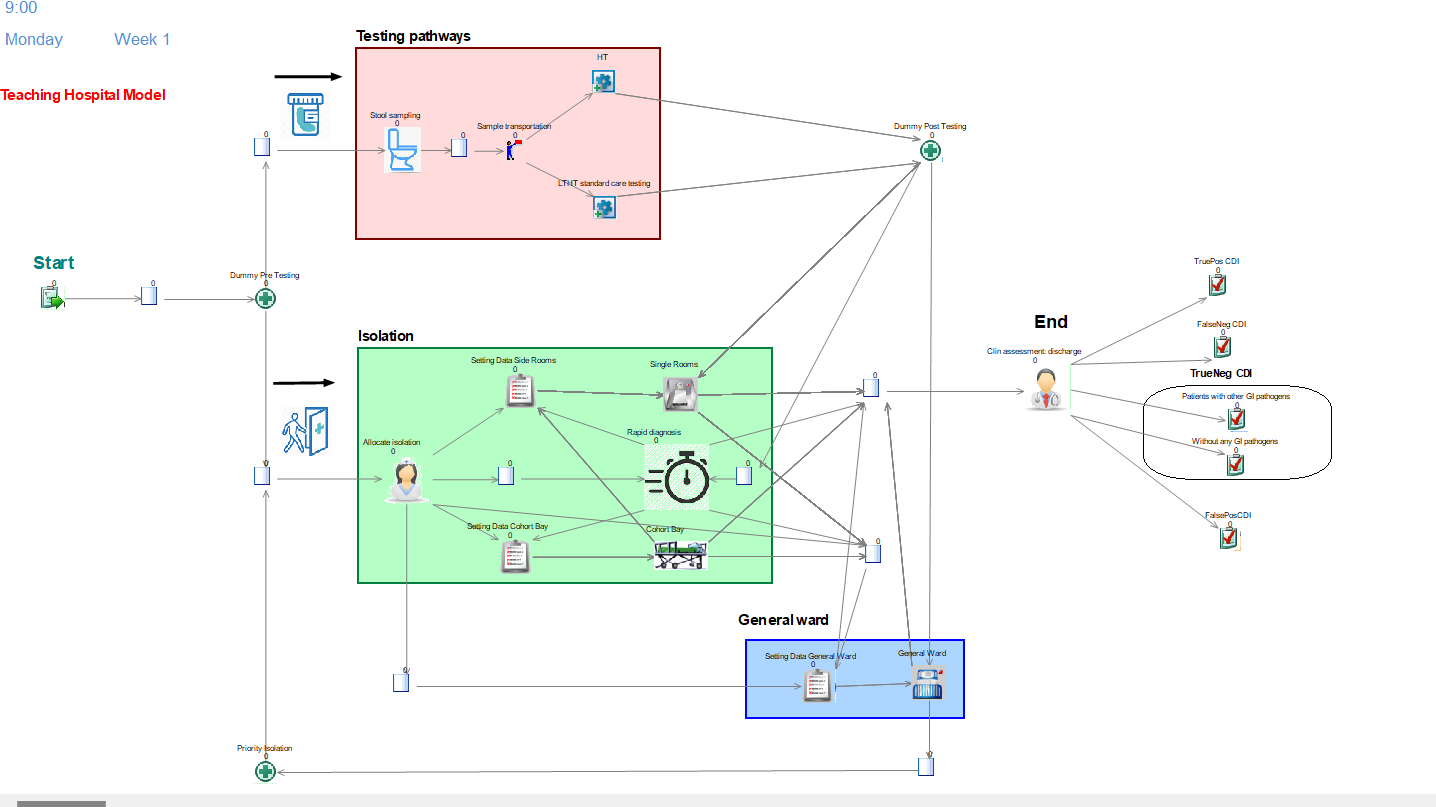 |
|  |  | 2.5.3. Resources | The activities ‘Preparing samples’, ‘GDH’, ‘Screening assessment’ related to the standard care testing strategy accept new samples at 10am and 4pm when the resources ‘Lab worker’ and ‘Biomedical scientist’ are released.. At LTHT an on-site laboratory routinely processes stool samples at 10am and 4pm. Samples arriving before these time points will wait before being processed.  No resources were applied to the other activities within the model. |
|  |  | 2.5.4. Queues | All queues in the models follow a first in first out discipline, except the ‘Queue for Allocate Isolation’ which prioritises patients depending on lbl_priority label (0 = no priority for patients suspected with infectious diarrhoea once they enter the model, 1 = priority for patients who have been confirmed as having infectious diarrhoea in the general ward). |
|  |  | 2.5.5. Entry/Exit points | Entry and Exit points for both models are presented in **Figure 1**.  All patients enter the model at the ‘Start Point’. The speed at which new patients enter the model was dictated by an assigned inter-arrival time. In the model, the monthly average of stool samples tested for CDI only equates a mean inter-arrival time of 35 minutes (meaning that a new symptomatic patient enters the model on average every 35 minutes), assuming an exponential distribution. Across both testing strategies, all patients exit the model to different ending point depending on their CDI diagnosis. Patients receiving a true-positive diagnosis for CDI exit the model at ‘TruePos CDI’, whereas those receiving a false-negative diagnosis exit the model at ‘FalseNeg CDI’. Patients receiving a true-negative diagnosis for CDI are sorted depending on the presence of other GI pathogens in their stools: patients infected with other GI pathogens exit the model in ‘Patients with other GI pathogens’, whereas those without exit the model in ‘Patients without any GI pathogens. Patients receiving a false-positive diagnosis for CDI exit the model at ‘FalsePos CDI’. |
| 1. **Data** |  | | |
| Data sources | 3.1 | A list of input parameters and associated data sources can be found in Appendix 1.  To populate the model, we used the main data sources, including:   - **COMBACTE-CDI study datasets** – to inform patient characteristics and hospital configurations. In particular, UK-individual patient data (IPD) from the COMBACTE-CDI case report form (CRF) dataset (n= 180 patients from 23 UK sites) was used to inform model parameters relating to patient characteristics (e.g. disease severity, symptoms duration, hospital LOS). In addition, UK-specific summary data from a European-wide survey part of COMBACTE-CDI study was used to inform key model parameters related to hospital configuration (e.g. numbers of samples run and single rooms available). This survey was sent out to community and hospital sites (n=158) across 12 European countries to assess current clinical practices for CDI patients and CDI costs (289). - **NHS Reference Costs, British National Formulary** – to inform unit costs - **Published literature** – to inform features of the LTHT testing pathway, reproductive rate of infection spread, health-related utility weights for CDI and costing estimates | |
| Pre-processing | 3.2 | Parametric survival analysis was conducted to determine appropriate distributions for time-to-event data in the model (i.e. duration of symptoms for CDI positive and negative patients, and hospital LOS). For each of these variables, different parametric models (Normal, Lognormal, Exponential, Gamma and Weibull) were fitted to the UK-based COMBACTE CDI IPD data using the R ‘fitdistrplus’ package (288). Based on a maximum likelihood estimation (MLE) process, the optimal fitting distribution for each variable was chosen based on: (i) an analysis of the Akaike Information Criterion (AIC) and Bayesian Information Criterion (BIC) metrics (the distribution with the lowest AIC and BIC values indicating the parameterisation with the optimal statistical fit to the data); (ii) an analysis of key summary statistics produced from the various parameterisations (i.e. mean, median, inter-quartile range, standard deviation [SD], and maximum values); and (iii) visual inspection of the goodness of fit of the different parametric models against the observed patient data (288).  The inter-arrival time was set equal to the expected frequency at which patients develop diarrhoeic symptoms within the hospital setting. This estimate was based on the average number of stool samples tested in the laboratory over a month from the COMBACTE-CDI. The observations from this data were highly right-skewed, with the mean being greater than the median due to a small number of high outliers in the data (n=2). Based on consultations with a clinical expert, the median number of samples being tested (for any condition) was considered to better represent the workload within a typical UK Teaching Hospital. This estimate (n= 1430) was thus applied in the model.  This data, however, included stool samples being tested for other conditions, not only CDI. In addition to the above data, the COMBACTE-CDI survey included a question about the proportion of stool samples being tested only for CDI. Respondents were asked to select from a multiple-choice question about the proportion of samples being tested for CDI only on a monthly basis (including: ‘less than 30% of samples’, ‘between 30-49%’, ‘between 50-69%’, ‘between 70-99% or ‘100%’ of samples’). Because of the wording of the question, the frequency of the specific values within each range was unknown. As such, within each range, a uniform distribution was applied so that each value within the range was assumed to be equally likely to occur. In addition, for the first range (e.g. 0-30%) a minimum proportion of samples being tested for CDI was set equal to 5% to ensure functionality of the simulation. Out of 8 participating UK sites in the COMBACTE-CDI survey, the majority reported testing between 30%-49% samples for CDI (50%, n=4) or, alternatively, less than 30% of samples (25%, n=2). The remaining UK sites would test between 50%-69% of samples for CDI only (25%, n=2). This information was combined with the median number of samples to calculate the number of monthly samples tested for CDI in the model.  The following steps were taken to calculate the inter-arrival time, including:   1. Calculate the proportion of samples being tested for CDI only  - Set global variable X1 equal to dist_proportions of samples tested only for CDI   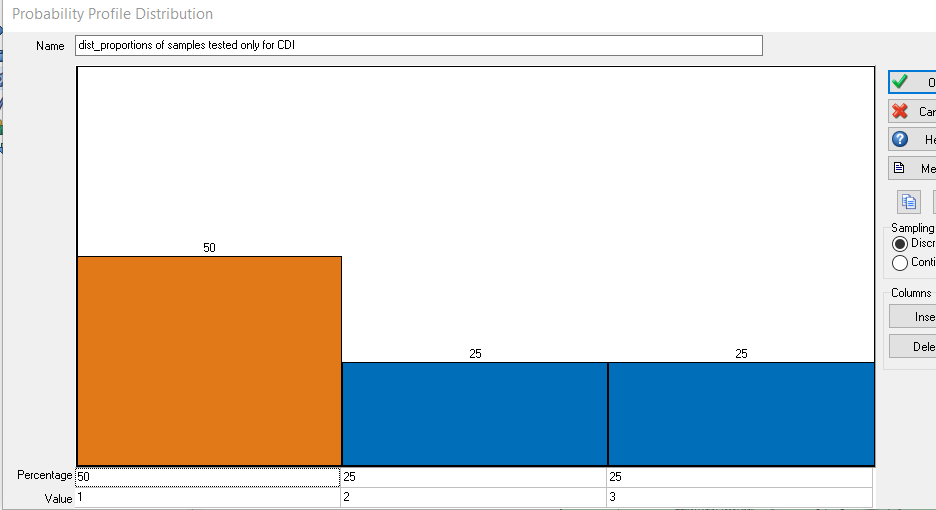   - - Value 1: 30-49% of samples being tested for CDI only   - Value 2: less than 30% of samples being tested for CDI only   - Value 3: 50-69% of samples being tested for CDI only - Assume an equal probability within each range (e.g. if you consider value 2, 0-10%, 10-20%, 20-30% ranges have a probability of 0.33). This is because COMBACTE-CDI survey data does not report the frequency within each range. For each range (e.g. 1-2-3), assign an uniform distribution.   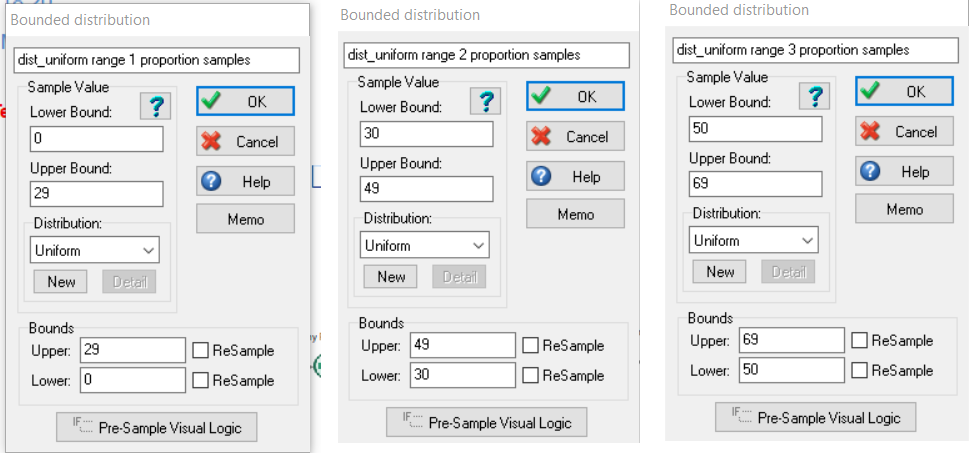  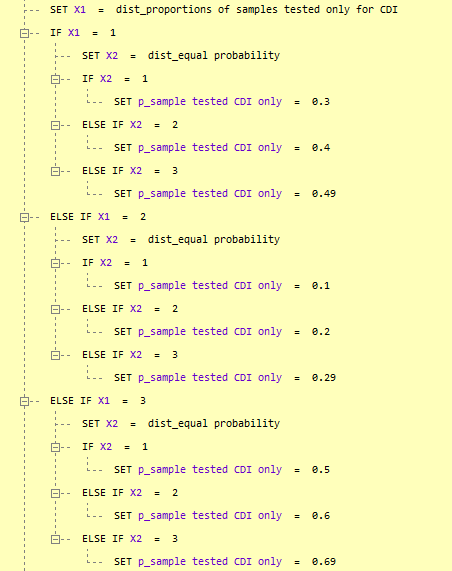  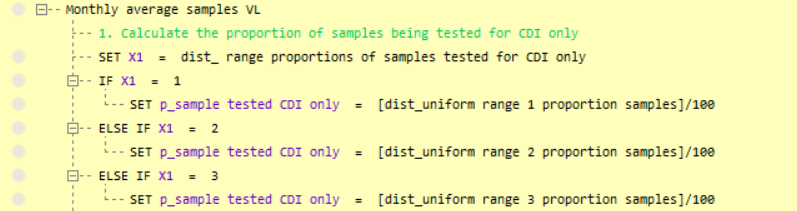   1. Calculate monthly average of samples tested only for CDI  - Depending on the hospital size, multiply the median stools being tested with the proportion of samples being tested only for CDI to monthly average samples - Calculate weekly average= monthly average CDI only/4 - Calculate daily average= weekly average/5 working days - Calculate number of samples per minute= daily average/600 min^[[3]](#footnote-3)^ - Lambda= 1/number of samples per minute - Plug lambda in an exponential distribution   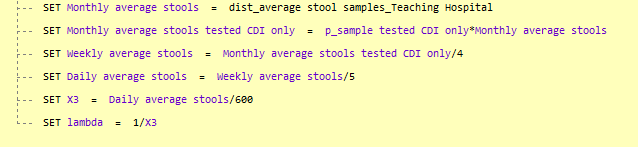   - Lambda = 1/ number of samples per minute  1. Set Start Point inter-arrival time equal to an exponential distribution with lambda as average   The monthly number of stool samples tested for CDI only was approximately equal to 539. As the monthly demand for stool testing was assumed to be constant and independent from seasonality or other external factors, an exponential distribution was selected to calculate the inter-arrival time. | |
| Input parameters | 3.3 | A list of model input parameters for both models is provided in Table 1 | |
| Assumptions | 3.4 | Key clinical assumptions (CA) underpinning the model are summarised below. These assumptions were informed by a series of consultations with clinical experts (including a Principal Clinical Scientist and a Clinical Research Fellow at LTHT).   1. The monthly demand for stool testing was assumed to be constant and independent from seasonal change or other external factors. 2. There is no risk of co-infection between GI pathogens and CDI. 3. Isolation in single rooms is required for patients positive to other GI pathogens. 4. When single rooms and cohort bays are at full capacity, patients confirmed with infectious diarrhoea remain in general ward until hospital discharge. 5. A rapid multiplex GI panel with assumed perfected diagnostic accuracy is run to detect multiple pathogens – separate to tests for CDI. Perfected diagnostic accuracy was assumed for the multiplex GI panel as the focus of this decision model lies in evaluating testing strategies for CDI, rather than other GI pathogens. 6. Clinicians consider the continuation or resolution of diarrhoea as the only symptom for CDI – without assessing whether the frequency of stools is improving or worsening. 7. Clinicians are assumed to pre-emptively administer treatment for CDI (i.e. ‘empirical treatment’) prior to receipt of test results if a patient presents with severe symptoms of CDI – as per current clinical practice at LTHT. 8. Clinicians fully adhere to test results when deciding when to start or stop administering antibiotic treatment for CDI. 9. Early treatment, via early diagnosis, is assumed to have no impact on patient survival, risk of disease recurrence and long-term quality of life due to paucity of data. 10. Patients with a true-positive (TP) test result for CDI are assumed to recover to full health without risk of disease recurrence at the end of the antibiotic treatment regimen. 11. Patients with a FN test result for CDI do not experience further disease recurrences. 12. Patients with a FP test result for CDI are assumed to remain symptomatic at day 10 of treatment and receive treatment for 4 additional days – as per current clinical practice at LTHT. | |
| 1. **Experimentation** |  | | |
| Initialisation | 4.1 | A warm-up period of 45 days (i.e. nine weeks) was applied in the model, to appropriately capture ongoing capacity constraints. The selected warm-up period was set to values sufficient to provide stable outputs for the number of secondary infections within the general ward. The model runs in minutes. | |
| Run length | 4.2 | Upon completion of the warm-up period, each model replication runs for a time horizon of 7 months (i.e. the *results collection period*). The model records outputs for every patient that enters the hospital during the *model entry period*, which is set equal to 60-days (two months) following the end of the warm-up period. Extending the results collection period to 7 months ensures that every patient within the model *evaluation set* (i.e. those entering within the model entry period) can have their full experience of the clinical pathway simulated. Whilst clinical management of CDI patients within the hospital usually lasts approximately up to one month (260, 266), a proportion of patients experience extended hospital stays (as reflected in the right-skewed tails of the LOS distributions applied in the model). A seven-month extended results collection period was therefore required to ensure outputs for patients entering during the model entry period were captured. Whilst additional patients were allowed to enter the model after the model entry period (to continue to simulate the busy hospital environment), only results for those patients who entered the model during the model entry period were recorded.  Figure below provides a schematic of the timeline for the model analysis.  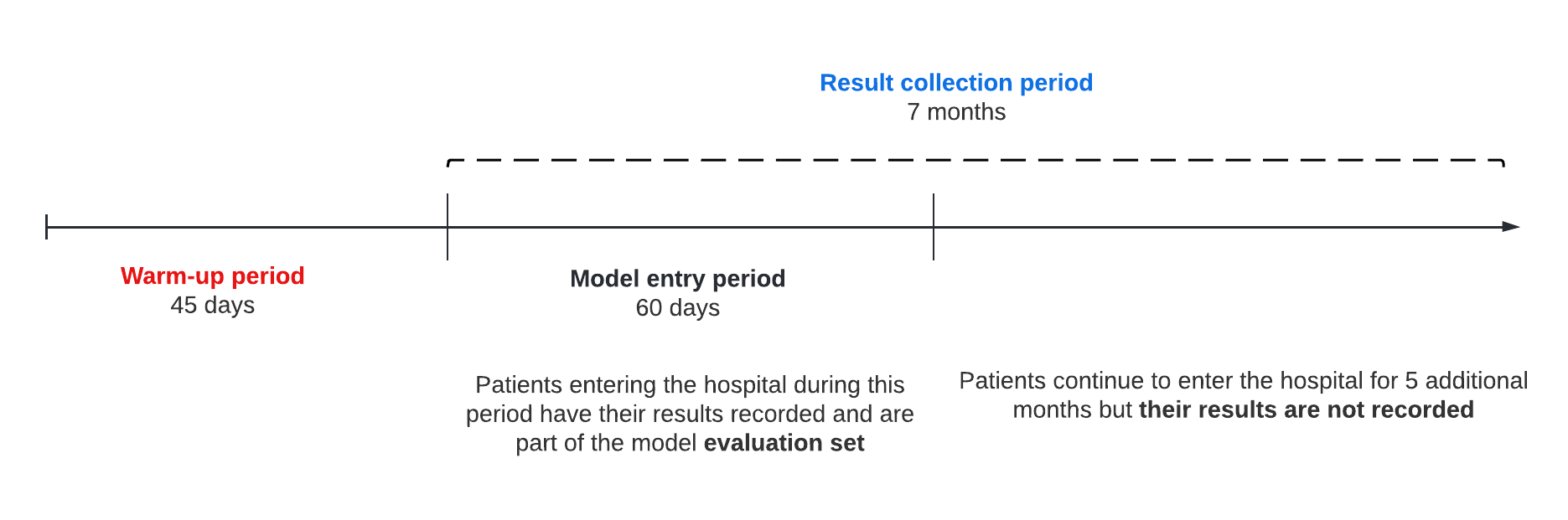 | |
| Estimation approach | 4.3 | All model analyses were based on running 70 *model replications* (i.e. running the model 70 times, with each run using a different random number sequence). This approach ensures that the impact of *first-order* uncertainty on the model results is appropriately accounted for. The replication number was set to values sufficient to provide stable outputs for the number of secondary infections within the general ward. | |
| 1. **Implementation** |  | | |
| Software or programming language | 5.1 | The models were developed in Simul8 (SIMUL8 Corp, Boston, MA)([https://www.simul8.com](https://www.simul8.comD)). | |
| Random sampling | 5.2 | All model analyses were based on running 650 replications of the model, to account for first order uncertainty – each run using a different random number sequence. | |
| Model execution | 5.3 |  | |
| System Specification | 5.4 | The model was implemented in the commercial software SIMUL8 version 28.0.0.4060 Student edition, and run on a VivoBook ASUS laptop X571GT_F571GT with a 2.60 GDHz Intel ® Core ™ i7 processor and 16 GB of memory under Microsoft Windows 10 Home (build 19,043). Total model run time was 20 minutes for HT testing strategy and 40 minutes for LTHT testing strategy. The model runs from 9am to 7pm (10 hours = 600 minutes in SIMUL8) for 5 days each week (Monday to Friday). Simulated time within the model progresses according to the *next time-to-event progression* mechanism. | |
| 1. **Code Access** |  | | |
| Computer Model Sharing Statement | 6.1 | Simul8 software can be purchased via the Simul8 website: [https://www.simul8.com](https://www.simul8.comD). Model coding is available upon request to Paola Cocco (P.Cocco@leeds.ac.uk) | |

1. This programme grant is called *‘*Rapid infections diagnostics to combat antimicrobial resistance’ (RID-AMR@Leeds) research group (89). [↑](#footnote-ref-1)
2. If a patient remains longer in single room isolation than the assigned LOS due to slow time-to-diagnosis, during the next check to assess resolution of symptoms, the model has the capacity to assess if the time the patient has spent within the simulation exceeds the assigned individual LOS. If so, the patient is assumed to be discharged. [↑](#footnote-ref-2)
3. The simulation runs from 9am to 7pm (10 hours) on Monday-Friday. Each day has 60*10= 600 minutes [↑](#footnote-ref-3)
