# Supplementary material for: Early Economic Modeling to Inform a Target Product Profile: A Case Study of a Novel Rapid Test for Clostridioides difficile Infection
Source: MDM Policy Pract. 2024 Nov 22;9(2):23814683241293739. doi: 10.1177/23814683241293739 (PMC11585019; doi:10.1177/23814683241293739)
Supplement: sj-docx-3-mpp-10.1177_23814683241293739 – Supplemental material for Early Economic Modeling to Inform a Target Product Profile: A Case Study of a Novel Rapid Test for Clostridioides difficile Infection [file sj-docx-3-mpp-10.1177_23814683241293739.docx]

# **Appendix 3**

This Appendix includes additional information on (i) model analysis ; and (ii) model results.

## **Model Analysis**

- - 1. **Model initialisation**

**Supplementary Figure 3. 1 Assessment of how long the warm-up period should be to stabilise the number of new secondary cases in general ward – HT testing strategy at 15 minutes turnaround time**

**Supplementary Figure 3. 2 Assessment of how long the warm-up period should be to stabilise the number of new secondary cases in general ward – LTHT testing strategy**

**Model replication estimation**

**Supplementary Figure 3. 3 Assessment of how many model replications required to stabilise the number of new secondary cases in the general ward – HT testing strategy at 15-minutes turnaround time**

**Supplementary Figure 3. 4 Assessment of how many model replications required to stabilise the number of new secondary cases in the general ward – LTHT testing strategy**

### **Phase 2: identification of key model drivers**

For parameters where IPD were available, a decrease or increase of 25% from baseline IPD values was applied and then the scale and shape parameters for the fitted Weibull distributions were re-calculated to estimate the lower and upper bound.

For Weibull parameters based on aggregate data from the Barbut, F. et al. (260) study, the scale and shape parameters were re-calculated by applying a +/- 25% change to the mean estimates, holding the standard deviation constant.

**Supplementary Table 3. 1 Lower and upper bounds for each model parameter, alongside calculations and sources, used to estimate the ranges for the univariate sensitivity analysis**

| **Parameter** | **Lower bound** | **Upper bound** | **Sources and calculations** |
| --- | --- | --- | --- |
| Disease prevalence CDI | 0% | 25% | Jones, W.S. et al. (267) |
| Disease prevalence other GI pathogens | 0% | 25% | Jones, W.S. et al. (267) |
| LOS CDI negative patients (days) | Shape: 0.8790504  Scale: 19.9177571 | Shape: 0.8790134  Scale: 33.2105691 | COMBACTE-CDI CRF dataset – uplift or lower IPD baseline value by 25% and re-calculate shape and scale parameters for Weibull distribution |
| LOS CDI positive patients – slow diagnosis (days) | Shape: 0.6491104  Scale: 16.60751 | Shape: 1.043657  Scale: 38.52885 | Barbut, F. et al. (260)– uplift or lower baseline value by 25% and re-calculate shape and scale parameters for Weibull distribution |
| LOS CDI positive patients – average diagnosis (days) | Shape: 0.7128001  Scale: 16.2108 | Shape: 1.167139  Scale: 35.4809 | Barbut, F. et al. (260) – uplift or lower baseline value by 25% and re-calculate shape and scale parameters for Weibull distribution |
| LOS CDI positive patients – rapid diagnosis (days) | Shape: 0.7011098  Scale: 13.76674 | Shape: 1.144493  Scale: 30.42887 | Barbut, F. et al. (260) – uplift or lower baseline value by 25% and re-calculate shape and scale parameters for Weibull distribution |
| Duration diarrhoea after sampling – CDI negative patients (days) | Shape: 0.5542175  Scale: 3.175754 | Shape: 0.55433  Scale: 5.294004 | COMBACTE-CDI CRF dataset – uplift or lower IPD baseline value by 25% and re-calculate shape and scale parameters for Weibull distribution |
| Duration diarrhoea after sampling – CDI positive patients (days) | Shape: 0.5879807  Scale: 5.610213 | Shape: 0.5879725  Scale: 9.344171 | COMBACTE-CDI CRF dataset – uplift or lower IPD baseline value by 25% and re-calculate shape and scale parameters for Weibull distribution |
| Reproductive ratio for CDI | 0.55 | 1.99 | Lanzas, C. et al. (293) |
| Utility weight for adult UK inpatient with the first episode of CDI | 32% | 52% | Wilcox et al calculations of the low and upper bound by using the z value for 95% CI |
| Probability of clinical cure – slow diagnosis | 63.975% | 100% | Barbut, F. et al. (260) – decrease baseline value by 25% to estimate lower bound; set upper bound equal to 100% |
| Probability of clinical cure – average diagnosis | 68.025% | 100% | Barbut, F. et al. (260) – decrease baseline value by 25% to estimate lower bound; set upper bound equal to 100% |
| Probability of clinical cure – rapid diagnosis | 71.7% | 100% | Barbut, F. et al. (260) – decrease baseline value by 25% to estimate lower bound; set upper bound equal to 100% |
| GDH EIA diagnostic sensitivity | 94% | 96% | Crobach, M.J.T. et al. (222) |
| GDH EIA diagnostic specificity | 94% | 95% | Crobach, M.J.T. et al. (222) |
| PCR diagnostic sensitivity | 92% | 97% | Crobach, M.J.T. et al. (222) |
| PCR diagnostic specificity | 97% | 99% | Crobach, M.J.T. et al. (222) |
| CCNA diagnostic sensitivity | 75.8% | 100% | Planche, T. and M. Wilcox (294) – lower bound based on Barbut, F. et al. (309) estimate; upper bound set equal to perfect diagnostic sensitivity |
| CCNA diagnostic specificity | 96.7% | 100% | Planche, T. and M. Wilcox (294) – lower bound based on DiPersio, J.R. *et al.* (370) estimate; upper bound set equal to perfect diagnostic specificity |
| Time-to-obtain stool sample | 0 day | 2 days | Jones, W.S. et al. (267) |
| Time-to-transport sample to the laboratory | 0 minutes | 300 minutes | Assumption – high value is half-a-day as it requires transporting samples to an off-site laboratory |
| GDH EIA time-to-result | 45 minutes | 180 minutes | +/- 25% |
| PCR time-to-result | 30 minutes | 120 minutes | +/- 25% |
| CCNA time-to-result | 300 minutes | 3 days | Assumption |
| Multiplex GI panel operating time | 30 minutes | 50 minutes | Assumption |
| Cost of bed day in adult isolation | £519.6225 | £866.0375 | +/- 25% |
| Cost of bed day in general ward | £437.25 | £728.75 | +/- 25% |
| GDH EIA cost per kit | £3.63 | £6.05 | +/- 25% |
| PCR cost per run | £20.175 | £33.625 | +/- 25% |
| CCNA cost | £2.805 | £4.675 | +/- 25% |
| Multiplex GI panel cost per sample | £32.2725 | £53.7875 | +/- 25% |
| Additional cost per secondary case per day | £717.885 | £1196.475 | +/- 25% |
| Vancomycin cost | £99.3675 | £165.6125 | +/- 25% |

Scenarios investigated in this phase include:

1. **Alternative de-escalation protocol for patients confirmed with CDI** - at LTHT, patients confirmed with CDI remain in single room isolation until the end of their hospital stay. Clinicians in other hospitals de-isolate patients confirmed with CDI 48 hours after the resolution of symptoms. This difference in de-escalation practices might have an impact on the availability of single rooms.
2. **Increased availability of multiplex GI panels** – the quantity of multiplex GI molecular platforms available may vary across different clinical settings depending on laboratory’s financial constraints and number of stool samples being tested. This scenario explored the impact of having an additional multiplex GI molecular platform – resulting in a total of four multiplex GI molecular platforms being available to detect other GI pathogens in patients suspected with CDI.
3. **Reduced availability of single rooms** – this scenario simulated the impact of HT in a smaller hospital with reduced availability of single rooms compared to LTHT. The baseline value of single rooms available (n=93) was reduced by 25%, resulting in 70 available single rooms in this scenario.
4. **UK District Hospital** – compared to UK Teaching Hospitals, UK District Hospitals typically have: (i) fewer single rooms (93 single rooms within UK Teaching Hospital vs 64 single rooms within UK District Hospital); (ii) fewer patients being tested for CDI (1430 monthly average of stools being tested within UK Teaching Hospital vs 850 stools within UK District Hospital); and (iii) an off-site laboratory (see **Supplementary Table 3. 2** for further details). This scenario analysis explored the impact of these differences.
5. **Application of second-best distribution to time-to-event parameters from parametric survival analysis** – this scenario investigated the impact of applying a different parametric distribution to the duration of symptoms and LOS parameters. For each time-to-event parameter, the second-best fitting distribution was selected. Key properties of the time-to-result parameters are summarised in **Supplementary Table 3. 3**.
6. **Inclusion of labour costs for running laboratory testing** – this scenario investigated the impact of including additional labour costs for running laboratory-based testing strategies. Since HT is a ward-based POCT, additional costs are applied solely to the standard care testing strategy. See **Supplementary Table 3. 4** for further detail.

**Supplementary Table 3. 2 Summary of the key structural assumptions underpinning UK Teaching Hospitals and UK District Hospitals as simulated in the baseline scenario and S.4 scenario, respectively**

| **Feature of clinical setting** | **UK Teaching Hospital** | **UK District Hospital** | **Calculations** | **Source** |
| --- | --- | --- | --- | --- |
| Single rooms being available (n) | 279 | 192 | Assumption – one-third of the reported single rooms in the COMBACTE CDI survey would be allocated for patients suspected with diarrhoea. | COMBACTE-CDI survey |
| Single rooms simulated (n) | 93 | 64 |  |  |
| Monthly average of stools being tested – median (n) | 1430 | 850 |  | COMBACTE-CDI survey |
| Laboratory | On-site (15 minutes required to transport samples from ward to the laboratory) | Off-site ( two shifts: one in the morning and one at lunch time) | NA | Expert opinion |

**Supplementary Table 3. 3 Second-best fitting distribution and related parameters for each of the time-to-event parameters**

| **Time-to-event parameter** | **Distribution used at baseline** | **Second-best fitting distribution** | **Parameters for second-best fitting distributions** |
| --- | --- | --- | --- |
| S.6 (A) – LOS patients negative to CDI | Weibull | Exponential | Rate = 0.03496115  Mean = 28.6031781 |
| S.6 (B) – LOS patients positive to CDI (slow time-to-diagnosis) | Weibull | Exponential | Rate = 0.0330033  Mean = 30.3000003 |
| S.6 (C) – LOS patients positive to CDI (average time-to-diagnosis) | Weibull | Exponential | Rate = 0.03717472  Mean = 26.90000086 |
| S.6 (D) – LOS patients positive to CDI (rapid time-to-diagnosis) | Weibull | Exponential | Rate = 0.04310345  Mean = 23.19999907 |
| S.6 (E) – Duration of symptoms patients negative to CDI | Weibull | Gamma | Alpha = 0.41780903  Beta = 0.05934788 |
| S.6 (F) – Duration of symptoms patients positive to CDI | Weibull | Gamma | Alpha = 0.4486443  Beta = 0.0365449 |

**Supplementary Table 3. 4 Details on the additional labour costs for each test part of the standard care strategy applied to scenario 6**

| **Testing strategy** | **Additional labour costs** | **Total costing estimate (including labour costs)** | **Source** | **Notes** |
| --- | --- | --- | --- | --- |
| **GDH EIA** | £5.66 | £10.5 | (267) | Original estimate was inflated from 2013 to 2021 |
| **PCR** | £1.19 | £28.09 | (300) | Original estimate was inflated from 2011 to 2021 |
| **CCNA** | £4.78 | £8.52 | (300) | Original estimate was inflated from 2011 to 2021 |
| **Multiplex GI panel** | £1.19 | £44.22 | (300) | Original estimate was inflated from 2011 to 2021 |

## **Model results**

### **Phase 1: MPS framework**

Under the minimum performance specifications at a 15-minute test turnaround time, HT was clinically more effective compared to the LTHT testing strategy, leading to 0.0205 total incremental QALY gains, despite yielding 3 additional secondary cases of CDI in the general ward^[[1]](#footnote-1)^. Applying the threshold testing unit cost (i.e. £44.39), HT was less expensive compared to the LTHT testing strategy, leading to an increase in total costs of £410. This, in turn, led to an INMB of £0 per QALY gained at a WTP threshold of £20,000 per QALY gained. **Supplementary Table 3. 5** summarises the incremental clinical- and cost-effectiveness outputs associated with the minimum performance specifications for HT compared to LTHT testing strategy at 15-minutes turnaround time.

**Supplementary Table 3. 5 New secondary cases of CDI, total and per patient QALY gains , total and per patient costs at baseline for each testing option being evaluated, and incremental differences between HT meeting the minimum performance specifications at 15-minutes turnaround time and LTHT testing strategy**

|  | **HT – minimum performance specifications at 15-minutes turnaround time** | **LTHT testing strategy** | **Incremental difference**  **(HT – LTHT testing strategy)** |
| --- | --- | --- | --- |
| **New secondary cases of CDI** | 43.79 | 40.87 | 3 |
| **Total QALY gains** | 47.62755 | 47.60704 | 0.0205 |
| **Per patient QALY gains^[[2]](#footnote-2)^** | 0.08836 | 0.08832 | 0.00004 |
| **Total costs** | £17,574,907 | £17,574,496 | £410 |
| **Per patient costs^21^** | £32,607 | £32,606 | £1 |
| **INMB at £20,000 QALY gained** | £0 | | |

### **Phase 2: Identification of key model drivers**

**Supplementary Figure 3. 5** **Tornado plot showing the results of the deterministic univariate sensitivity analysis for the most influential model parameters using estimates from the literature and the +/- 25% deviation from baseline values. Results are expressed in terms of INMB at £20,0000 per QALY gained. INMB at baseline is equal to £0 and reflects the cost-effectiveness output associated with the minimum performance specifications for HT at 15-minutes turnaround time**


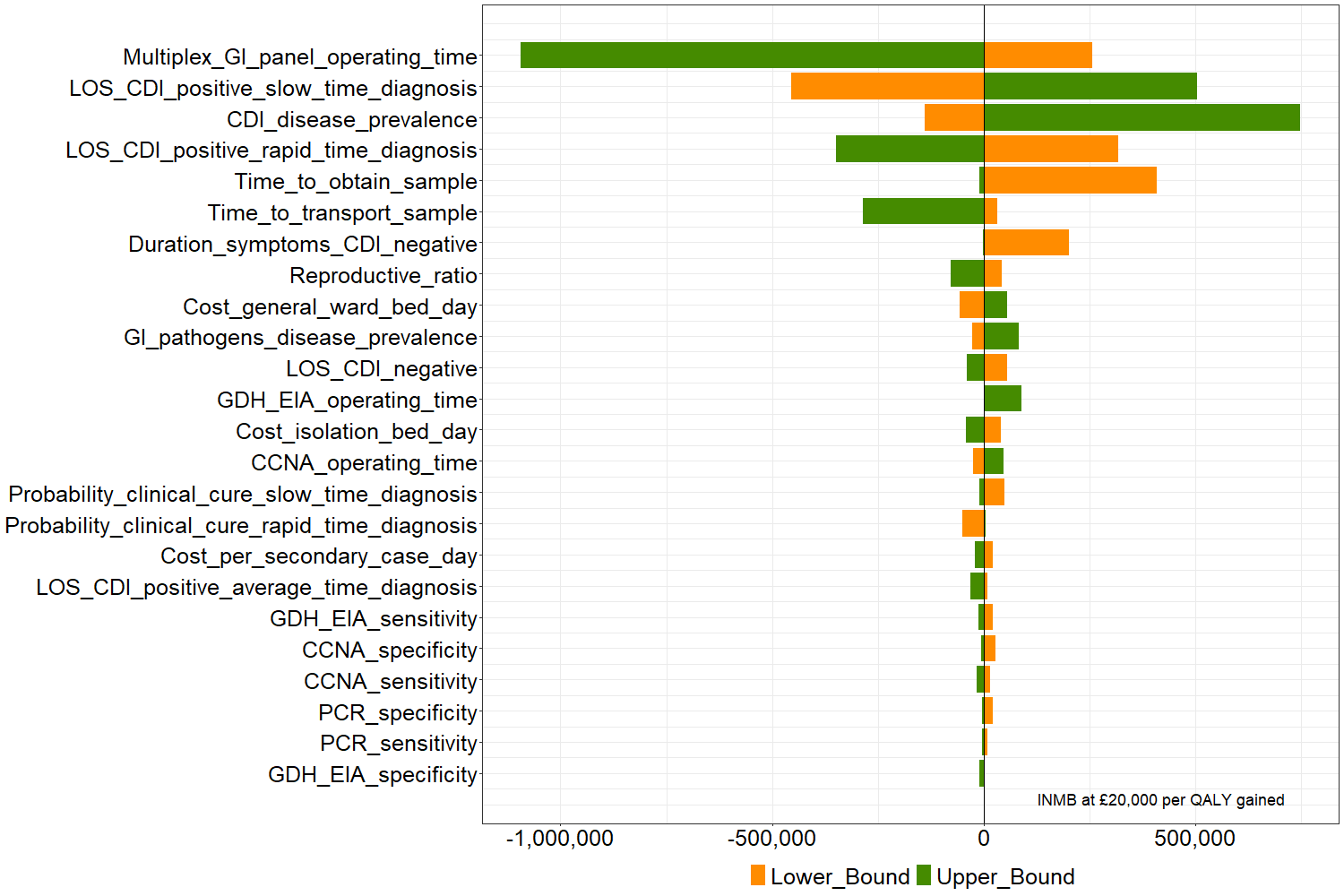


For analysis purposes, parameters were divided into the following clusters:

- **disease-related parameters** – parameters relating to patient characteristics (e.g. prevalence of CDI and other GI pathogens), duration of symptoms, hospital LOS, treatment effectiveness at day 10 of antibiotic treatment, reproductive ratio, and health-related utility weights.
- **testing workflow parameters** – parameters relating to diagnostic accuracy and time-to-diagnosis (e.g. time-to-obtain stool samples, time-to-transport samples to the laboratory, operating time for each testing option).
- **costs**

**Supplementary Table 3. 6 Secondary cases prevented, incremental QALY gains, incremental costs INMB and INMB range for HT over LTHT testing strategies compared to baseline results – sorted by lower and upper bound for each disease-related parameter**

**Supplementary Table 3. 7 Secondary cases prevented, incremental QALY gains, incremental costs INMB and INMB range for HT over LTHT testing strategies compared to baseline results – sorted by lower and upper bound for each testing workflow-related parameter**

**Supplementary Table 3. 8 Secondary cases prevented, incremental QALY gains, incremental costs INMB and INMB range for HT over LTHT testing strategies compared to baseline results – sorted by lower and upper bound for each cost parameter**

**Supplementary Table 3. 9 New secondary cases of CDI, incremental QALY gains, incremental costs INMB between HT meeting the minimum performance specifications at 15-minutes turnaround time and LTHT testing strategy for each structural scenario being explored. Scenarios highlighted in orange have the greatest impact on the cost-effectiveness outputs associated with the minimum performance specifications for HT at baseline.**

**Scenario analysis**

**Supplementary Table 3. 9** presents the results of the scenario analysis compared to baseline results. Based on the results of the scenario analyses, simulating a typical UK District Hospital (S4) and increasing the availability of multiplex GI panels (S2) were found to have the greatest impact on the cost-effectiveness outputs associated with the minimum performance specifications for HT at 15-minute turnaround time.

The following subsections describe the impact of each structural scenario on clinical- and cost-effectiveness outputs associated with the minimum performance specifications at 15-minute turnaround time, separately.

**S.1 – Alternative de-escalation protocol for patients confirmed with CDI**

Simulating a more rapid de-escalation protocol resulted in HT yielding a higher INMB (£2,990) compared to the baseline results. Releasing patients confirmed with CDI upon symptoms resolution (rather than at the end of hospital stay) increased the availability of single rooms thereby reducing the number of new secondary cases in the general ward – across both testing strategies. Both testing strategies thus yielded lower incremental QALY lost due to secondary cases and therefore higher incremental QALY gains (0.04 compared to 0.02 at baseline). Under the LTHT testing strategy, however, patients experienced a longer turnaround time to yield final diagnosis which, in turn, led to worse clinical outcomes and thereby lower QALY gains (47.92) versus the HT testing strategy (47.96).

While the isolation costs were lower for both testing strategies under this scenario (£2,094,637 and £2,059,311 for HT and LTHT testing strategy, respectively) compared to baseline results (£2,396,752 and £2,310,641 for HT and LTHT testing strategy, respectively), general ward bed costs were higher. This reflected the longer LOS patients positive to CDI spent in general ward under S1 as opposed to single room isolation, as per the baseline scenario. The HT strategy, however, was associated with lower incremental isolation bed costs versus the LTHT testing strategy compared to baseline results. The marginal reduction in isolation costs therefore lead to higher incremental costs savings (-£2,093) between both strategies compared to the baseline results.

**S.2 – Increased availability of multiplex GI panels**

Adding one extra multiplex GI panel to test patients suspected with infectious diarrhoea led to a higher INMB for the HT against LTHT testing strategy (£150,796), compared to the baseline results.

Having a quicker confirmation of infectious diarrhoea due to other GI pathogens expedited the time-to-diagnosis for patients suspected with CDI. This increased the availability of single rooms as patients negative to CDI were more quickly de-isolated compared to baseline scenario. The faster assessment of GI pathogens allowed the full benefit of the HT rapid test to be realised, with HT in this scenario leading to fewer secondary cases within general ward over LTHT testing strategy (0.51) compared to baseline (3), which resulted in additional cost savings (-£148,887). An additional benefit of shortening the test turnaround time was the increased effectiveness of antibiotic treatment for CDI, which meant that patients positive to CDI also experienced better short-term clinical outcomes (0.10 QALY gain vs. standard care).

**S.3 – Reduced availability of single rooms in Teaching Hospital**

Reducing the number of single rooms available in a typical UK Teaching Hospital resulted in HT yielding a higher INMB compared to baseline results (£5,829). Both testing strategies yielded more new secondary cases, lower QALY gains and higher costs in this scenario compared to baseline results. In the context of a reduced availability of single rooms, the HT yielded fewer incremental secondary cases over LTHT testing strategy (2.86) compared to baseline results (3) as patients received a final diagnosis for CDI in a more timely manner as opposed to the comparator testing strategy. As the HT prevented more secondary cases within the general ward, this led to higher incremental QALY gains (0.03) and incremental cost savings (-£5,276) compared to baseline outputs.

**S.4 – UK District Hospital**

Simulating a typical UK District Hospital led to a higher INMB for HT over the comparator testing strategy compared to baseline (£153,899). Both testing strategies yielded fewer new secondary cases in the general ward, higher QALY gains and lower costs.

Although a typical UK District Hospital was associated with fewer single rooms where to isolate patients suspected and confirmed with CDI, under S4 a reduced number of patients were tested for CDI thereby there was a reduced demand for single rooms compared to UK Teaching Hospitals. This, in turn, resulted in fewer new secondary cases and thereby higher QALY gains. HT yielded fewer comparative secondary cases for CDI (-1.30) over LTHT testing strategy compared to baseline output (3), and led to higher incremental QALY gains (0.13) and lower incremental costs (-£151,263).

**S.5 – Alternative distributions for time-to-event parameters**

Applying the second-best distribution for the time-to-event patient parameters (e.g. LOS, duration of symptoms), resulted in an increase of the cost-effectiveness output for HT compared to LTHT testing strategy (INMB equal to £149,930).

Across both testing strategies, simulating the second-best distribution to time-to-event patient parameters resulted in fewer new secondary cases, higher incremental QALY gains and higher costs. The HT strategy yielded better incremental clinical benefits (i.e. fewer new secondary cases, higher QALY gains) over the comparator testing strategy, compared to baseline results. In this context, HT was associated with a greater reduction in new secondary cases, leading to higher incremental QALY gains compared to LTHT testing strategy.

Applying the second-best distribution had the following downstream implications:

- **lower average LOS for patients confirmed with CDI, and reduced shorter right tail of high LOS values (i.e. fewer extreme high values)** – this, in turn, led to lower isolation costs associated with both testing strategies (£2,228,105 for HT and £2,206,964 for the LTHT testing strategy) compared to the respective baseline values (£2,396,752 and £2,310,640, respectively);
- **higher average LOS for patients negative to CDI on average but shorter right tail** – overall, this led to a higher general ward bed costs associated with both testing strategies (£14,596,509 and £ 14,878,135 for HT and LTHT testing strategy) compared to baseline values (£13,617,912 and £13,617,790 at the base case). The reduction in isolation costs were greater for HT compared to the LTHT testing strategy – the economic benefit of reducing the LOS for patients negative to CDI was offset by a longer turnaround time to receive confirmation of non-infectious diarrhoea under LTHT testing strategy, thereby leading to higher isolation costs for the LTHT testing strategy compared to HT.

1. Despite HT leading to more secondary cases compared to LTHT testing strategy, at 15-minutes turnaround time HT yielded QALY gains as faster testing strategies improved short-term patient health (e.g. higher probability of clinical cure, shorter LOS) to a greater extent compared to testing strategies with slower turnaround time. The higher individual QALY gains associated with faster test turnaround time offset the reduction in QALY gains associated with an increased infection spread. [↑](#footnote-ref-1)
2. Across 70 model replications, the size of the cohort entering the model on average 539 patients. [↑](#footnote-ref-2)
